# Fast relocking and afterslip-seismicity evolution following the 2015 Mw 8.3 Illapel earthquake in Chile

**DOI:** 10.1038/s41598-023-45369-9

**Published:** 2023-11-09

**Authors:** Joaquín Hormazábal, Marcos Moreno, Francisco Ortega-Culaciati, Juan Carlos Báez, Carlos Peña, Christian Sippl, Diego González-Vidal, Javier Ruiz, Sabrina Metzger, Shoichi Yoshioka

**Affiliations:** 1 Department of Geophysics, Faculty of Physical and Mathematical Sciences, University of Chile, Santiago, Chile; 2 Department of Structural and Geotechnical Engineering, Pontificia Universidad Católica, Santiago, Chile; 3 Millennium Institute of Oceanography, IMO, Concepción, Chile; 4 Data Observatory Foundation, ANID Technology Center No. DO210001, Santiago, Chile; 5 Centro Sismológico Nacional, Facultad de Ciencias Físicas y Matemáticas, Universidad de Chile, Santiago, Chile; 6 Helmholtz Centre Potsdam, GFZ German Research Centre for Geosciences, Potsdam, Germany; 7 Institute of Geosciences, Ruhr University Bochum, Bochum, Germany; 8 Institute of Geophysics of the Czech Academy of Sciences, Prague, Czech Republic; 9 Department of Earth Science, University of Concepción, Concepción, Chile; 10 Research Center for Urban Safety and Security, Kobe University, Rokkodai-cho 1-1, Nada Ward, Kobe, 657-8501 Japan; 11 Department of Planetology, Graduate School of Science, Kobe University, Rokkodai-cho 1-1, Nada Ward, Kobe, 657-8501 Japan

**Keywords:** Geophysics, Tectonics

## Abstract

Large subduction earthquakes induce complex postseismic deformation, primarily driven by afterslip and viscoelastic relaxation, in addition to interplate relocking processes. However, these signals are intricately intertwined, posing challenges in determining the timing and nature of relocking. Here, we use six years of continuous GNSS measurements (2015–2021) to study the spatiotemporal evolution of afterslip, seismicity and locking after the 2015 Illapel earthquake ($M_w$ 8.3). Afterslip is inverted from postseismic displacements corrected for nonlinear viscoelastic relaxation modeled using a power-law rheology, and the distribution of locking is obtained from the linear trend of GNSS stations. Our results show that afterslip is mainly concentrated in two zones surrounding the region of largest coseismic slip. The accumulated afterslip (corresponding to $M_w$ 7.8) exceeds 1.5 m, with aftershocks mainly occurring at the boundaries of the afterslip patches. Our results reveal that the region experiencing the largest coseismic slip undergoes rapid relocking, exhibiting the behavior of a persistent velocity weakening asperity, with no observed aftershocks or afterslip within this region during the observed period. The rapid relocking of this asperity may explain the almost regular recurrence time of earthquakes in this region, as similar events occurred in 1880 and 1943.

## Introduction

Knowledge of the spatiotemporal evolution of kinematic processes at the subduction interface is essential for enhancing our understanding of the mechanisms underlying stress accumulation and release throughout the seismic cycle of major earthquakes. We know that during the interseismic period, the plate interface is heterogeneously locked^1–3^, with certain segments fully locked and others undergoing aseismic slip, resulting in variable strain accumulation along strike and depth. These variations in the degree of locking appear to influence the characteristics of future earthquakes, as evidenced by a correlation between areas of observed coseismic slip and patchworks of geodetically-determined interseismically locked zones for the most significant earthquakes of the past nearly two decades^1–4^. Therefore, the degree of locking when combined with historical earthquake data is a valuable tool for estimating the slip deficit, providing crucial information about the potential location and magnitude of future earthquakes. However, our knowledge of the temporal variations in locking is limited by the absence of long-term geodetic records that cover the entire seismic cycle, which can span from tens of years to centuries. This limitation hampers our ability to accurately assess slip deficits in subduction zones.

After large earthquakes, surface displacement occurs in the opposite direction compared to the interseismic period, exhibiting a gradual decay in the rate of displacement over time. These observations were first documented in Japan during the mid-20th century^5,6^. Later, with the advent of space geodesy, these effects have been extensively documented^7–11^. The postseismic processes are time-dependent, and their magnitude and relaxation time are controlled by the magnitude of the earthquake and the rheology of the fault- and lithosphere-asthenosphere-system^7,11,12^. In addition, postseismic deformation processes are influenced by the stress state of the surrounding volume and the evolution of stresses on the fault^13,14^. Rapidly decaying postseismic deformation (lasting days or years) in the near-field of the rupture can result from fault afterslip caused by the frictional response of the subduction interface^8,15^. Larger-scale processes with short- and long-term effects on the deformation field (lasting from days to tens of years) include viscoelastic relaxation of the upper continental and oceanic mantle^9,10,16^, which stresses the upper plate and results in trenchward displacement over a wide inland region^17^. Other processes that can contribute to postseismic deformation include crustal faulting in the upper plate^18^, and poroelastic deformation caused by fluid flow in response to coseismic stress changes within the pore space^19^. Previous work^20,21^ has shown how difficult it is to distinguish these processes in geodetic observations because they often act simultaneously.

When broken by a large earthquake, certain sections of the fault undergo frictional restrengthening (healing), resulting in relocking processes, while other sections continue to experience a combination of seismic (aftershocks) and aseismic (afterslip) slip. This complex behavior poses challenges in accurately identifying the exact moment of relocking, leading to ongoing debates regarding the rate of fault healing and the timing of relocking. Some laboratory experiments and geodetic modeling suggest that the plate interface can rapidly recover its interseismic locking state after a large slip, with recovery times ranging from instantaneous to a period of one year^21–23^. In contrast, experimental data from samples taken from the Hikurangi margin, which experiences continuous slow earthquakes, indicate near-zero healing rates^24^. Consequently, there is no widespread agreement on the timing and controlling factors of healing, primarily due to the limited number of observations documenting the relocking process and the challenges involved in extrapolating from experimental data. Additionally, the challenge of estimating post-earthquake slip hampers our understanding of the relationship between aftershocks and afterslip. While it has been proposed that aftershocks are triggered by stress perturbations resulting from afterslip^25^, the considerable uncertainty in afterslip models^26^ leaves the connection between aftershocks and afterslip unclear. Obtaining new evidence on the timing of the transition from rapid coseismic to slower afterslip and relocking is crucial for assessing the interaction between different slip modes and their contribution to the overall slip budget in the seismic cycle.

**Figure 1:**
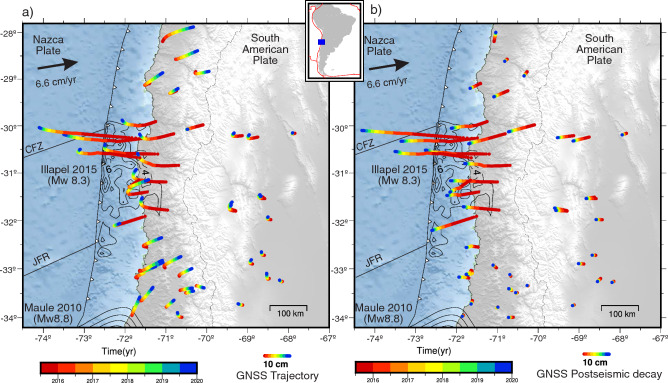
Map of the study area showing time colored cumulative postseismic ground displacements recorded at continuous GNSS stations during four years after the 2015 Illapel earthquake. Black contours show the coseismic slip model of the Illapel^32^ and Maule^43^ earthquakes, with contour intervals of 2 m. (**a**) Evolution of the horizontal trajectory at each station, considering the postseismic decay and the linear trend. (**b**) Trajectory of postseismic deformation alone. JFR is the Juan Fernandez Ridge, and CFZ is the Challenger Fracture Zone. The figure was created using GMT 6 (Generic Mapping Tools) software^44^.

In this study, we present evidence that the rupture zone of the 2015 Illapel earthquake, with a moment magnitude ($M_w$) of 8.3 in Chile, has been fully relocked since, at most, the third year after the event. We analyze and model data from 51 continuous Global Navigation Satellite System (GNSS) stations (Fig. 1, Figs. S1, S2) spanning the first 6 years (2015–2021) after the 16 September 2015 Illapel earthquake to characterize postseismic deformation, its relationship to seismicity, and the degree of current plate locking around the rupture zone. The Illapel region in north-central Chile is located at the plate boundary system of the Nazca and South American plates (Fig. 1). This region is characterized by intense seismic activity, which has increased over the last 20 years. The coseismic source of the 2015 Illapel earthquake has been extensively studied^27–32^. This earthquake ruptured an area of $\sim$200$\times$100 km, $\sim$300 km north of the 2010 Maule earthquake^2^, and caused total slip peaks of 6–9 m^27,31,32^ (Fig. 1). Earlier earthquakes similar to 2015 occurred in 1943 and 1880^33^, suggesting some regularity in the accumulation and release of seismic energy in this segment.

Previous studies have estimated the early postseismic deformation of the Illapel earthquake, considering short time windows of 1 and 11 days^31^, 26 days^34^, 43 days^29^, 45 days^35–37^, 60 days^38^, 74 days^39^, and 10 months^40^. These studies mainly investigated afterslip processes; only Guo et al.^35^ included linear viscoelastic relaxation and afterslip models, while Yang et al.^37^ also considered poroelastic effects on afterslip distributions. Most of these studies agree on two main afterslip patches located along the northern and southern edges of the coseismic rupture (Fig. 1), separated by the deepest part of the coseismic rupture. Higher afterslip is generally observed in the northern patch, and afterslip during the first months after the earthquake is equivalent to 12% to 13% of the coseismic moment^29^. Frank et al.^40^ suggests that the afterslip following the mainshock rupture is the main driver of aftershocks. The purpose of this study is to take a step forward, by investigating the spatio-temporal slip behavior of the megathrust constrained by continuous GNSS data. We do so by implementing a 4D forward numerical model, and applying the least squares inversion with Equal Posterior Information Condition (EPIC) Tikhonov regularization^41^ to robustly resolve the afterslip and locking degree. Finally, we updated the seismicity catalog of north-central Chile of Sippl et al.^42^ to cover our entire observation period and compared it with the spatiotemporal evolution of afterslip and plate locking.

## Results

**Figure 2:**
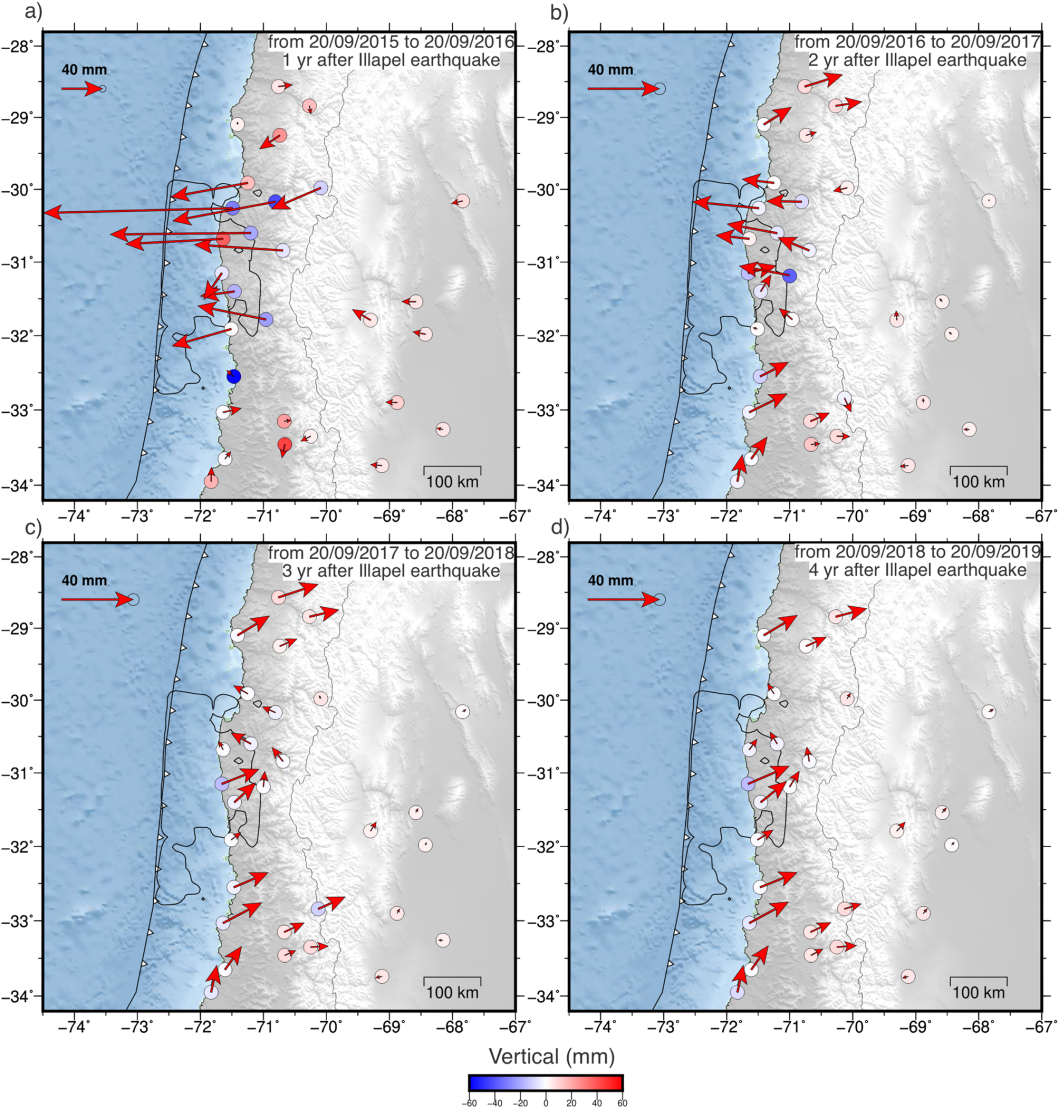
Annual cumulative horizontal and vertical displacements at GNSS stations between 20 September 2015 and 20 September 2019. Panels **a)**-**d**) show predictions of the linear trend plus postseismic decay at the stations during years one to four after the earthquake, respectively. Black contour shows the area where coseismic slip is greater than 1 m^32^. Colored circles show vertical displacements. Note the different scale of the horizontal vectors in panel **a**). We observe the evolution of ground displacements and their change in direction from a trenchward motion - panel **a**) - to a landward movement - panel **d**) - being the latter the general behavior four years after the earthquake. The figure was created using GMT 6 (Generic Mapping Tools) software^44^.

### Spatiotemporal evolution of the surface displacement field and seismicity

The curvature in the spatial path of ground motions in the years following a large earthquake such as 2015 Illapel (e.g., $M_w \sim$ 8) (Fig. 1a) is a combination of transient postseismic processes and plate relocking signal. Initially, postseismic deformation (including relaxation, relocking and locking in adjacent zones) dominates, but it decays rapidly in the nearshore areas where the coupling signal begins to prevail, as observed in the interseismic rotation of the displacement vectors (Fig. 2). All stations near the rupture zone show a rapid westward movement immediately after the Illapel earthquake (up to 18 cm in the first year), which then gradually slows down in the following years, producing a clockwise rotation of the horizontal displacements until they reach the interseismic direction. The segments north and south of the Illapel rupture zone are mainly affected by a short postseismic deformation, which quickly transitions after one year to an interseismic phase with movement in the direction of plate convergence.

During the first year, the GNSS stations show significant subsidence ($\sim$6 cm) above the rupture downdip limit and localized uplift of about 4.6 cm at the coast. Gentle uplift is also observed in the Andean mountain range and backarc, forming a long-wavelength lithospheric flexure pattern that decreases with time (Fig. 2). In the second year after the earthquake, GNSS stations on the coast near the center of the rupture zone reverse the direction of their horizontal motion toward the interseismic direction (northeast). This change indicates the beginning of the predominance of relocking over the postseismic signal in the near field. Three and four years after the earthquake, the ground surface continues to move interseismically in the central part of the rupture zone (31$^{\circ }$S–31.5$^{\circ }$S), but is surrounded by areas with smaller displacement (Fig. 2c,d). Over 4 years after the earthquake, the largest cumulative displacements toward the trench reach $\sim$30 cm (Fig. 1a). In this period, the postseismic deformation field is mainly concentrated in the Chilean forearc, between $\sim$29.8$^{\circ }$S and 32.2$^{\circ }$S, around the rupture zone, without significantly affecting the backarc.

**Figure 3:**
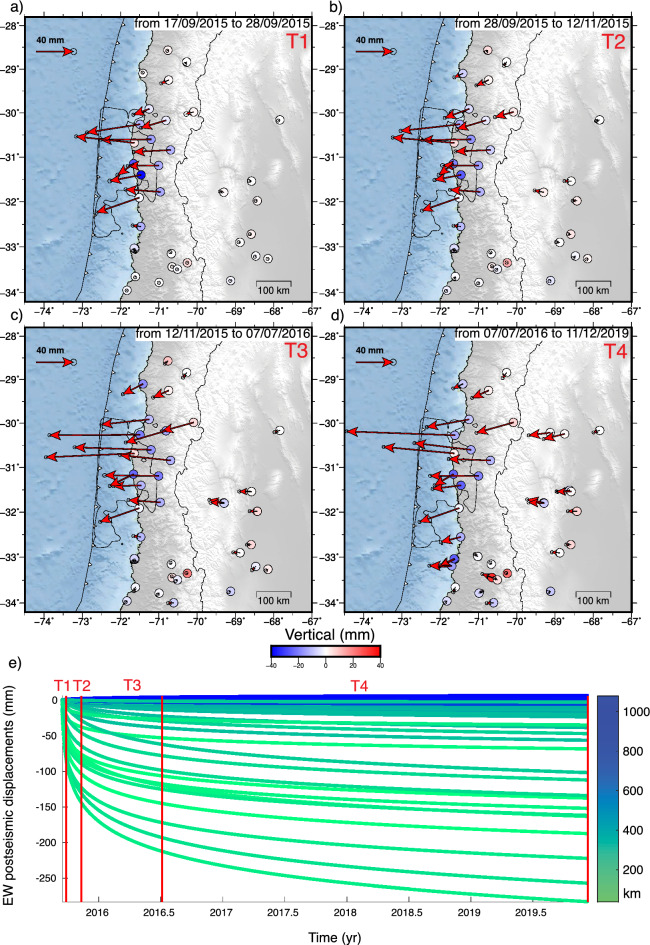
Cumulative postseismic displacements during the “equal-amplitude” (geometric) time windows T1 (**a**), T2 (**b**), T3 (**c**), and T4 (**d**), with duration of 11, 56, 294, and 1546 days, respectively. Horizontal and vertical displacements are shown as arrows and colored circles, respectively. Black contour line shows the area where coseismic slip is greater than 1 m^32^. (**e**) The cumulative postseismic horizontal (eastward) displacements of the GNSS station network as a function of time. The red lines indicate the temporal boundaries of the four geometric windows defining the analyzed periods of postseismic deformation. The color of each displacement curve is based on the distance between the station and the 2015 earthquake epicenter. The displacements have similar amplitude ranges at all temporal windows. The figure was created using GMT 6 (Generic Mapping Tools) software^44^.

We isolate the logarithmic decay components from the trajectory models, which represent postseismic deformation processes (mainly afterslip and viscoelastic relaxation) until the end of 2019 at each station (Figs. 1b,  3). The postseismic ground motion is rapid in the first year after the earthquake, reaching more than 20 cm. Then, it becomes noticeably slower over the rest of the observed period, reaching a cumulative maximum of $\sim$27 cm (Fig. 3 and Fig. S3). To model the postseismic deformation mechanisms, we divided the observation time into four time windows, T1–4, with durations of 11, 56, 294, and 1546 days to obtain similar amplitude displacements and thus maintain the signal-to-noise ratio (Fig. 3). The separation of the postseismic signal at each GNSS station into time windows with displacements of similar amplitude, allows quantifying the change in the global deformation pattern over time, i.e., the relative behavior between the near and far field. Thus, we can characterize postseismic deformation patterns caused by postseismic relaxation of the mantle that affects mostly the far field and by afterslip, whose signal is concentrated near the rupture. To calculate the duration of the time windows T1–T4, we used only GNSS time series covering the full observation period, including the first few days after the earthquake, when the most significant displacements were recorded. Once these windows were set, we calculated the postseismic ground displacements of the GNSS data, selecting only the stations with more than 95% of the data in that window.

The stations near the coast around the rupture zone have the largest horizontal postseismic displacements (cumulative displacements greater than $\sim$7 cm in each window). Stations in the backarc region show small but resolvable horizontal displacements ($\sim$1 cm cumulative in T1, T2, and T3). Only in T4 (which spans a much longer time than the other windows) the cumulative displacements in the backarc exceed $\sim$3 cm, indicating that the decay time is longer in the far field than near the rupture zone. The horizontal displacements change direction at the center of the rupture zone, a pattern that suggests the development of two afterslip patches. The stations show continuous subsidence near the coast (>2.5 cm accumulated in each time window) and localized uplift inland of the maximum coseismic slip ($\sim$1 cm accumulated per time window).

**Figure 4:**
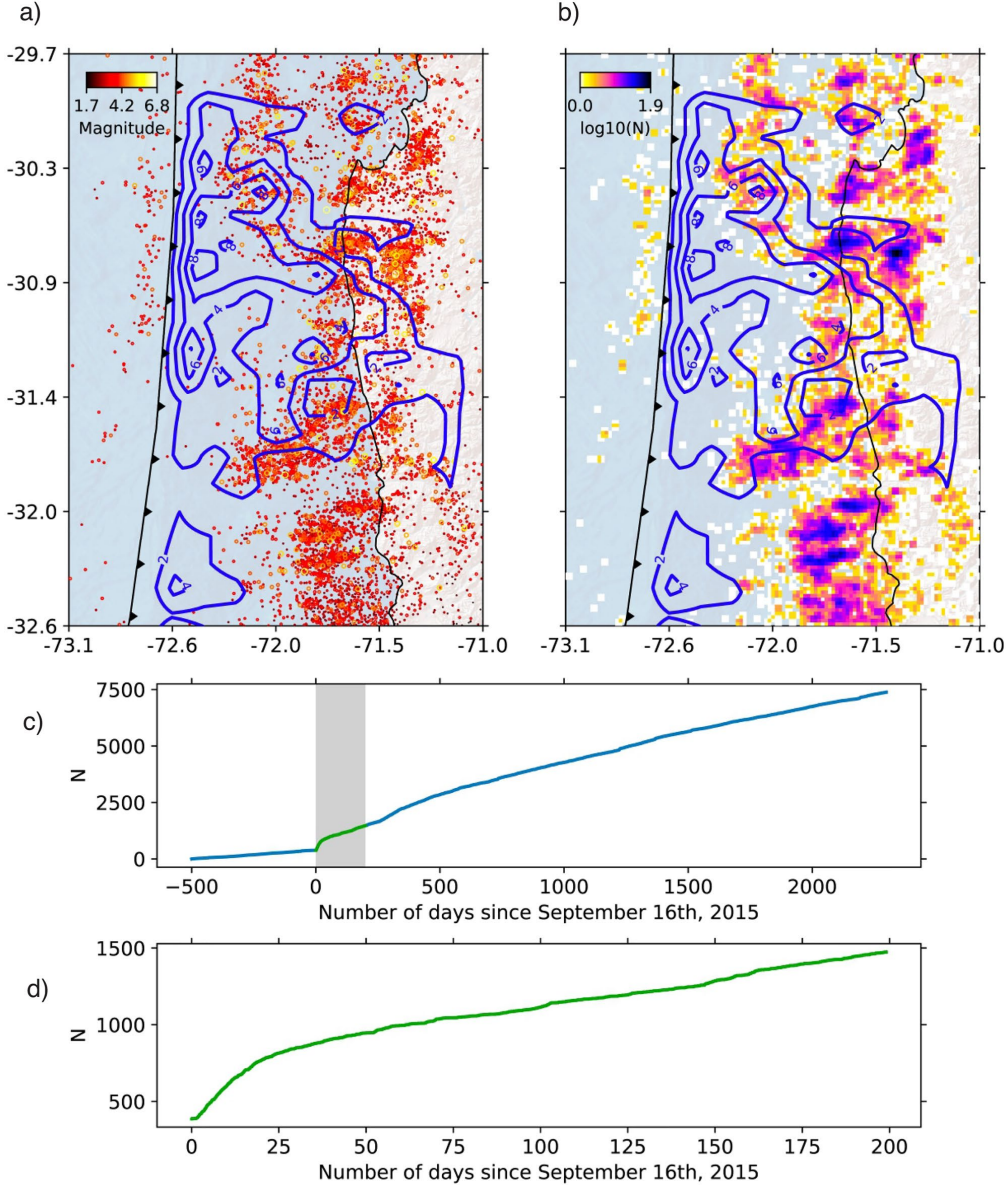
Catalog of microseismicity from 1 April 2014 to 31 December 2021. (**a**) Map view plot of epicenters; circle sizes are scaled and colored by magnitude. (**b**) Plot of seismicity density. In (**a,b**), the contour lines of coseismic slip^32^ are shown at intervals of two meters. (**c,d**) Cumulative events in the region of the map view for the entire time interval covered by the catalog (**c**) and for 125 days after the Illapel earthquake (**d**). The figure was created using Cartopy (https://scitools.org.uk/cartopy).

The seismicity catalog, which covers the time interval from 1 April 2014 to 31 December 2021, shows that seismic activity surrounds the rupture zone of the 2015 earthquake. There is an increased occurrence of seismic events directly below the rupture area, as well as at shallower depths to the north and south (Fig. 4). Conversely, the area that experienced rupture during the main shock displays notably lower seismic activity. Exponential decay of the aftershock rate occurs until $\sim$50 days after the Illapel earthquake, followed by a relatively constant rate of background seismicity. We do not observe clear changes in the spatial distribution of seismicity between the early aftershock sequence and the later parts of the earthquake catalog, which can be considered background activity.

### Afterslip and locking degree distributions

We constrain afterslip, using the accumulated postseismic surface displacements in each of the geometric time windows T1-T4. We estimate the afterslip distribution at the plate interface using a combination of a 3D geomechanical model and an inversion approach, similar to the method presented by Peña et al.^45^. Accordingly, within each individual geometric time window, we subtract the predicted postseismic decay based on a nonlinear viscoelastic relaxation model^45^ (Figs. S4, S5) from the measured displacements. By applying this correction within each geometric time window, we derive the distributions of afterslip. We performed afterslip inversions constrained by postseismic decay displacements, as well as those corrected by the effects of mantle viscoelastic relaxation (Fig. S6). To determine our preferred afterslip models, we use the L-curve method^46^ (Fig. S7). All afterslip inversions fit well to the accumulated displacements of each time window (Figs. S8, S9).

**Figure 5:**
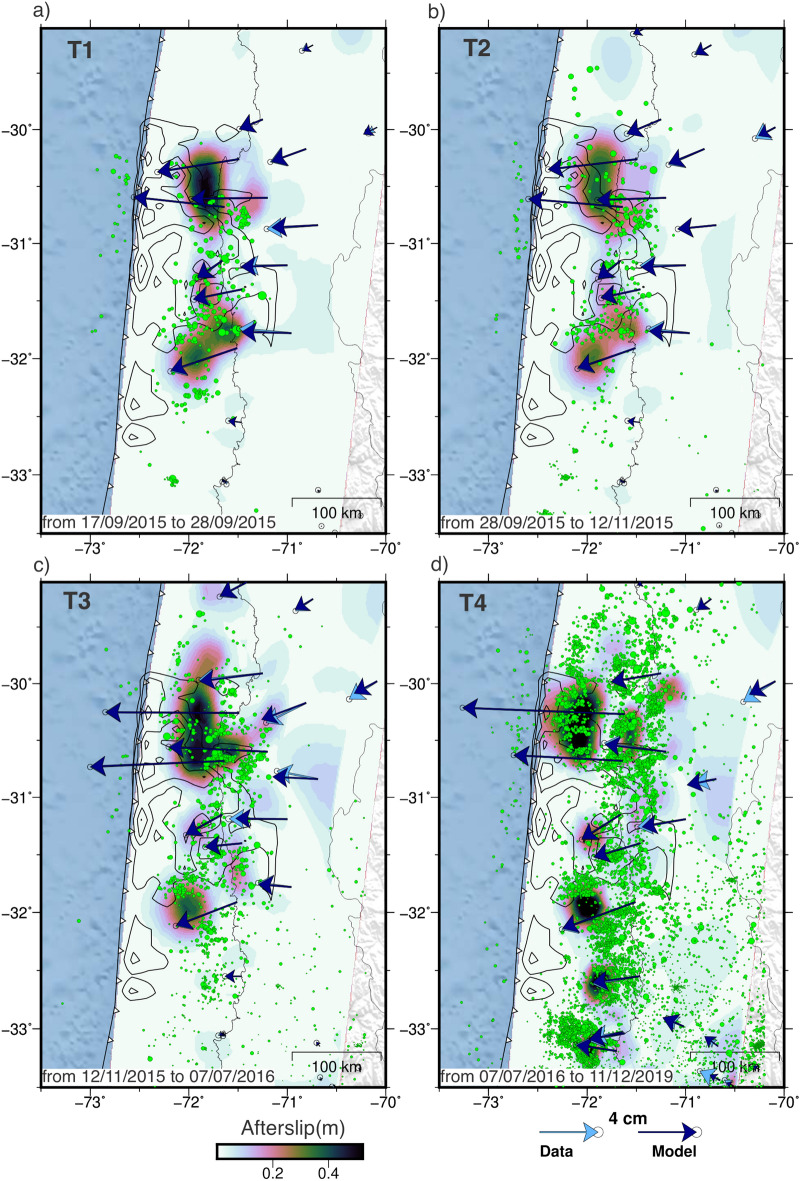
Modeled cumulative afterslip distribution for each time window. (**a**–**d**) Afterslip distributions for T1 (**a**), T2 (**b**), T3 (**c**), and T4 (**d**). The gray lines represent the Illapel 2015 slip distribution 2 meter contours^32^ and the green dots the seismicity for each time window. The light blue and dark blue vectors show the observed and modeled horizontal displacements, respectively. The figure was created using GMT 6 (Generic Mapping Tools) software^44^.

The viscoelastic model based on the coseismic slip of Tilmann et al.^27^ does not result in significant displacements (<2 cm) in the backarc region (Fig. S5). As a result, both the uncorrected displacements and those corrected using the Tilmann et al.^27^  coseismic slip-based viscoelastic model, exhibit large displacements in the backarc, which in turn lead to inferring afterslip at greater depths (Figs. S5,  S8). In contrast, the viscoelastic model based on the mainshock slip from Carrasco et al.^32^ predicts backarc displacements that are of similar magnitudes as the GNSS observations, exceeding 3 cm. By correcting the observations using the predictions of this viscoelastic model, we obtain afterslip distributions concentrated in the surroundings of the mainshock rupture (Fig. 5), which in turn results in a better fit to the data in the far field. Therefore, we focus our analysis on the latter model.

The afterslip distributions in the T1, T2, and T3 time windows show similar first-order features in the inversions of the data corrected for the predicted viscoelastic relaxation motions and in the uncorrected data. In these windows, afterslip consists of two separate segments, one in the north of the rupture zone (with higher magnitude) and one in the south, both at similar depths. In time windows T3 and T4, models based on uncorrected data increase the afterslip inferred at depths greater than 60 km, which may be an artifact due to the absence of the viscoelastic component in the modeling. This behavior is consistent with the increase in displacements predicted by the viscoelastic models in the backarc during periods T3 and T4. The afterslip of period T4 becomes patchy (Fig. 5d), with the main afterslip lobes splitting apart, consistent with a large diminishing of afterslip rate in that period.

Results from our preferred model (Fig. 5) show distributions of cumulative afterslip corresponding to moment magnitudes ($M_w$) of 7.3, 7.3, 7.4, and 7.5 for time windows T1 (11 days), T2 (56 days), T3 (294 days), and T4 (1546 days), respectively. The daily average of afterslip moment for T1, T2, T3, and T4 are $M_w$ 6.7, 6.2, 5.8, and 5.3, respectively. The northern afterslip patch has a maximum dislocation of 0.52 m, 0.38 m, 0.52 m, and 0.65 m at T1, T2, T3 and T4, respectively. The northern patch has a cumulative amplitude of 1.74 m and the afterslip has a magnitude $M_w$ 7.8 in the observed period. In the T1 time window, seismicity is mainly concentrated around the southern afterslip area. In T2 and T3, seismicity begins to surround the  regions with high afterslip, that exhibit no seismicity within. In T4, a larger number of events, like the afterslip, show a more patchy distribution, also accompanied with an increase in complexity of seismicity patterns that surround areas of high afterslip.

**Figure 6:**
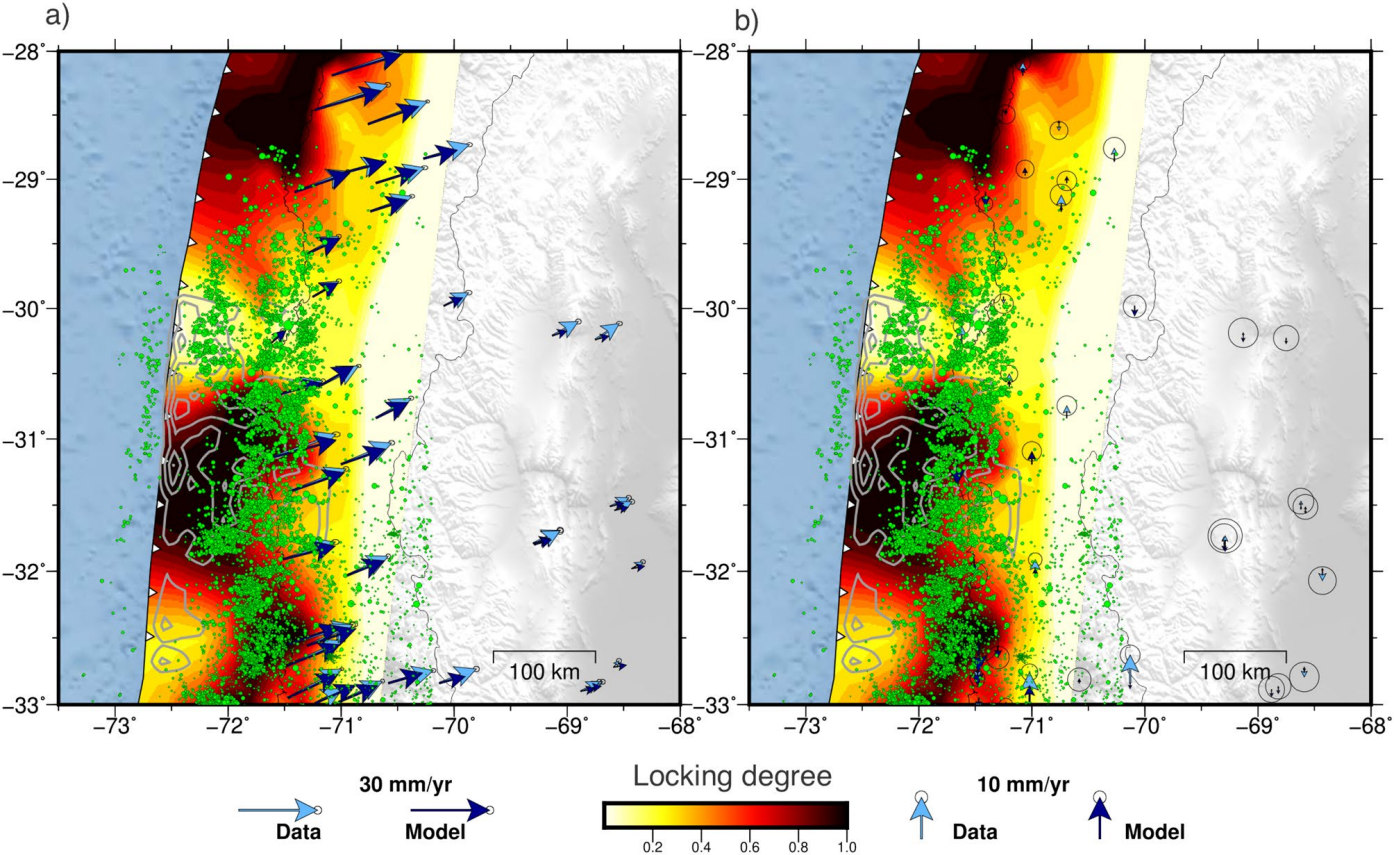
Degree of locking based on estimated secular velocities from 2018 to 2021. (**a**) Horizontal and (**b**) vertical GNSS secular velocities expressed in a stable South American reference frame. Light and dark blue vectors represent observations and locking model predictions, respectively. Green circles show the updated seismicity catalog^42^, including events up to 2021. Gray 2 meter contour lines represent the 2015 Illapel coseismic slip^32^. The figure was created using GMT 6 (Generic Mapping Tools) software^44^.

To obtain the velocities used to constrain locking, we analyze the time series from 2018 to 2020 due to the presence of postseismic effects, data gaps, and artificial offsets prior to that period, which may introduce a bias in the inferred velocities. The locking degree is then estimated using a method similar to Li et al.^47^, with the exception that we employ the same inversion method implemented for the afterslip distributions. Our best-fitting locking model reproduces the horizontal and vertical velocities between 2018 and 2021 quite well (Fig. 6, Fig. S10). Our analysis suggests that the rupture zone of the Illapel earthquake is highly locked between 2018 and 2020, with creeping zones located to the north and south of the rupture area. To the north of the rupture zone, there is an approximately 50 km long corridor of creep, which gradually increases its degree of locking north of 29°S, where the plate interface is highly locked offshore. South of 32°S, the model infers high locking in the deeper part of the seismogenic zone and creeping near the trench, which is an area that may not be well resolved by the inversion. Seismicity surrounds the highly locked zone and is concentrated in the creeping corridor.

**Figure 7:**
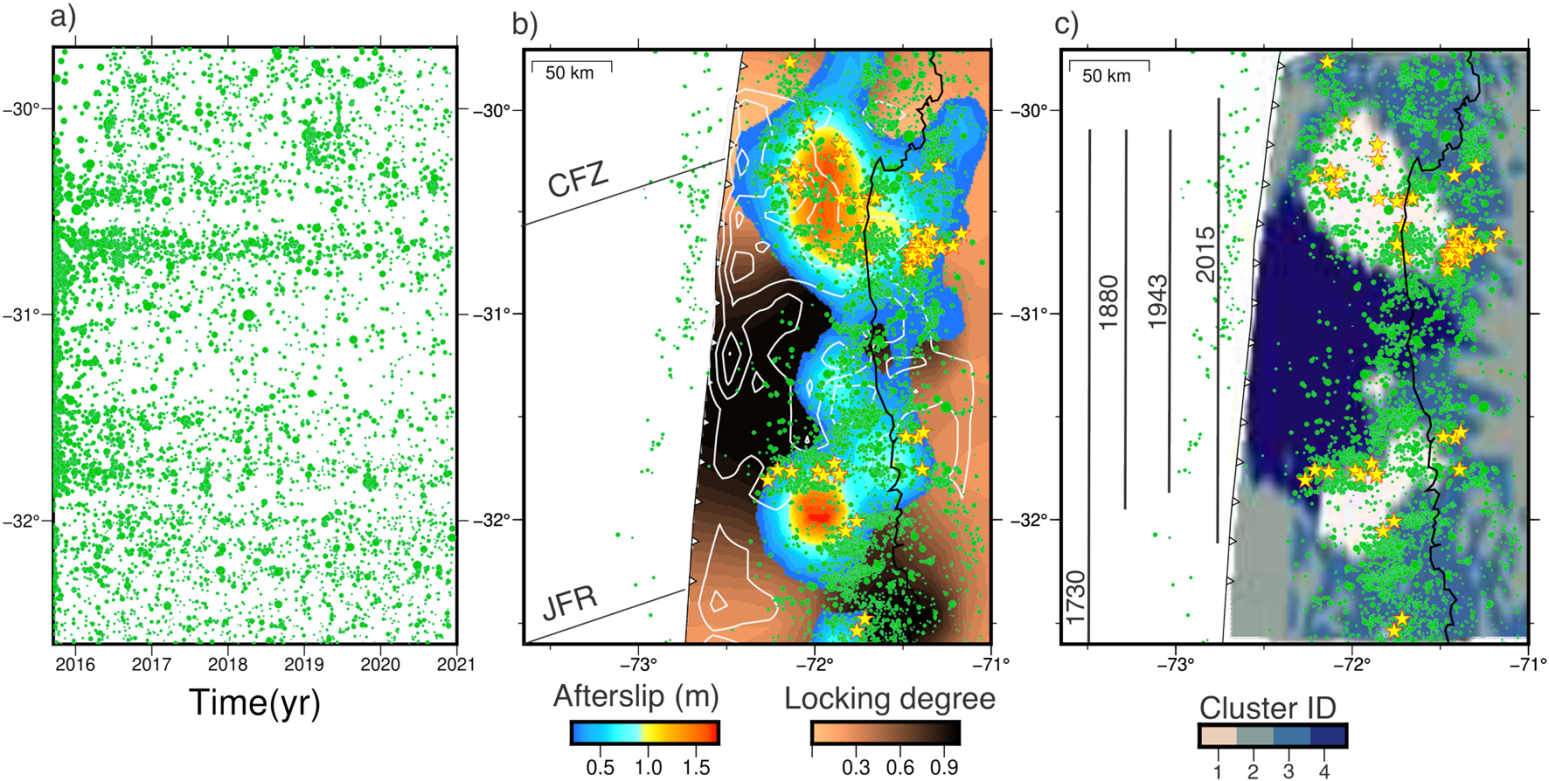
Comparison of the kinematic behavior of the plate interface (coseismic slip, afterslip, and locking degree) with the seismicity. (**a**) Temporal evolution of the seismicity along latitudes. (**b**) Accumulated afterslip between 17 September 2015 and 11 December 2019 and distribution of locking degree estimated from GNSS velocities between 2018 and 2022. JFR is the Juan Fernandez Ridge, and CFZ is the Challenger Fracture Zone. White contour lines represent the Illapel coseismic slip^32^. Green dots represent seismicity, yellow stars represent repeating earthquakes. (**c**) Distribution of earthquake clusters based on an analysis of spatial correlations between coseismic slip, afterslip, locking degree, and seismic moment distributions. Gray lines show estimated rupture lengths for historical and recent large earthquakes^33^. The figure was created using GMT 6 (Generic Mapping Tools) software^44^.

We performed a clustering analysis using the agglomerative clustering algorithm implemented in sklearn-scikit^48^ to investigate the spatial relationship between the distributions of coseismic slip, afterslip, locking, and seismic moment estimate to evaluate the kinematic behavior of slip at the megathrust (Fig. 7). We chose an optimal number of four clusters (Fig. S11), which gives a local minimum Bayesian Information Criterion (BIC). A larger number of clusters reduces the BIC values but overfits the data. Accordingly, four zones with distinct kinematics at the plate interface can be characterized by clustering analysis (Fig. 7c, Fig. S12). Cluster 1 groups the zones with high afterslip (average: 1.1 m), low coseismic slip (average: 2.2 m), moderate locking degree (average: 0.5), and high seismic moment estimate (average: 13.1 log(Nm)). Cluster 2 is located in areas of low locking and no seismicity, unaffected by the 2015 earthquake. Cluster 3 groups areas with high seismicity but low afterslip and moderate locking. Cluster 4 groups areas with low afterslip (average: 0.14 m), high coseismic slip (average: 4.6 m), moderate degree of locking (average: 0.9), and low seismic moment estimate (average: 6.7 log(Nm)).

## Discussion

This work presents a comprehensive analysis of the evolution of ground displacements and seismicity following the 2015 ($M_w\,8.3$) Illapel earthquake. Covering the period from 2015 to 2021, the study focuses on two specific aspects: the afterslip analysis from 2015 to 2019 and the locking estimation from 2018 to 2021. By examining these postseismic observations over a span of approximately 6 years, we gain valuable insights into the temporal and spatial patterns of viscoelastic deformation, afterslip, relocking, and their correlation with seismic activity. The analysis of the spatiotemporal evolution of ground motion reveals that the central region of the 2015 rupture zone exhibits what we refer to as reloading, the initial indications of the transition from postseismic extension to interseismic contraction dominating crustal deformation. As an indicator of a restarting interseismic strain accumulation in the upper crust, reloading serves as a proxy to evidencing that the central region of the 2015 rupture is the first zone to become relocked. Reloading, and thus relocking, are evident through the observed shift in displacement direction, transitioning from movement towards the trench to movement towards plate convergence during two years after the earthquake (Figs. 1 and  2). These findings are supported by the results obtained from the locking inversion, which indicate that the entire 2015 rupture zone is fully coupled during the period from 2018 to 2021. On the contrary, postseismic deformation, characterized by displacements towards the trench, predominates at the edges of the rupture zone and in the far-field backarc region (Fig. 3). This observation strongly indicates that the distribution of afterslip is concentrated around the seismic rupture zone (Fig. 5), while the viscoelastic relaxation of the mantle induces postseismic deformation in the backarc. These patterns of afterslip closely align with those observed in previous studies based on early postseismic displacements^39^. The viscoelastic relaxation and afterslip induced by the Illapel earthquake exhibit distinct decay rates over time. In period T4, the far-field horizontal displacements show a higher magnitude in comparison to earlier time windows (Fig. 3). This observation implies that the afterslip may have declined during the analyzed period, while the effects of viscoelastic relaxation persist and continue to impact the far field over an extended duration.

Compared to the vertical pattern of the 2010 Maule earthquake, whose uplift mainly affected the Andes^17^, the post-Illapel uplift is concentrated only on the coast near the rupture center (Figs. 3 and  4), which is an area surrounded by subsidence. This vertical deformation pattern suggests that afterslip is the dominant process in nearshore ground motion, since megathrust inverse slip beneath the coast can drive uplift along the coast. Therefore, probably due to the smaller magnitude of the Illapel earthquake, it induces deformation dominated by viscoelastic processes mainly in the volcanic arc and backarc zones (far field), and by afterslip in the near field.

The afterslip distributions in the first three time windows are similar, consisting of two main afterslip zones, one to the north (larger in size and slip) and the other to the south of the rupture zone (Fig. 5). The amount of afterslip is similar in all time intervals, but in the first two time windows, the number of seismic events is relatively small compared those in the T3 and T4 windows. Therefore, postseismic slip in the first 67 days is predominantly aseismic. In the fourth time window (T4), we see that afterslip breaks up into smaller areas, resembling a patchwork similar to the spatial distribution of seismicity in that period. The northern patch propagates into the trench, triggering seismicity updip of the aseismic slip (Fig. 5d). Previous studies also suggest that the afterslip from the northern patch propagates toward the trench^11^. The number of seismic events decays rapidly in the first 50 days after the earthquake. In addition, the average daily moment magnitudes decreases significantly from $M_w$ 6.7 in T1 to $M_w$ 5.3 in T4. This, together with the reversal of the direction of horizontal displacements to the east during three years after the earthquake (Fig. 2) and the disintegration of the two main afterslip patches in T4 into smaller zones, suggest that afterslip is waning quickly. Thus, four years after the Illapel event, the deformation field is becoming dominated by interseismic contraction.

In all periods analyzed, seismicity and repeater earthquakes tend to concentrate at the edges of the afterslip patches, while they are absent in areas of high afterslip (Figs. 5 and  7). The core of the afterslip patches remains aseismic throughout the observation period (Fig. 7), confirming their aseismic behavior. This implies that the aftershocks might be a result of tractions generated by the movements of these patches, suggesting that afterslip drives aftershocks^25^. The distribution of Illapel afterslip and seismicity never propagates into the zone of maximum coseismic slip, which is consistent with afterslip models of other earthquakes where afterslip surrounds coseismic ruptures, e.g, 2010 Maule^21,49^, 2011 Tohoku ^50^, and 2005 Sumatra^8^. Earthquakes of $M_w<8.5$ tend to produce relatively little afterslip, which decays rapidly. The percentage of the moment magnitude of afterslip relative to the main earthquake is $\sim$20.8% over four years for the Illapel event, which is consistent with the afterslip magnitude of similar sized events, such as the 1995 $M_w$ 8.1 Antofagasta earthquake (<20% in 1 year)^51,52^ and the 2007 $M_w$ 8.0 Pisco earthquake (7–28% in 1.1 year)^53^.

The distinct kinematic behavior and distribution of seismicity in the Illapel region megathrust suggests that the subduction interface is frictionally heterogeneous (Fig. 7c). It is composed of patches exhibiting seismic behavior (highly locked with high slip during earthquakes, cluster 4) and aseismic behavior (constant or episodic slip acting as a rupture barrier during large earthquakes, concentrating afterslip, cluster 1), as well as patches displaying dual behavior that are moderately coupled and concentrate background seismicity (cluster 3). Thus, the region of cluster 4 in Fig. 7c behaves as a persistent velocity-weakening asperity that may have ruptured in a similar manner during the 1880, 1943, and 2015 earthquakes (Fig. 7)^33^. Taking into account the recurrence interval of approximately 60-70 years for the previous two characteristic earthquakes in this area, as well as the evident indication of fault locking through surface displacements observed during 3 to 5 years after the 2015 earthquake, we can infer a rapid relocking within the seismic cycle. Consequently, the section of the plate boundary that exhibited significant locking before the 2015 earthquake^27^ rapidly reestablished its locked state following the event. The high degree of locking exhibited by this asperity prior to the 2015 Illapel earthquake, along with its rapid reattachment, suggests that interseismic coupling in this asperity is likely to remain high and consistent throughout the entire interseismic period.

The postseismic afterslip represents the response of the low-locked parts of the fault to the coseismic stress perturbation in a zone governed by a velocity-strengthening rheology (cluster 1). The kinematics of the zone appear to be related to permanent frictional properties due to subduction of the Challenger Fracture Zone and the Juan Fernandez Ridge (Fig. 7b). The subduction of these oceanic features may induce high pore fluid pressures^54^, geometric complexities^55^, and different frictional properties^56^ that can act as barriers to the rupture propagation of large earthquakes in the region. The megathrust region ruptured by the 2015 ($M_w 8.3$) Illapel earthquake seems to be capable of rapidly regaining frictional resistance. Therefore, we suggest it behaves as a persistent frictional feature that accumulates elastic energy over 60-70 years, generating the characteristic type of large earthquakes in the region ($M_w$ $\sim$8) at almost regular recurrence times (1848, 1943, and 2015).

## Methods

### GNSS time series analysis

The continental side of the Illapel rupture is well-covered by continuous GNSS stations^57^, which monitor 3-D surface motions from the coastline (only $\sim$ 80 to 100 km away from the trench) to the Argentina far field (>1000 km away from the trench, Fig. 1 and Fig. S1). We analyzed daily GNSS time series processed at the Nevada Geodetic Laboratory (NGL)^58^ from 17 September 2015 to 31 December 2020. We selected GNSS stations with sufficient temporal coverage (i.e., more than two years of continuous observations), yielding 51 stations that are well distributed in both the near and far field (Figs. S1,  S2). We use the NGL time series in the International GNSS-14 Service Reference Frame (IGS14)^59^. To account for the rigid-body rotation of South America, we transformed the estimated horizontal displacements and velocities to a reference system with respect to the stable part of the South American plate by subtracting the angular velocity described by the Euler vector of 21.44$^{\circ }$S, 125.18$^{\circ }$W, 0.12$^{\circ }$/Myr^60^.

GNSS time series primarily reflect a sum of tectonic processes, such as coseismic jumps, interseismic velocities, transient signals (e.g., postseismic motions and slow earthquakes), along with components related to seasonal oscillations (e.g., hydrologic forcing), instrumental failures (e.g., antenna replacement) and instrumental noise^61^. We use a trajectory model^61^ to describe the motion of a GNSS station and characterize the postseismic decay and secular velocities. This model decomposes the motion *x*(*t*) on each direction (i.e., east, north, up) of a GNSS station into four components as

1 $$\begin{aligned} x(t)=\overbrace{A + v(t-t_R)}^{\text {(1) secular}}+ \overbrace{\sum ^{n_i}_{i=1}B_i H(t-t_i)}^{\text {(2) jumps}} + \overbrace{\sum _{j=1}^{n_{eq}} C_jlog\left( 1+\frac{t - t_{eq_j}}{\tau }H(t-t_{eq_j})\right) }^{\text {(3) postseismic}} + \overbrace{ \sum ^{2}_{k=1}\left[ D_k cos(2\pi \frac{t}{T_k})+ E_k sin(2\pi \frac{t}{T_k})\right] }^{\text {(4) seasonal}} + \ \xi (t) \end{aligned}$$

where the different terms of the model correspond to: (1) a linear component representing secular deformation processes – e.g., interseismic velocity *v *— with respect to a reference time $t_R$; (2) subdaily jumps representing displacements caused by earthquakes or antenna exchanges occurring at times $t_i$; (3) a logarithmic decay — with characteristic decay time $\tau$ — representative of postseismic deformation due to fault afterslip induced by an earthquake occurred at time $t_{eq_j}$; (4) seasonal signals with annual ($T_1$) and semi-annual ($T_2$) periods. *H* is the unitary Heaviside step function and $\xi (t)$ represents formal uncertainties in the positional GNSS time series. Here, the parameters *A*, *v*, $B_i$, $C_j$, $D_k$ and $E_k$ are estimated by fitting the trajectory model to the observed time series using a linear weighted least squares method^61^. The decay parameter $\tau$ cannot be solved using the linear inversion, as the trajectory model (Eq. 1) has a nonlinear dependence on $\tau$. Therefore, we use a grid-search approach to find the optimal value of $\tau$ for each time series, where several solutions with different values of $\tau$ are evaluated. We then choose the value of $\tau$ that produces the lowest weighted root-mean-square (wrms) residual for each time series.

The trajectory model is fitted to each of the GNSS positional time series accounting for their formal uncertainties. However, it does not account for the Common Mode Error (CME), a spatially correlated error between different GNSS stations of a regional network. CME introduces a spatially coherent bias in the position of the GNSS stations due to uncertainties in the reference frame realizations, satellite orbits and clocks, as well as related to large-scale environmental effects^62^. To estimate the CME, we perform a stacking of the residual of the fitted trajectory models. We first use a mean motion filter to remove any low frequencies from the residuals, and compute the stacking after filtering. Finally, we remove the estimated CME from the data to recompute the different components of the trajectory model. The trajectory models for each of the series used are shown in Fig. S2.

### Earthquake catalog

In the present study, we extended the seismicity catalog of Sippl et al.^42^, which covers the time interval from 1 April 2014 to 31 December 2018 and contains 11,931 events for the north-central Chile region ($\sim$29.5$^{\circ }$–34.5$^{\circ }$S). Using data from 32 permanent seismic stations operated by the Centro Sismologico Nacional (CSN)^63^, we have extended this catalog to the end of 2021 using the same automated processing as described in Sippl et al.^42^. The newly obtained catalog includes 21,293 double-difference relocated earthquakes, the majority of which occurred at depths of $\le$60 km on or near the megathrust. We also searched for repeating earthquakes by station-wise cross-correlating event pairs using the criterion of Uchida and Matsuzawa^64^, which requires a cross-correlation coefficient of $\ge$0.95 at two or more stations (repeaters shown in Fig. 7). As the station network was extended in the first part of the covered time interval (years 2014 and 2015), the event catalog should be less complete for the first two years, so that event numbers before the Illapel earthquake as well as in the early part of the aftershock series are likely underestimated (Fig. 4b, c).

### Non-linear viscoelastic response using power law rheology

We use a finite element method (FEM) model to compute the nonlinear viscoelastic response due to the stress changes induced by the Illapel main shock (Fig. S4). The essential components of our mechanical model have been previously documented^45^, and here we describe the relevant aspects of our analysis. It is a forward geomechanical model considering power-law rheology with dislocation creep processes in the crust and upper mantle; it takes into account the slab geometry^65^ and the Moho discontinuity. The model domain is discretized into finite elements with a length of 4 km close to the region of coseismic slip, while we use a coarser element resolution at larger distances ($\sim$50 km length). As a result, the model domain is large enough to avoid boundary artifacts (Fig. S4). This model has already been extensively tested and used^19,45^.

We implement a temperature controlled power law rheology (Table S1) for the entire model domain described by the equation:

2 $$\begin{aligned} {\dot{\epsilon }} = A \sigma ^n exp (-Q/RT) \end{aligned}$$

where ${\dot{\epsilon }}$ is the strain rate, *A* is a pre-exponent parameter, $\sigma$ is the differential stress, *n* is the stress exponent, *Q* is the activation energy for creep, *R* is the gas constant, and *T* is the absolute temperature^66^. We use rock material properties that can explain the observed geodetic data in southern Chile^9,45,67^ and north-central Chile where the Illapel earthquake occurred. The values of the rheological properties are summarized in Table S1. The nonlinear viscoelastic parameters we used can also explain the first-order surface deformation recorded after the 2010 Maule event^67^. The resulting numerical problem is solved using the commercial FEM software ABAQUS^TM^, version 6.11. For each time window (T1–T4), we compute the nonlinear viscoelastic response due to the stress changes induced by the 2015 Illapel earthquake and subtract it from the observed geodetic measurements. We then use the residuals to estimate the afterslip distribution at each time window.

### Afterslip and locking degree distributions accross the megathurst fault

The fault slip is parameterized on a non-planar triangulated surface representing the contact between the Nazca and South American plates in the study region as defined by SLAB2^65^, ranging from the trench to a depth of 90 km.

The afterslip physical model is represented by Green’s functions (GFs) that are calculated assuming triangular dislocations in a homogeneous elastic half-space with Poisson’s ratio of 0.25 and using the methodology of Nikkhoo and Walter^68^. Here, we calculate the surface displacements due to a dislocation along the strike and dip directions at each triangular element of the fault. For the degree of locking, we use a viscoelastic FEM model to construct GFs, following the procedure and viscosity values for the continental and oceanic mantle used by Li et al.^47^ and the software Pylith^69^.

We use the least squares method with EPIC Tikhonov regularization^41^ to estimate afterslip and locking degree. The EPIC defines a spatially variable smoothing prior to compensate for the spatial variability of the observational constraints on fault slip. In this sense, it produces robust slip estimates that are less smoothed in the fault regions that are better constrained by the data, and more smoothed in regions that are less constrained by such observations. For this purpose, the following optimal problem is solved

3 $$\begin{aligned} \min _{{\textbf {m}}} || \mathbf {W_\chi }(\textbf{Gm}-{\textbf{d}})||^2_2 + ||\mathbf {W_h \nabla ^2m}||^2_2 \end{aligned}$$

where ${\textbf{d}}$ is the data vector (displacements or velocities), ${\textbf{G}}$ is the Green’s function, ${\textbf{m}}$ the model parameters to be estimated (afterslip or coupling degree), $\mathbf {W_\chi }$ the data misfit weight matrix, $\mathbf {W_h}$ is the matrix of regularization weights computed according to the EPIC, and $\mathbf {\nabla ^2}$ is a finite-difference approximation of the Laplacian operator applied to fault slip along the dip and strike directions. We impose positivity constraints on fault slip along the dip direction (dip slip $>= 0$). Using the L-curve method^46^, we map the trade-off between data misfit and regularization for each time window (Fig. S7) and determine the preferred model searching to balance both terms. We used the Monte Carlo propagation method to estimate the uncertainties of the optimal model.

The obtained afterslip estimates are constrained by the corrected accumulated 3D postseismic displacements measured at the GNSS stations in each time window. The displacements are corrected by subtracting the prediction of the modeled viscoelastic response caused by the mainshock slip of either Carrasco et al.^32^ or Tilmann et al.^27^ (Fig. S5). We also compare these results with inversions using postseismic displacements without viscoelastic corrections (Figs. S6,  S8,  S9). The estimated locking degree (Fig. 6) is constrained by interseismic rates. To obtain such rates, we subtract the postseismic component from each of the GNSS time series and use the trajectory model to estimate the linear trend for the period from 2018 to 2021 (i.e., 4 years of observation). We chose this period for the locking analysis because most of the postseismic deformation has drastically decreased.

### Clustering analysis

Clustering is an unsupervised machine learning method used to autonomously evaluate the data distribution in feature space. We use the agglomerative clustering algorithm implemented in sklearn-scikit^48^. This is a hierarchical clustering with a bottom-up approach. The algorithm first treats each object as a single cluster. Then, the pairs of clusters are successively merged until all clusters are merged into one large cluster containing all objects. We use the Ward linkage criterion, which merges clusters that cause the least increase in intra-cluster variance. We use a homogeneous grid to extract the values of coseismic slip, afterslip, locking, and seismic moment of $M_w<7$ events and use these four datasets as features in the cluster analysis. We fit Gaussian Mixture models applying the BIC to determine the optimal number of clusters. We assume that the data points come from multi-dimensional Gaussian distributions, so the lower the BIC values, the better the model.

### Supplementary Information

Supplementary Information.

## Data Availability

The daily GNSS time series analyzed in the current study are available in the Nevada Geodetic Laboratory (NGL)^58^ repository (http://geodesy.unr.edu/NGLStationPages/gpsnetmap/GPSNetMap.html). All GNSS time series used in this study can be found in the Supplementary Information.

## Codes availability

The python codes for least squares inversion with EPIC Tikhonov regularization are available at https://github.com/frortega/EPIC_LS^41^. 
