# Supplementary material for: Fast relocking and afterslip-seismicity evolution following the 2015 Mw 8.3 Illapel earthquake in Chile
Source: Sci Rep. 2023 Nov 9;13:19511. doi: 10.1038/s41598-023-45369-9 (PMC10636185; doi:10.1038/s41598-023-45369-9)
Supplement: Supplementary file 1 — Supplementary Information. [file 41598_2023_45369_MOESM1_ESM.pdf]

Fast relocking and afterslip-seismicity evolution following the 2015 Mw 8.3 Illapel earthquake in Chile

Joaquín Hormazábal[1+], Marcos Moreno[2,+,*], Francisco Ortega-Culaciati[1,+], Juan Carlos Báez[3], Carlos Peña[4], Christian Sippl[5], Diego González-Vidal[6], Javier Ruiz[1], Sabrina Metzger[4], and Shoichi Yoshioka[7,8]

[1]Departamento de Geofísica, Facultad de Ciencias Físicas y Matemáticas, Universidad de Chile, Santiago, Chile.
[2]Departamento de Ingeniería Estructural y Geotécnica, Pontificia Universidad Católica, Santiago, Chile.
[3]Centro Sismológico Nacional, Facultad de Ciencias Físicas y Matemáticas, Universidad de Chile, Santiago, Chile.
[4]Helmholtz Centre Potsdam, GFZ German Research Centre for Geosciences, Potsdam, Germany
[5]Institute of Geophysics of the Czech Academy of Sciences, Prague, Czech Republic
[6]Department of Earth Science, University of Concepción, Concepción, Chile.
[7]Research Center for Urban Safety and Security, Kobe University, Rokkodai-cho 1-1, Nada Ward, Kobe, 657-8501, Japan.
[8]Department of Planetology, Graduate School of Science, Kobe University, Rokkodai-cho 1-1, Nada Ward, Kobe 657-8501, Japan.
[*]marcos.moreno@uc.cl
[+]these authors contributed equally to this work

Content

This supporting information provides supporting figures, and tables.

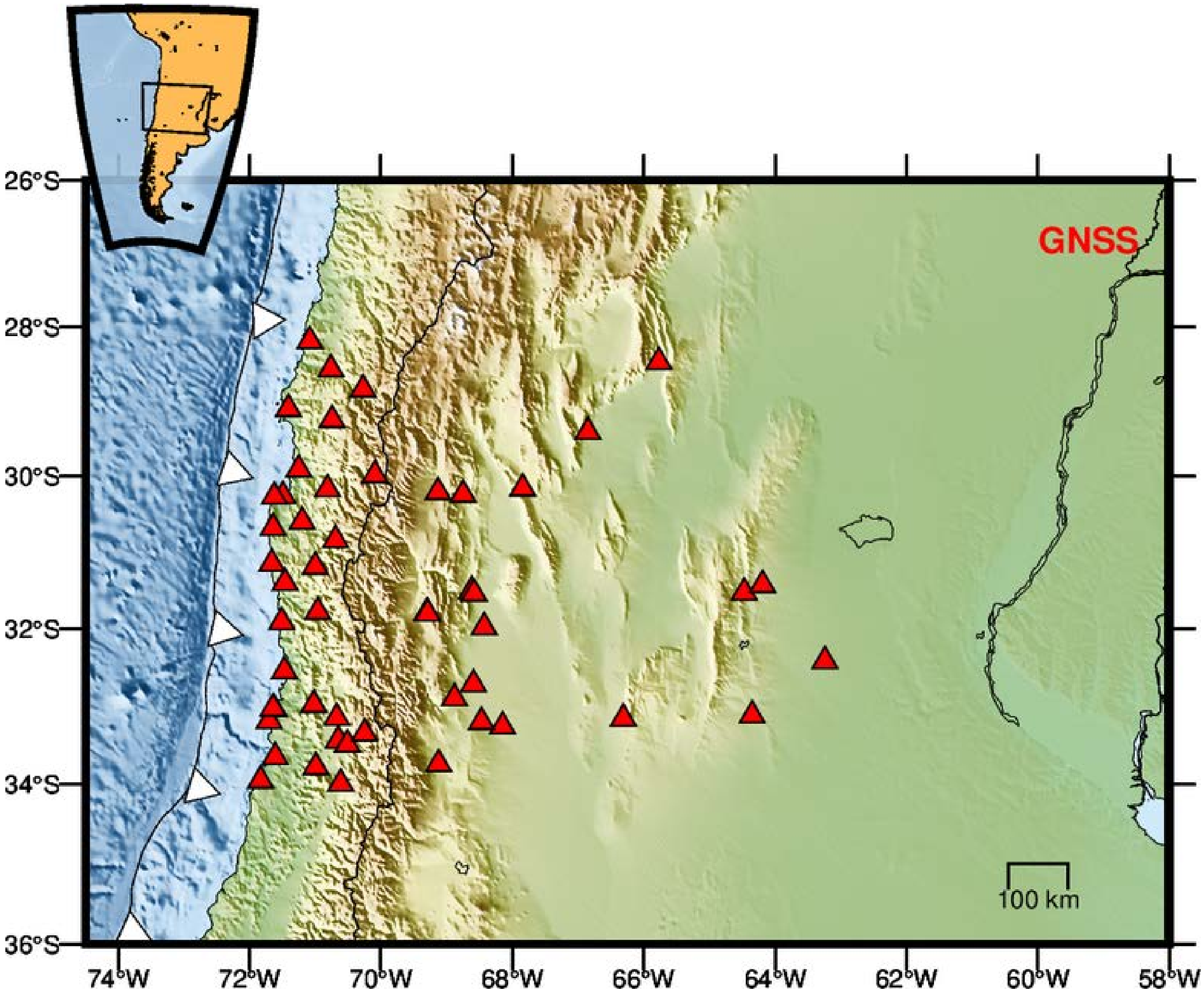

Figure S1. Distribution of GNSS continuous stations used in this work. All time series used in this analysis are shown in Fig. S2. The figure was created using GMT 6 (Generic Mapping Tools) software[1].

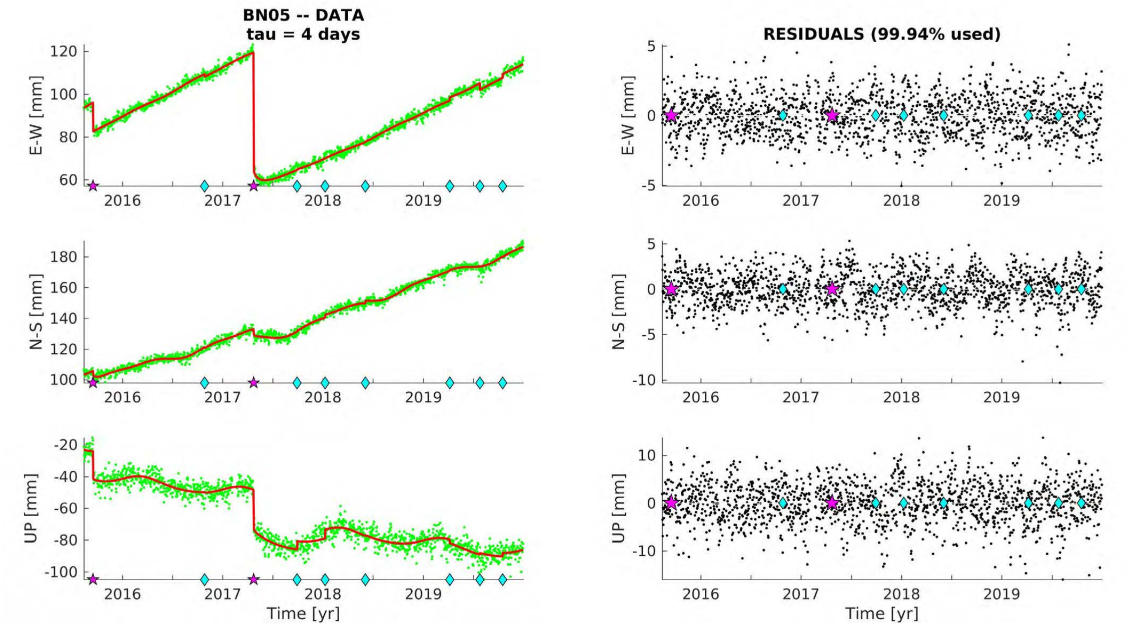

Figure S2. Series of figures showing the GNSS time-series (left column) for east, north and vertical component and the model residuals (right column). The red lines represent the predictions of the trajectory model. The red lines and the green dots represent the predictions of the trajectory model and the daily observations, respectively. The model accounts for a linear, interseismic rate, antenna offsets (Cyan diamond symbol), earthquake offsets (fuchsia stars), postseismic decays, and seasonal oscillations. All time series used in this analysis are included at the end of this document.

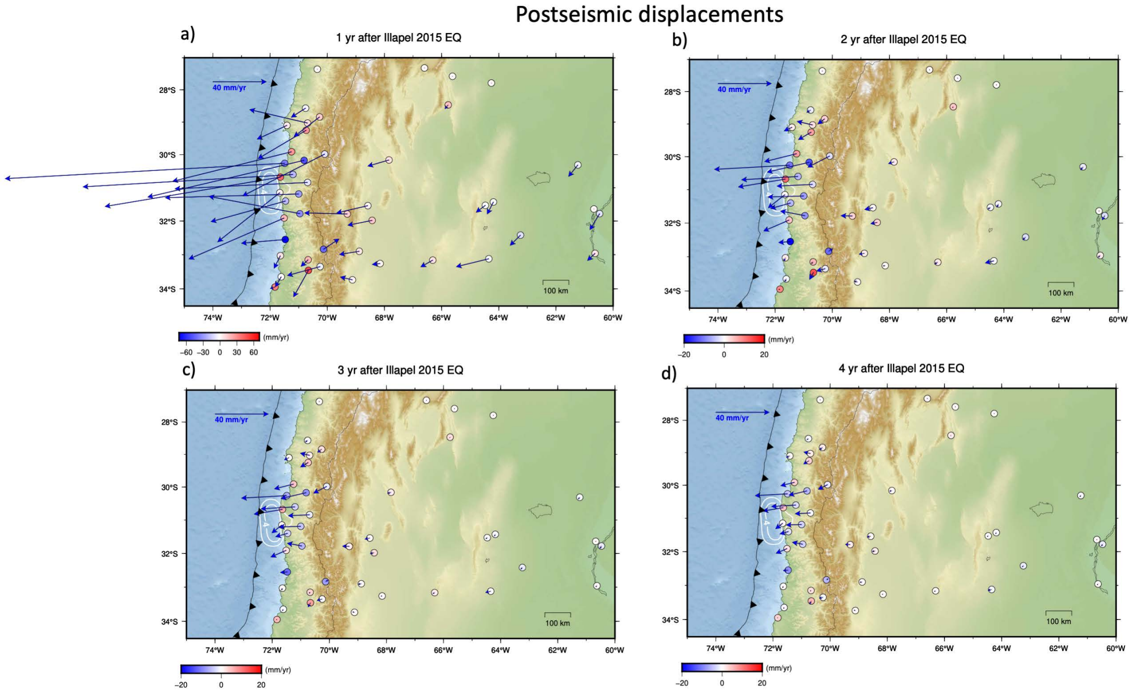

Figure S3. Logarithmic decay components from the trajectory models for one (a), two (b), three (c), and four (d) years after the Illapel earthquake. Colored circles show vertical displacements. The figure was created using GMT 6 (Generic Mapping Tools) software[1].

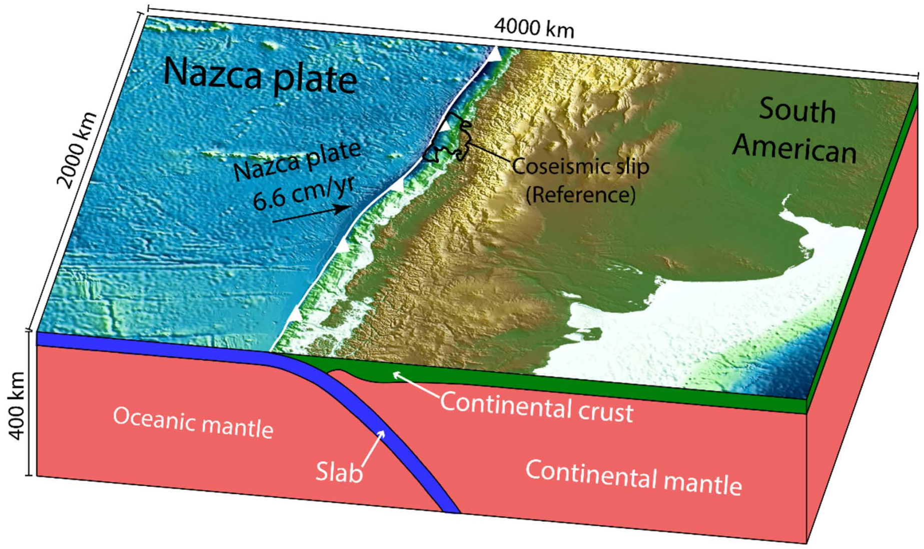

Figure S4. FEM model configuration. The geometry and rheological blocks of the numerical model used to estimate the postseismic viscoelastic relaxation are shown. The figure was created using GMT 6 (Generic Mapping Tools) software[1].

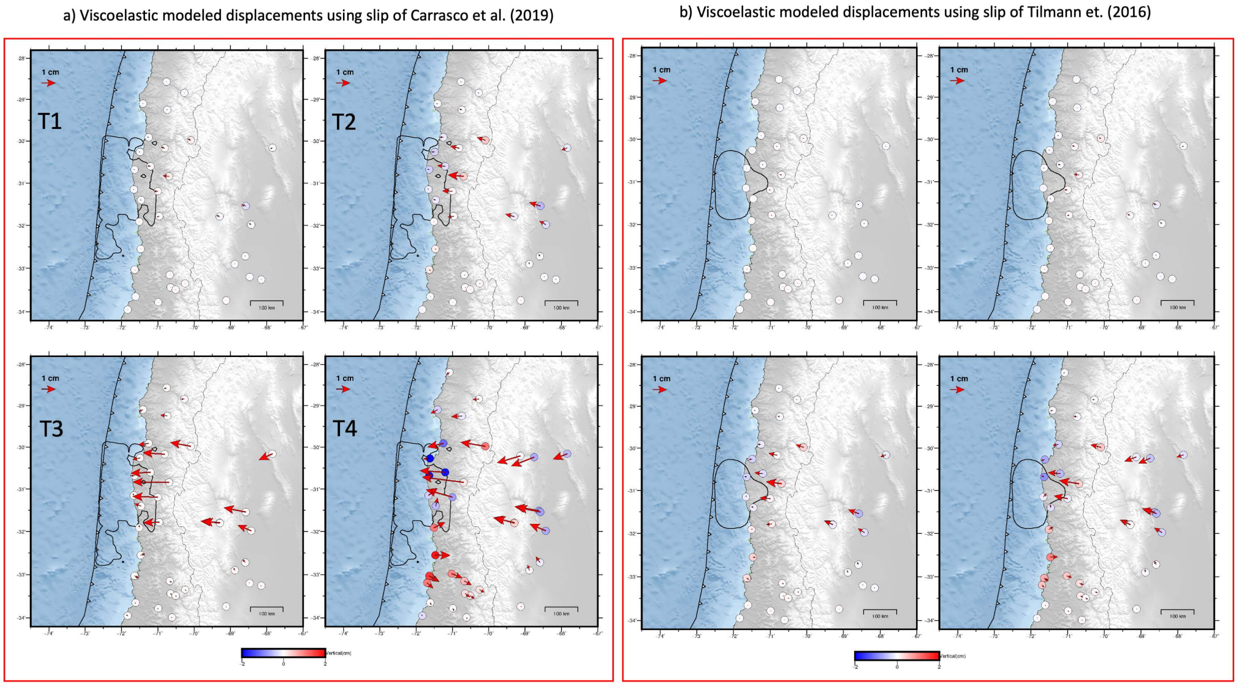

Figure S5. Predictions of the viscoelastic model using the coseismic slip of Carrasco et al. (2019)[2] (left panel) and Tilmann et al. (2016)[3] (right panel) as inputs. Colored circles show vertical displacements. The figure was created using GMT 6 (Generic Mapping Tools) software[1].

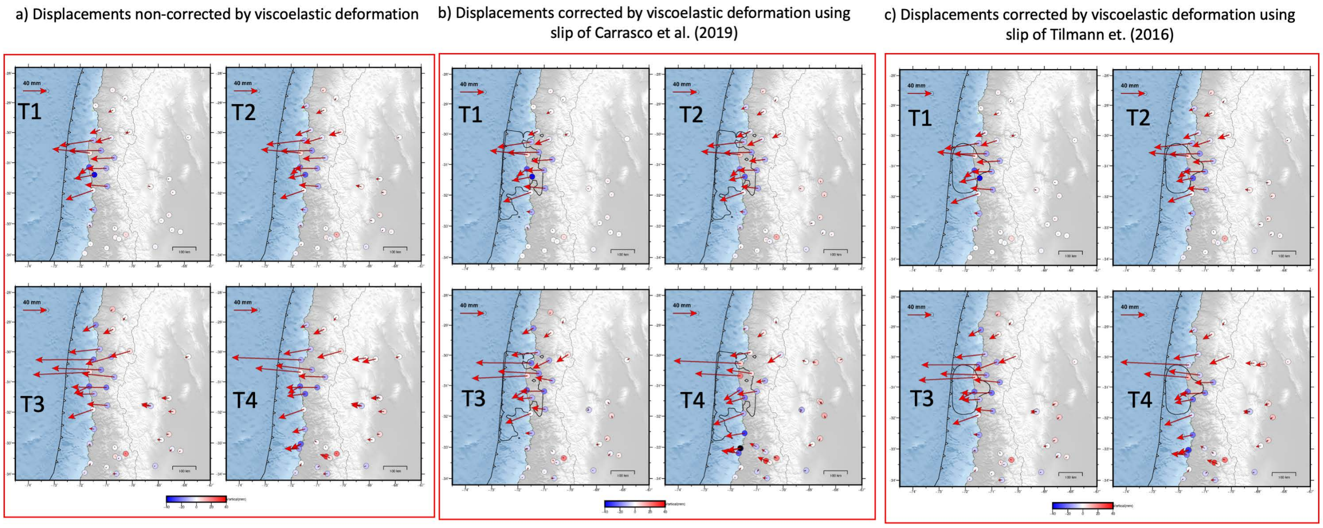

Figure S6. Cumulative postseismic displacements for each time window from data without viscoelastic corrections (a), and displacements corrected by the viscoelastic signal using slip from Carrasco et al. (2019)[2] (b) and Tilmann et al. (2016)[3] as inputs in the FEM-model(c). The figure was created using GMT 6 (Generic Mapping Tools) software[1].

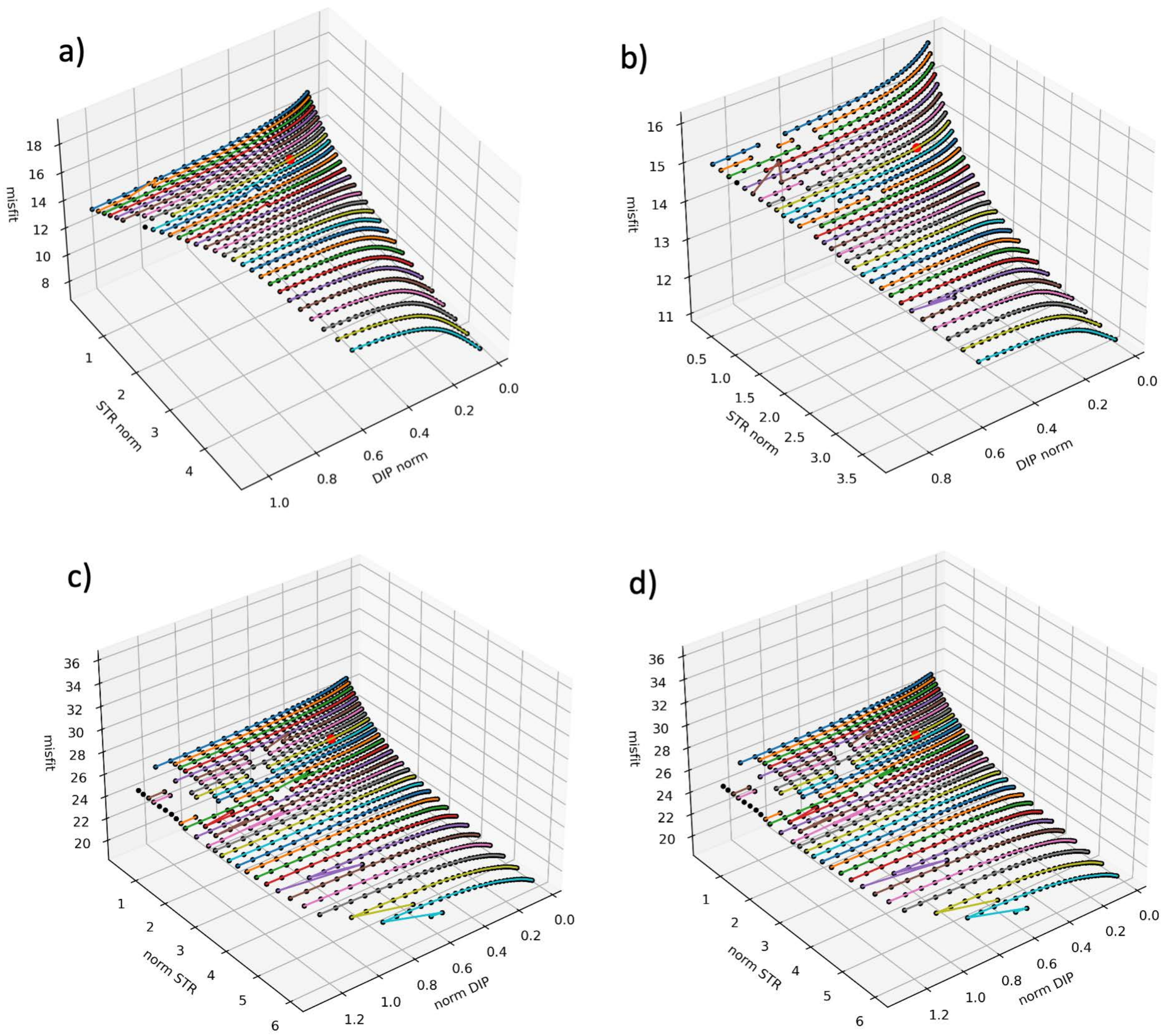

Figure S7. L-curve plots to determine the optimal afterslip models for time windows T1 (a), T2 (b), T3 (c), and T4 (d).

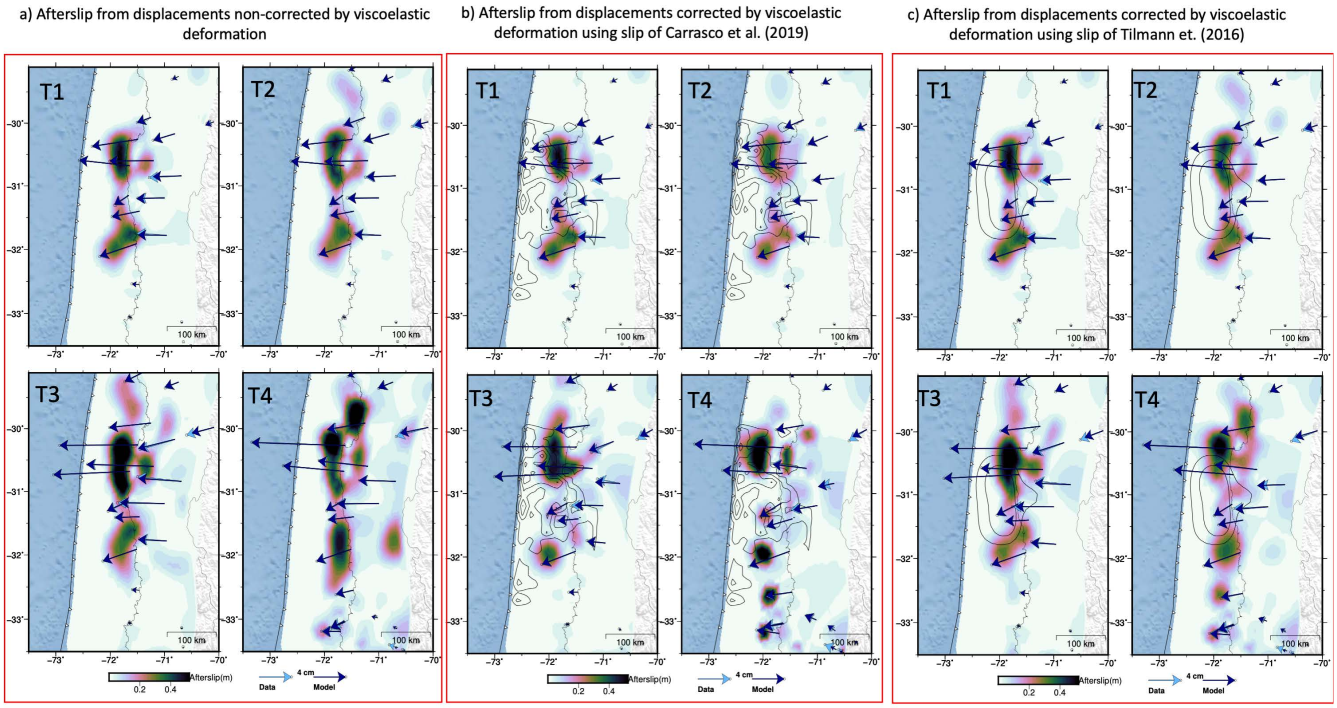

Figure S8. Afterslip distributions for each time window using uncorrected displacements (a) and displacements with viscoelastic corrections modeled from the coseismic slip of Carrasco et al. (2019)[2] (b) and Tilmann et al. (2016)[3] (c). The figure was created using GMT 6 (Generic Mapping Tools) software[1].

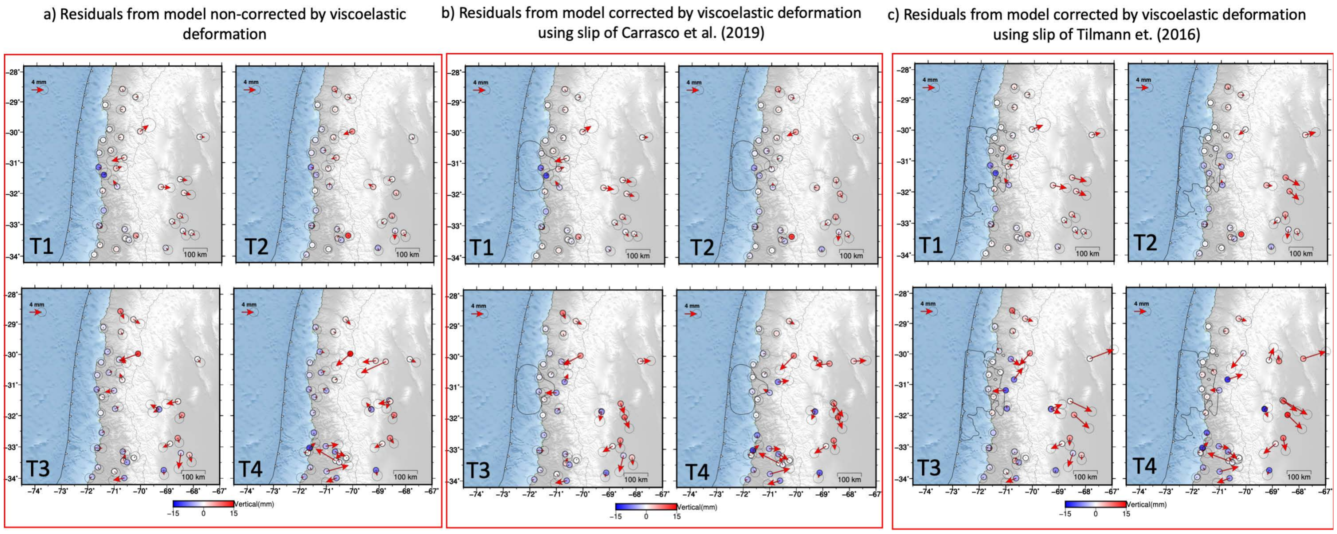

Figure S9. Residuals between the measurements and afterslip model predictions using uncorrected displacements (a) and displacements with viscoelastic corrections modeled from the coseismic slip of Carrasco et al. (2019)[2] (b) and Tilmann et al. (2016)[3] (c). The figure was created using GMT 6 (Generic Mapping Tools) software[1].

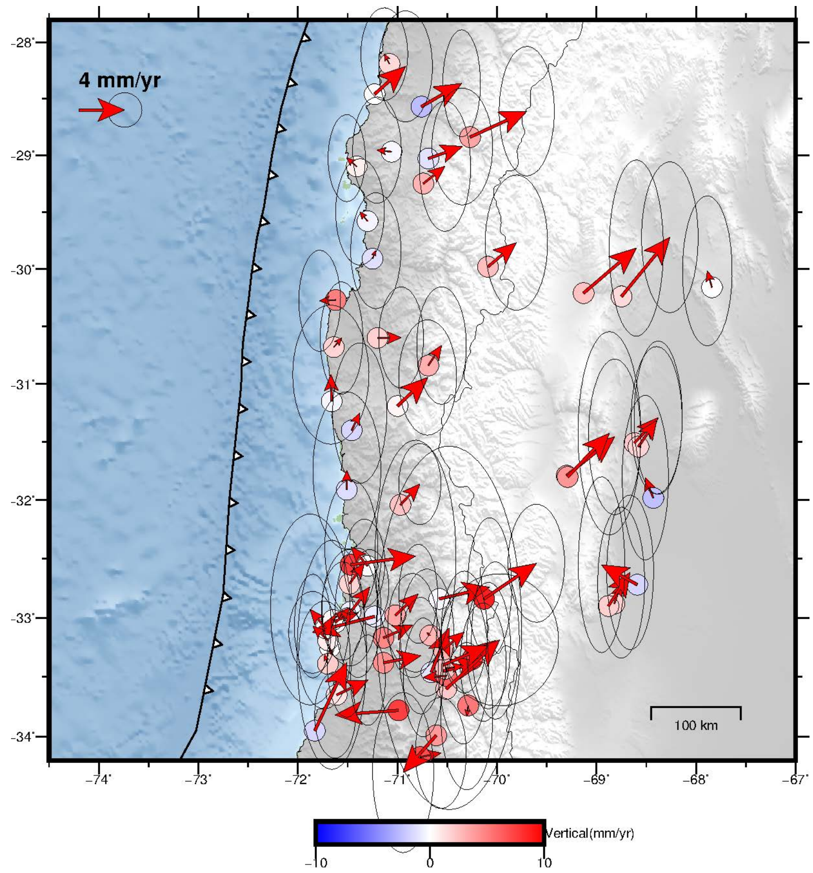

Figure S10. Residuals between the measurements and locking model predictions. The figure was created using GMT 6 (Generic Mapping Tools) software[1].

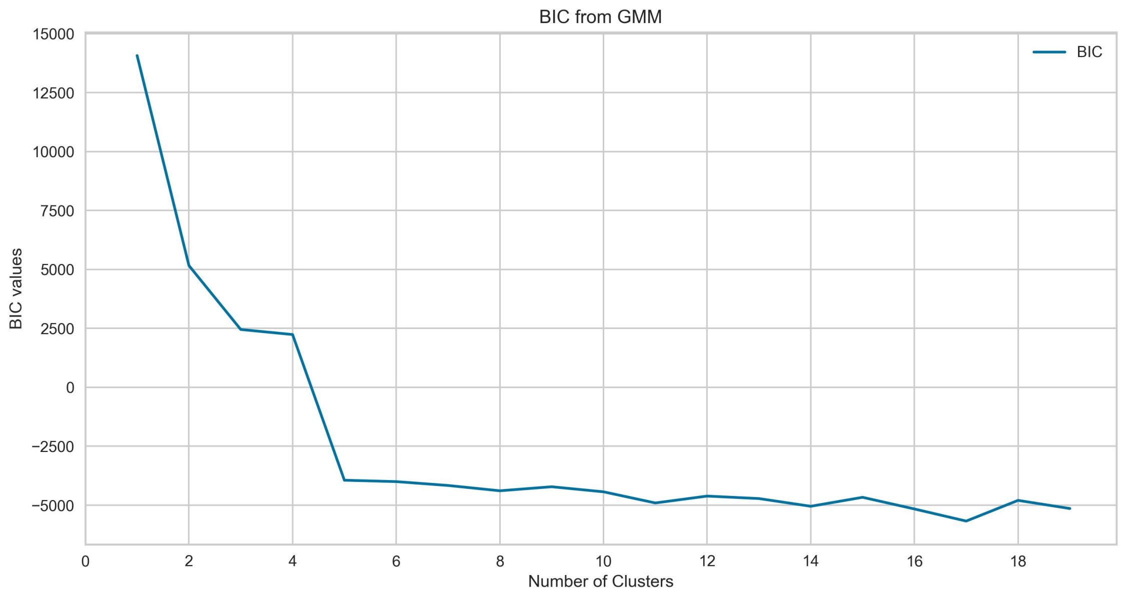

Figure S11. Plot between number of clusters and Bayesian Information Criterion (BIC) values. We chose an optimal number of four clusters, which gives a local minimum BIC.

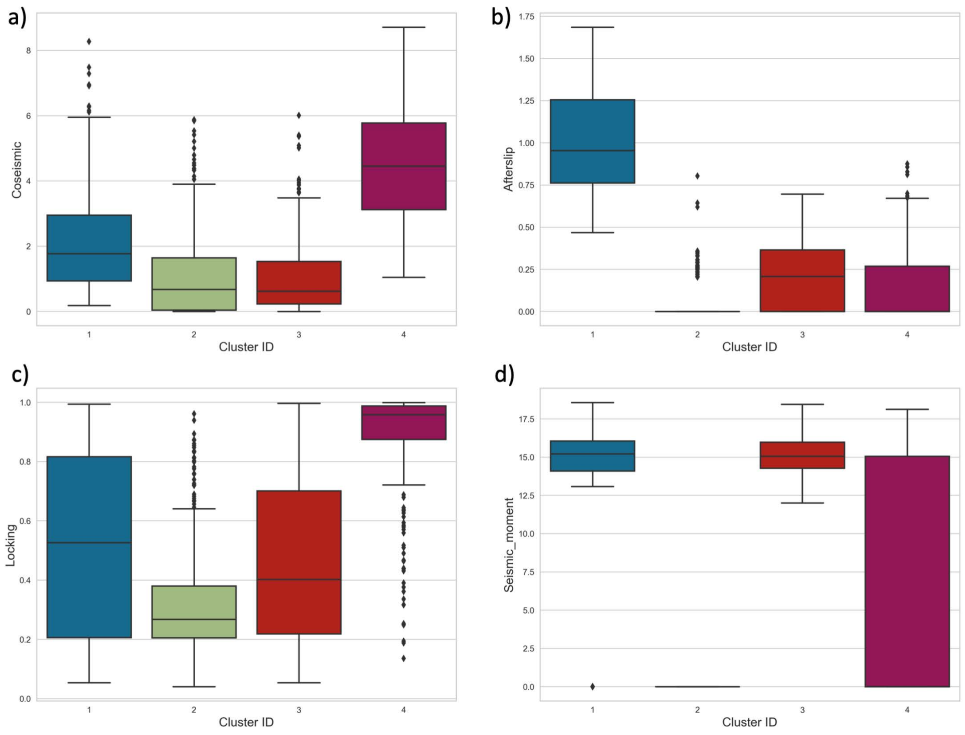

Figure S12. Box plots of the cluster analysis showing the distribution of the cluster in each feature: a) coseismic slip, b) afterslip, c) locking degree, and d) seismic moment estimate.

Model domain	Rock type[b]	Young's modulus E [GPa]	Poisson's ratio v[a]	Pre-exponent A [$Mpa^{-n}s^{-1}$]	Stress exponent n	Activation energy Q [kJ mol^{-1}]
Continental crust	Wet quartzite	100	0.265	3.2×10^{-4}	2.3	154
Continental upper mantle	Wet olivine 1	160	0.25	5.6×10^{6}	3.5	480
Oceanic upper mantle	Wet olivine 2	160	0.25	1.6×10^{5}	3.5	480
Slab	Diabase	120	0.3	2.0×10^{-4}	3.4	260

Table S 1. Elastic properties and dislocation creep parameters used in the FEM modeling.

References

1. Wessel, P. *et al.* The generic mapping tools version 6. *Geochem. Geophys. Geosystems* **20**, 5556–5564, DOI: 10.1029/2019GC008515 (2019).
2. Carrasco, S., Ruiz, J. A., Contreras-Reyes, E. & Ortega-Culaciati, F. Shallow intraplate seismicity related to the Illapel 2015 Mw 8.4 earthquake: Implications from the seismic source. *Tectonophysics* **766**, 205–218, DOI: 10.1016/j.tecto.2019.06.011 (2019).
3. Tilmann, F. *et al.* The 2015 Illapel earthquake, central Chile: A type case for a characteristic earthquake? *Geophys. Res. Lett.* **43**, 574–583, DOI: 10.1002/2015GL066963 (2016).

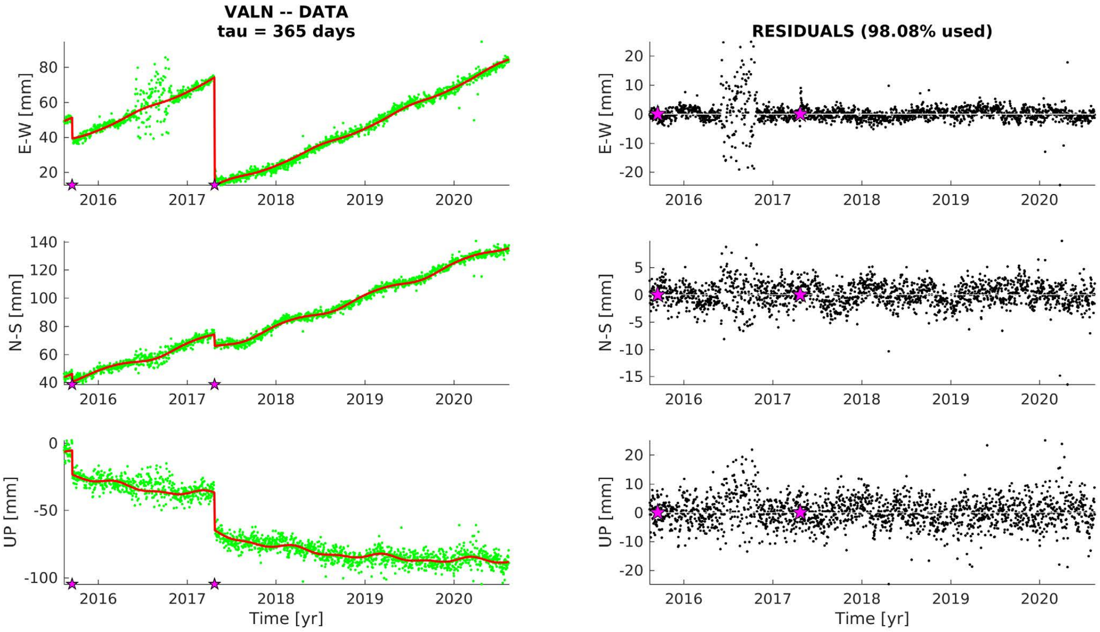

Figure S13. Series of figures showing the GNSS time-series (left column) for east, north and vertical component and the model residuals (right column). The red lines represent the predictions of the trajectory model. The red lines and the green dots represent the predictions of the trajectory model and the daily observations, respectively. The model accounts for a linear, interseismic rate, antenna offsets (Cyan diamond symbol), earthquake offsets (fuchsia stars), postseismic decays, and seasonal oscillations.

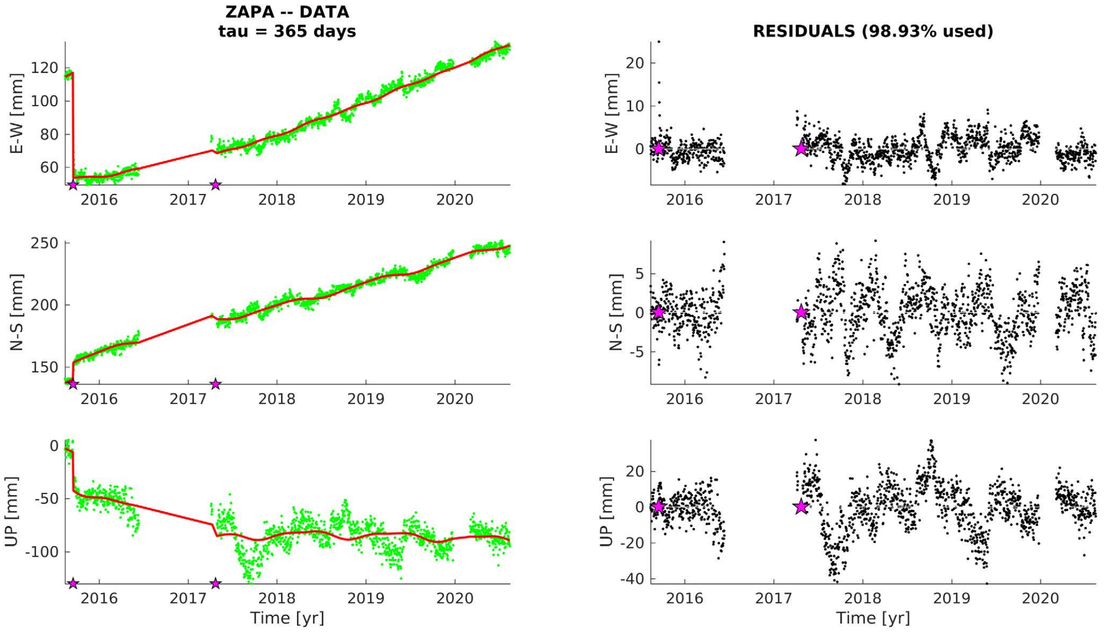

Figure S14. Series of figures showing the GNSS time-series (left column) for east, north and vertical component and the model residuals (right column). The red lines represent the predictions of the trajectory model. The red lines and the green dots represent the predictions of the trajectory model and the daily observations, respectively. The model accounts for a linear, interseismic rate, antenna offsets (Cyan diamond symbol), earthquake offsets (fuchsia stars), postseismic decays, and seasonal oscillations.

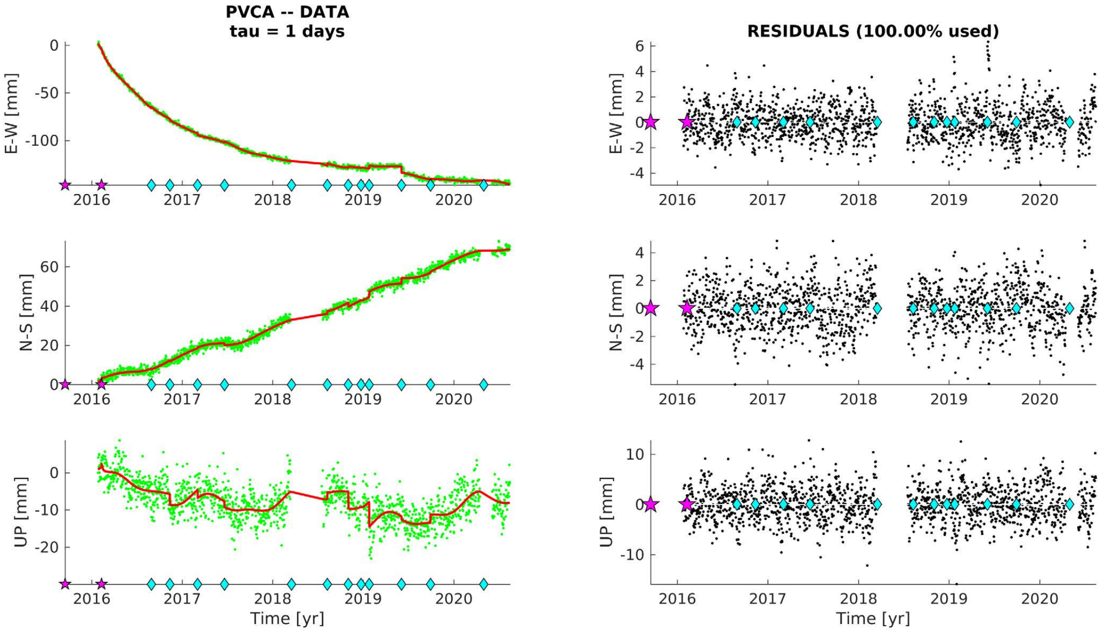

Figure S15. Series of figures showing the GNSS time-series (left column) for east, north and vertical component and the model residuals (right column). The red lines represent the predictions of the trajectory model. The red lines and the green dots represent the predictions of the trajectory model and the daily observations, respectively. The model accounts for a linear, interseismic rate, antenna offsets (Cyan diamond symbol), earthquake offsets (fuchsia stars), postseismic decays, and seasonal oscillations.

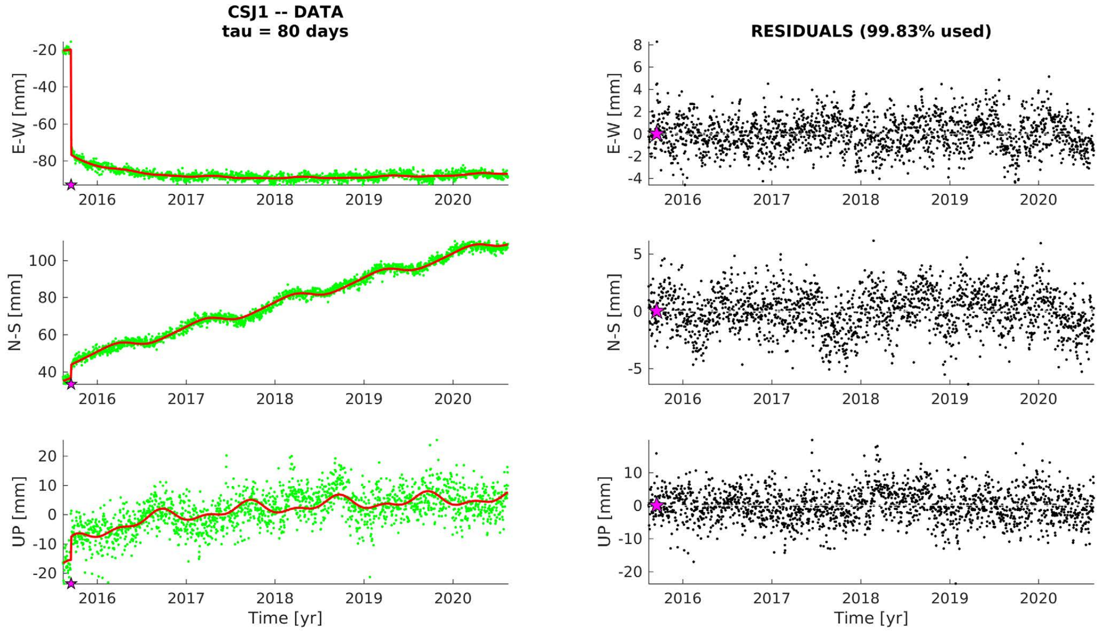

Figure S16. Series of figures showing the GNSS time-series (left column) for east, north and vertical component and the model residuals (right column). The red lines represent the predictions of the trajectory model. The red lines and the green dots represent the predictions of the trajectory model and the daily observations, respectively. The model accounts for a linear, interseismic rate, antenna offsets (Cyan diamond symbol), earthquake offsets (fuchsia stars), postseismic decays, and seasonal oscillations.

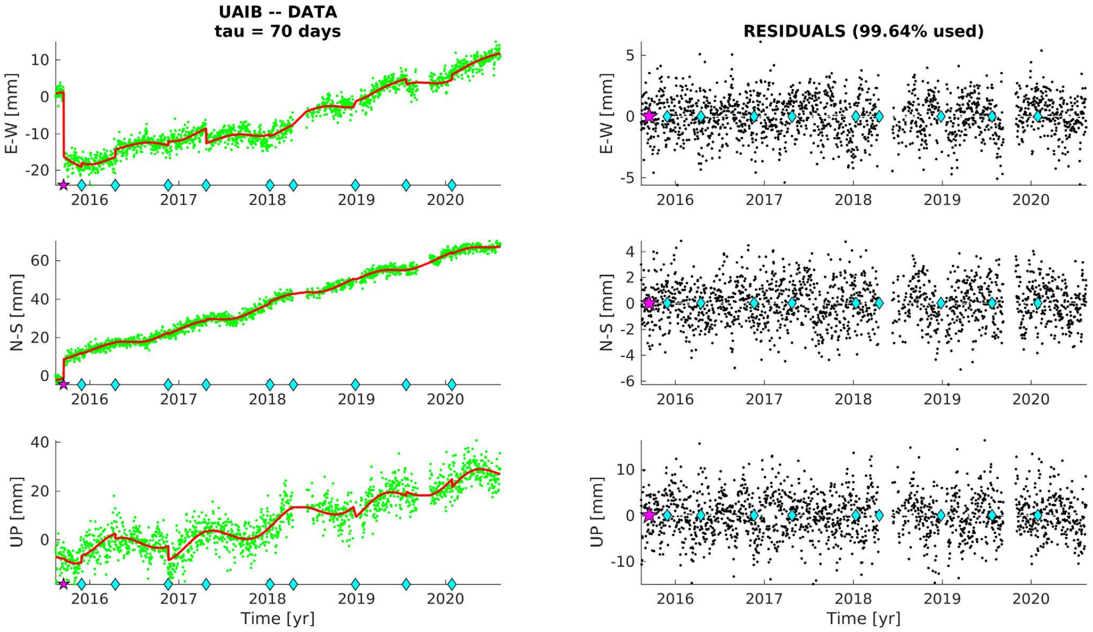

Figure S17. Series of figures showing the GNSS time-series (left column) for east, north and vertical component and the model residuals (right column). The red lines represent the predictions of the trajectory model. The red lines and the green dots represent the predictions of the trajectory model and the daily observations, respectively. The model accounts for a linear, interseismic rate, antenna offsets (Cyan diamond symbol), earthquake offsets (fuchsia stars), postseismic decays, and seasonal oscillations.

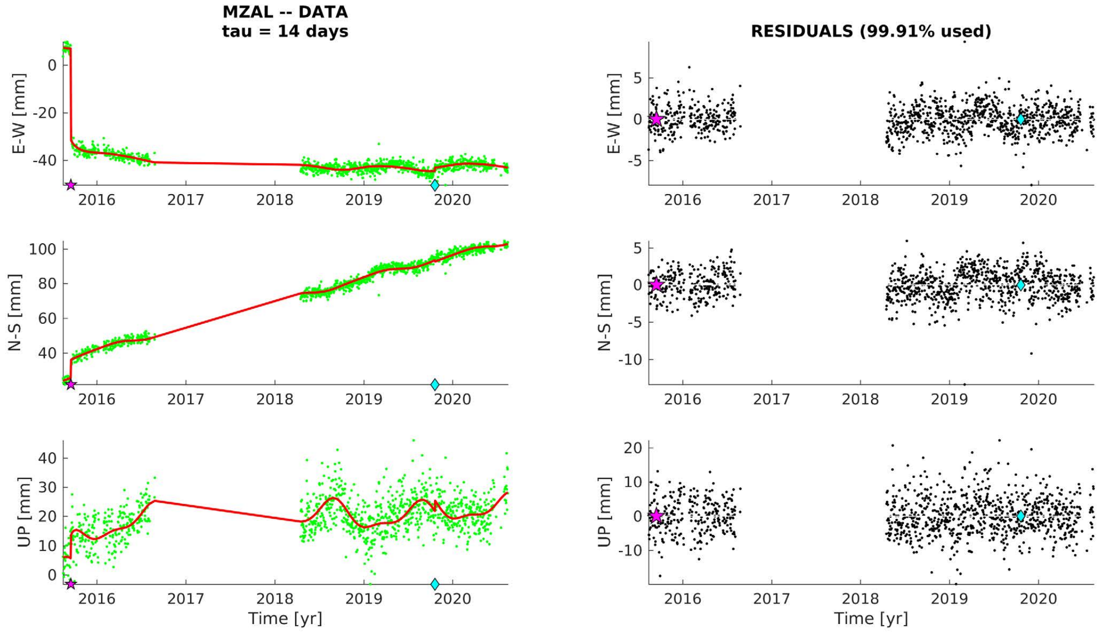

Figure S18. Series of figures showing the GNSS time-series (left column) for east, north and vertical component and the model residuals (right column). The red lines represent the predictions of the trajectory model. The red lines and the green dots represent the predictions of the trajectory model and the daily observations, respectively. The model accounts for a linear, interseismic rate, antenna offsets (Cyan diamond symbol), earthquake offsets (fuchsia stars), postseismic decays, and seasonal oscillations.

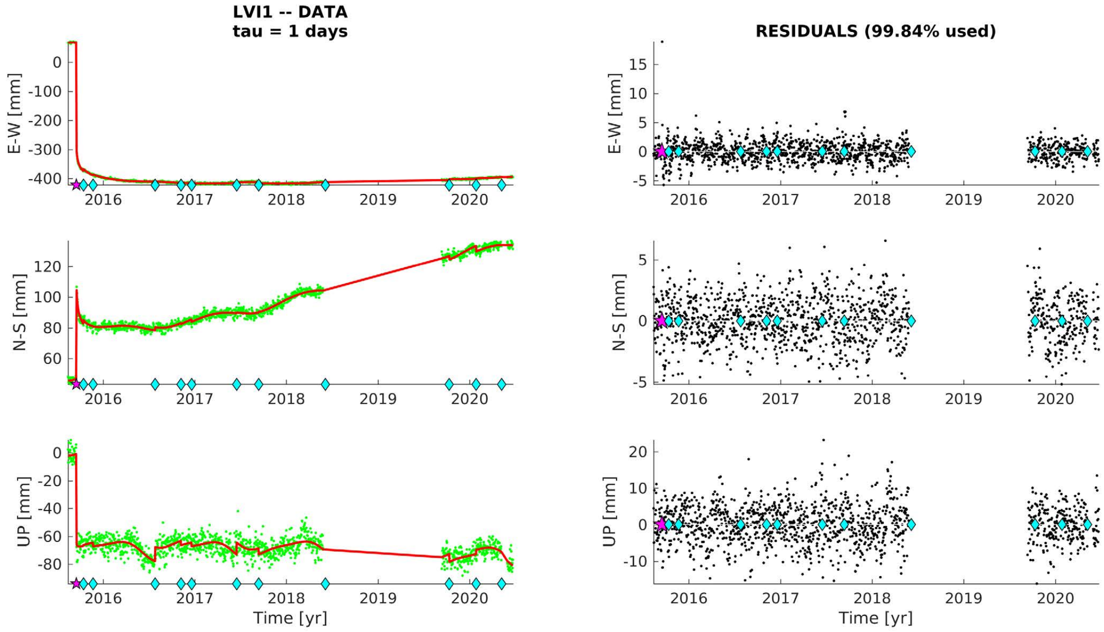

Figure S19. Series of figures showing the GNSS time-series (left column) for east, north and vertical component and the model residuals (right column). The red lines represent the predictions of the trajectory model. The red lines and the green dots represent the predictions of the trajectory model and the daily observations, respectively. The model accounts for a linear, interseismic rate, antenna offsets (Cyan diamond symbol), earthquake offsets (fuchsia stars), postseismic decays, and seasonal oscillations.

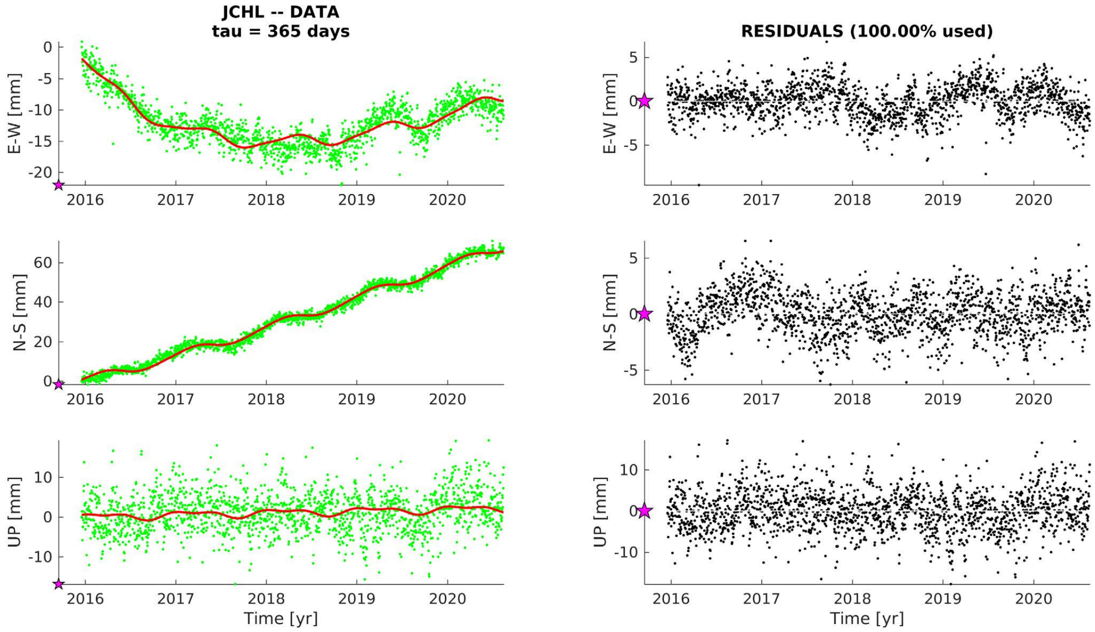

Figure S20. Series of figures showing the GNSS time-series (left column) for east, north and vertical component and the model residuals (right column). The red lines represent the predictions of the trajectory model. The red lines and the green dots represent the predictions of the trajectory model and the daily observations, respectively. The model accounts for a linear, interseismic rate, antenna offsets (Cyan diamond symbol), earthquake offsets (fuchsia stars), postseismic decays, and seasonal oscillations.

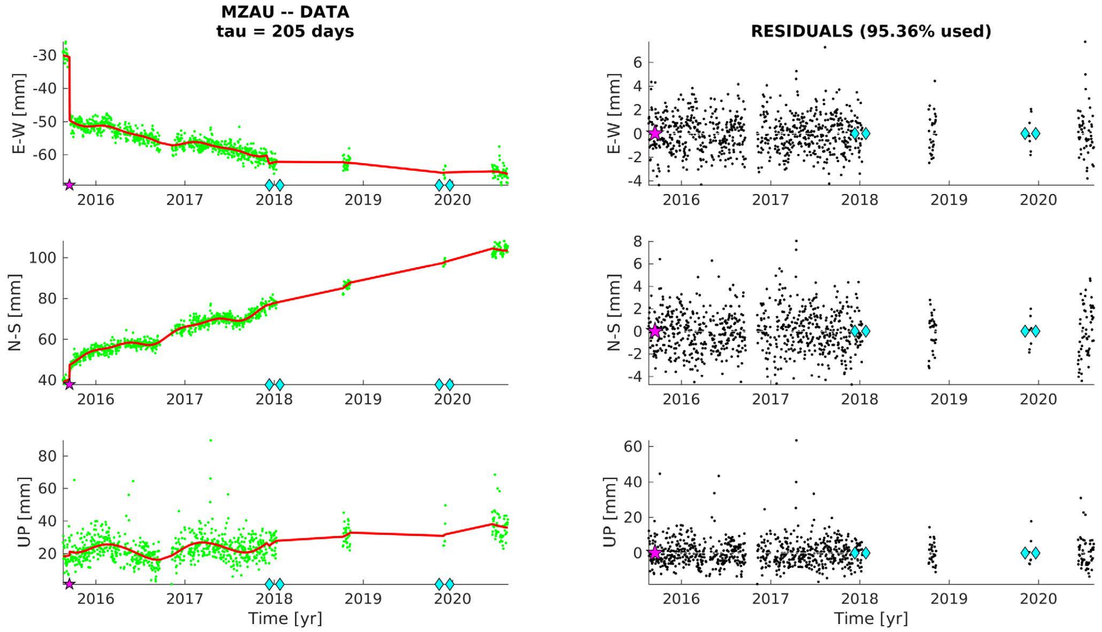

Figure S21. Series of figures showing the GNSS time-series (left column) for east, north and vertical component and the model residuals (right column). The red lines represent the predictions of the trajectory model. The red lines and the green dots represent the predictions of the trajectory model and the daily observations, respectively. The model accounts for a linear, interseismic rate, antenna offsets (Cyan diamond symbol), earthquake offsets (fuchsia stars), postseismic decays, and seasonal oscillations.

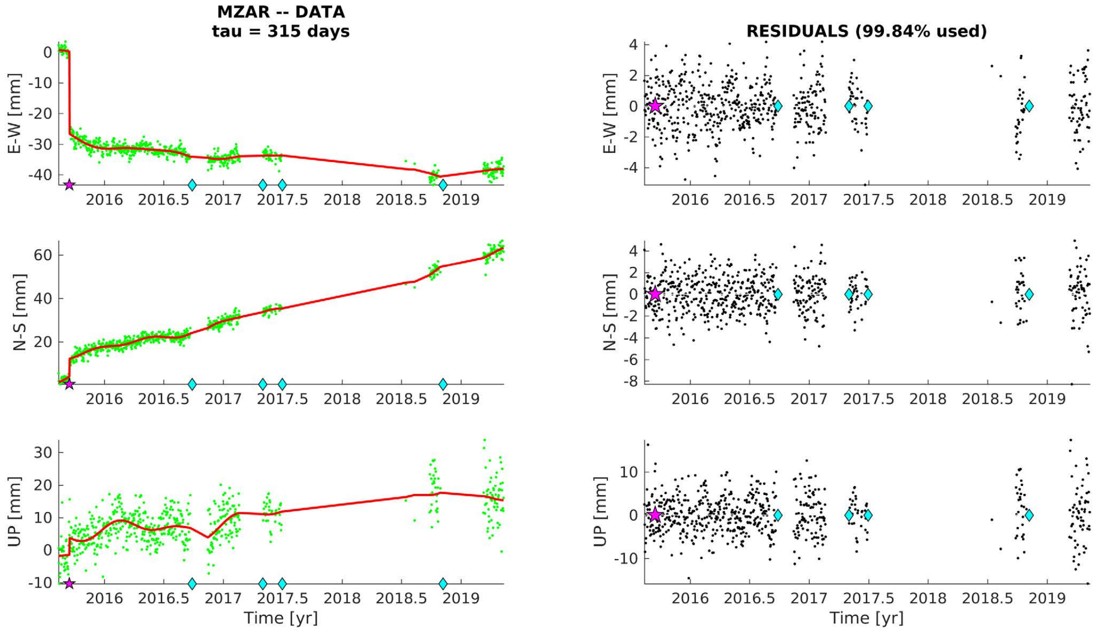

Figure S22. Series of figures showing the GNSS time-series (left column) for east, north and vertical component and the model residuals (right column). The red lines represent the predictions of the trajectory model. The red lines and the green dots represent the predictions of the trajectory model and the daily observations, respectively. The model accounts for a linear, interseismic rate, antenna offsets (Cyan diamond symbol), earthquake offsets (fuchsia stars), postseismic decays, and seasonal oscillations.

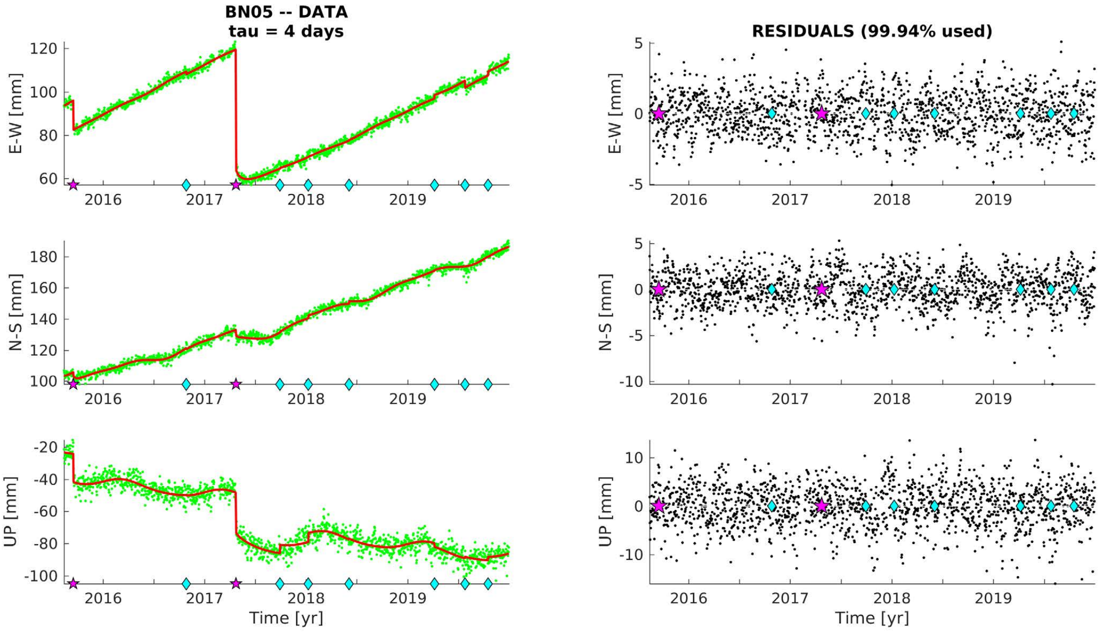

Figure S23. Series of figures showing the GNSS time-series (left column) for east, north and vertical component and the model residuals (right column). The red lines represent the predictions of the trajectory model. The red lines and the green dots represent the predictions of the trajectory model and the daily observations, respectively. The model accounts for a linear, interseismic rate, antenna offsets (Cyan diamond symbol), earthquake offsets (fuchsia stars), postseismic decays, and seasonal oscillations.

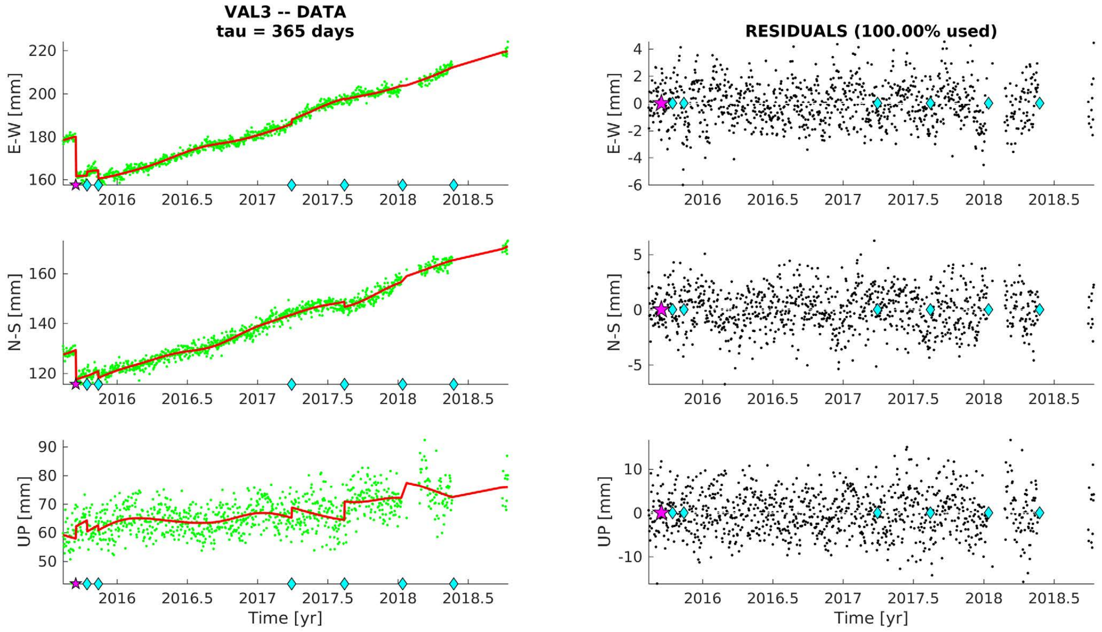

Figure S24. Series of figures showing the GNSS time-series (left column) for east, north and vertical component and the model residuals (right column). The red lines represent the predictions of the trajectory model. The red lines and the green dots represent the predictions of the trajectory model and the daily observations, respectively. The model accounts for a linear, interseismic rate, antenna offsets (Cyan diamond symbol), earthquake offsets (fuchsia stars), postseismic decays, and seasonal oscillations.

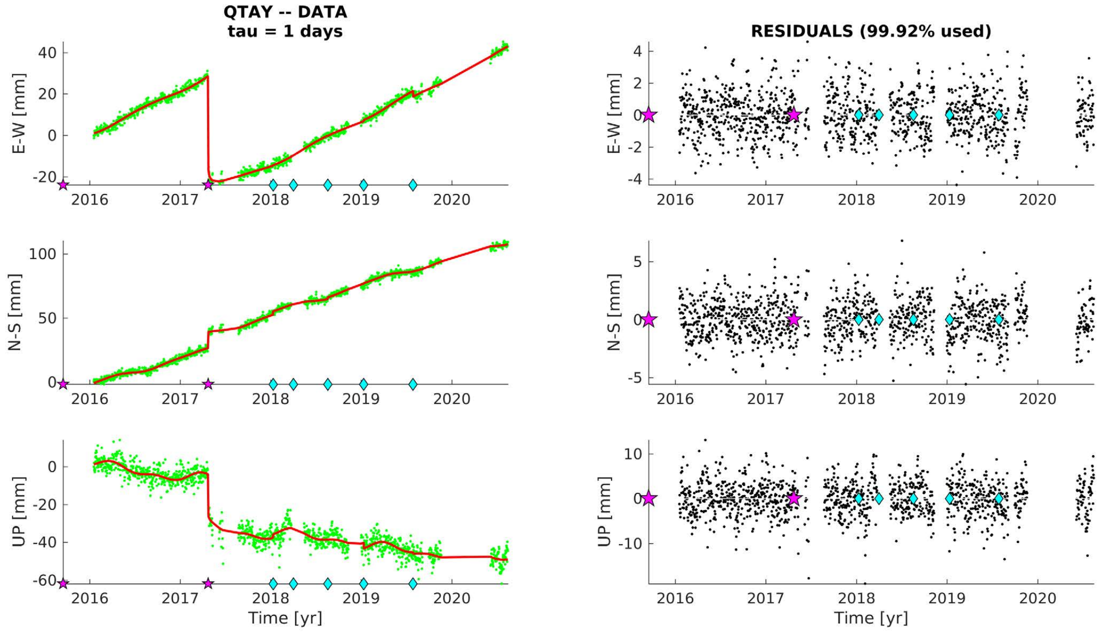

Figure S25. Series of figures showing the GNSS time-series (left column) for east, north and vertical component and the model residuals (right column). The red lines represent the predictions of the trajectory model. The red lines and the green dots represent the predictions of the trajectory model and the daily observations, respectively. The model accounts for a linear, interseismic rate, antenna offsets (Cyan diamond symbol), earthquake offsets (fuchsia stars), postseismic decays, and seasonal oscillations.

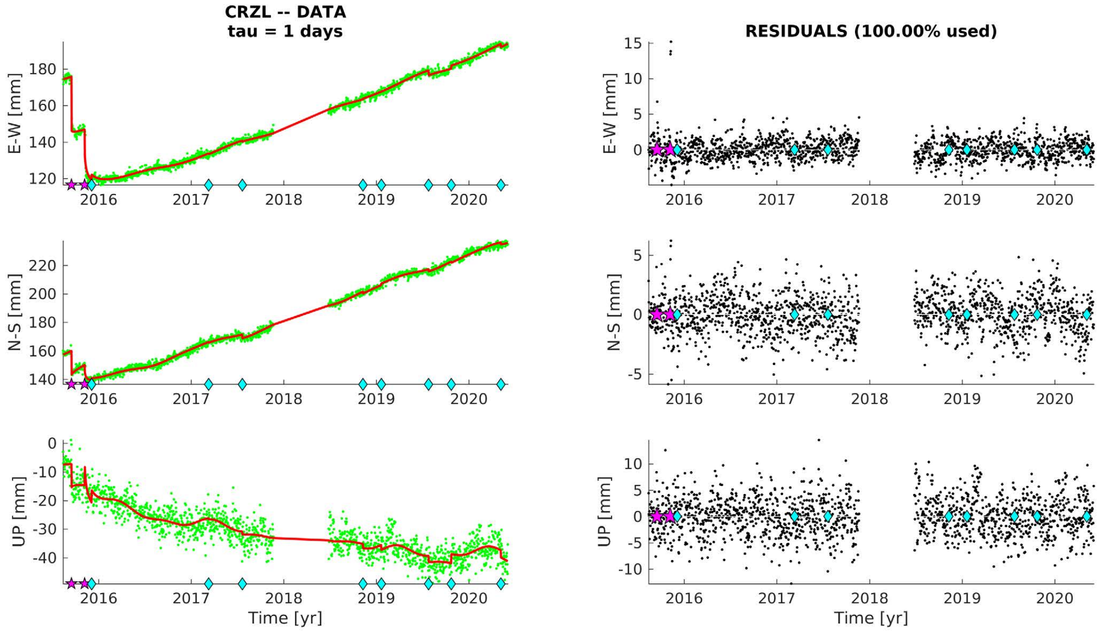

Figure S26. Series of figures showing the GNSS time-series (left column) for east, north and vertical component and the model residuals (right column). The red lines represent the predictions of the trajectory model. The red lines and the green dots represent the predictions of the trajectory model and the daily observations, respectively. The model accounts for a linear, interseismic rate, antenna offsets (Cyan diamond symbol), earthquake offsets (fuchsia stars), postseismic decays, and seasonal oscillations.

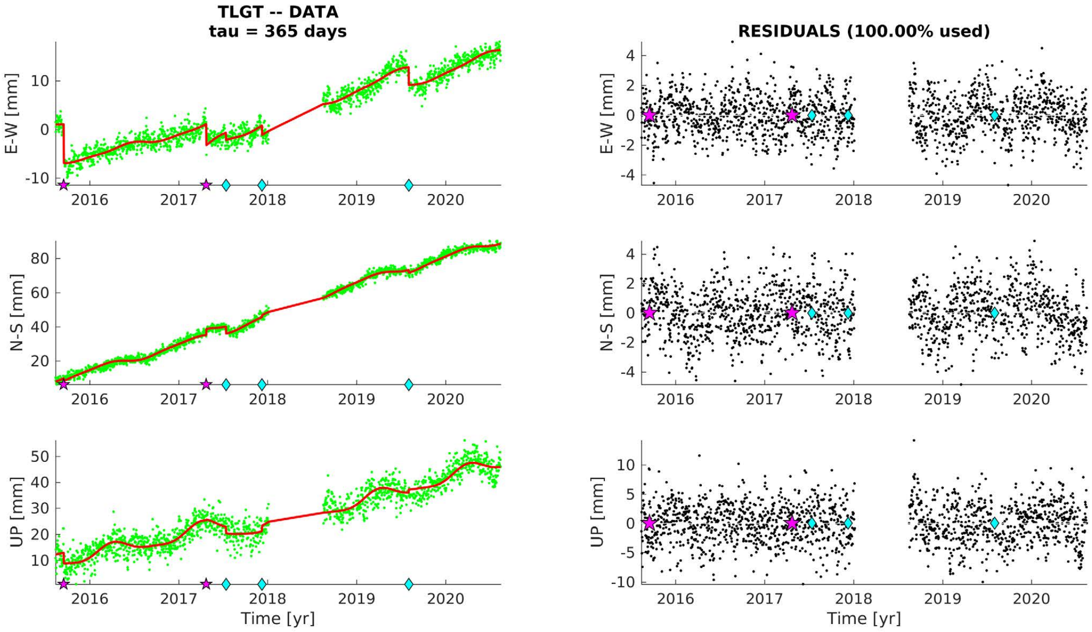

Figure S27. Series of figures showing the GNSS time-series (left column) for east, north and vertical component and the model residuals (right column). The red lines represent the predictions of the trajectory model. The red lines and the green dots represent the predictions of the trajectory model and the daily observations, respectively. The model accounts for a linear, interseismic rate, antenna offsets (Cyan diamond symbol), earthquake offsets (fuchsia stars), postseismic decays, and seasonal oscillations.

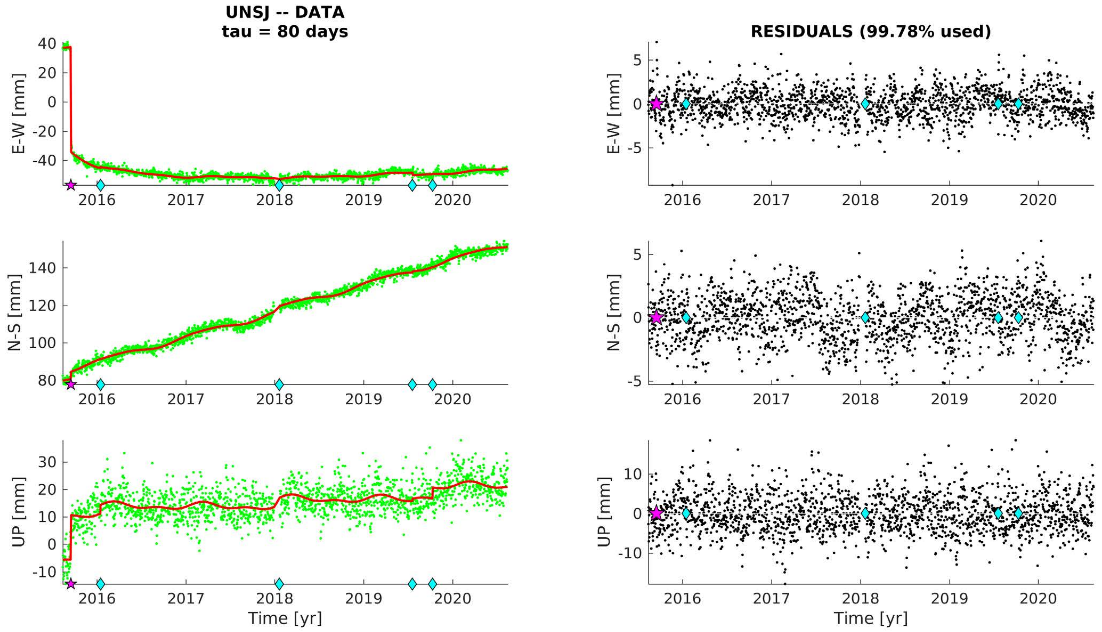

Figure S28. Series of figures showing the GNSS time-series (left column) for east, north and vertical component and the model residuals (right column). The red lines represent the predictions of the trajectory model. The red lines and the green dots represent the predictions of the trajectory model and the daily observations, respectively. The model accounts for a linear, interseismic rate, antenna offsets (Cyan diamond symbol), earthquake offsets (fuchsia stars), postseismic decays, and seasonal oscillations.

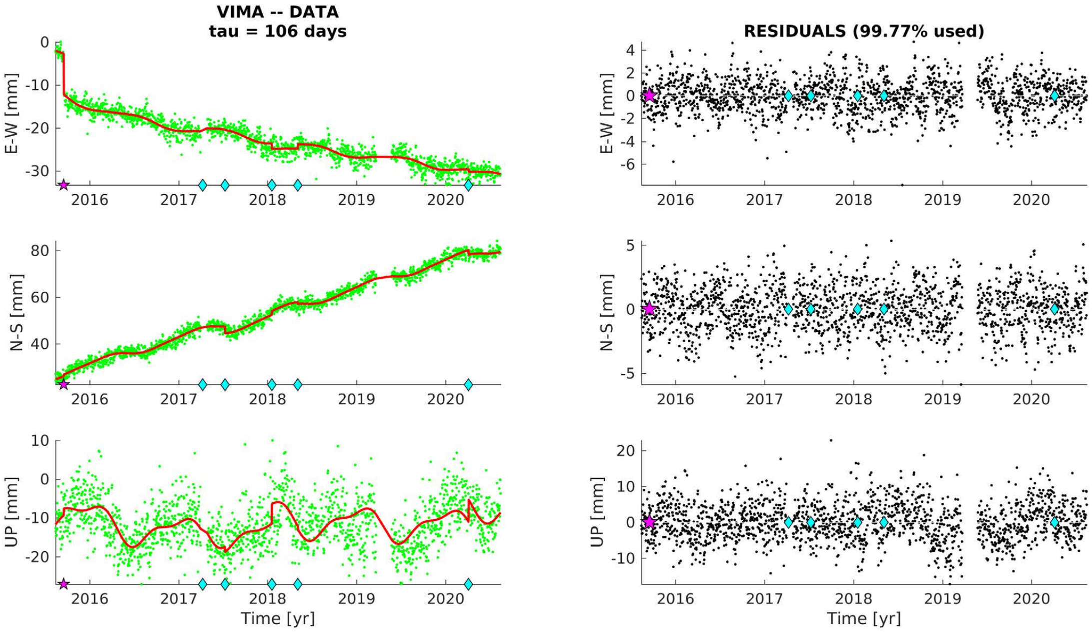

Figure S29. Series of figures showing the GNSS time-series (left column) for east, north and vertical component and the model residuals (right column). The red lines represent the predictions of the trajectory model. The red lines and the green dots represent the predictions of the trajectory model and the daily observations, respectively. The model accounts for a linear, interseismic rate, antenna offsets (Cyan diamond symbol), earthquake offsets (fuchsia stars), postseismic decays, and seasonal oscillations.

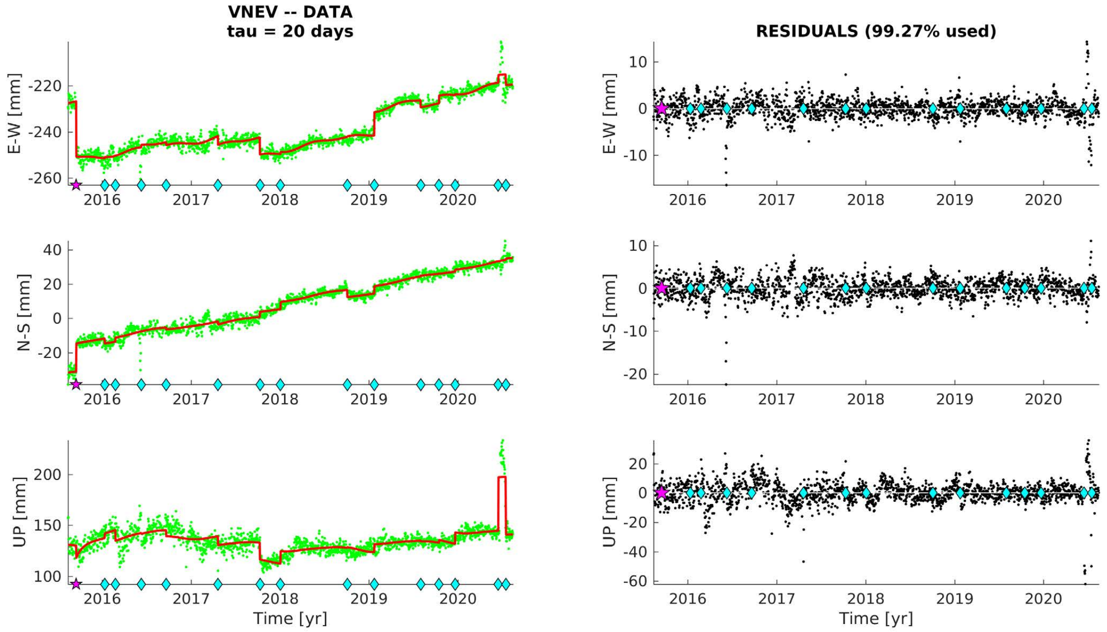

Figure S30. Series of figures showing the GNSS time-series (left column) for east, north and vertical component and the model residuals (right column). The red lines represent the predictions of the trajectory model. The red lines and the green dots represent the predictions of the trajectory model and the daily observations, respectively. The model accounts for a linear, interseismic rate, antenna offsets (Cyan diamond symbol), earthquake offsets (fuchsia stars), postseismic decays, and seasonal oscillations.

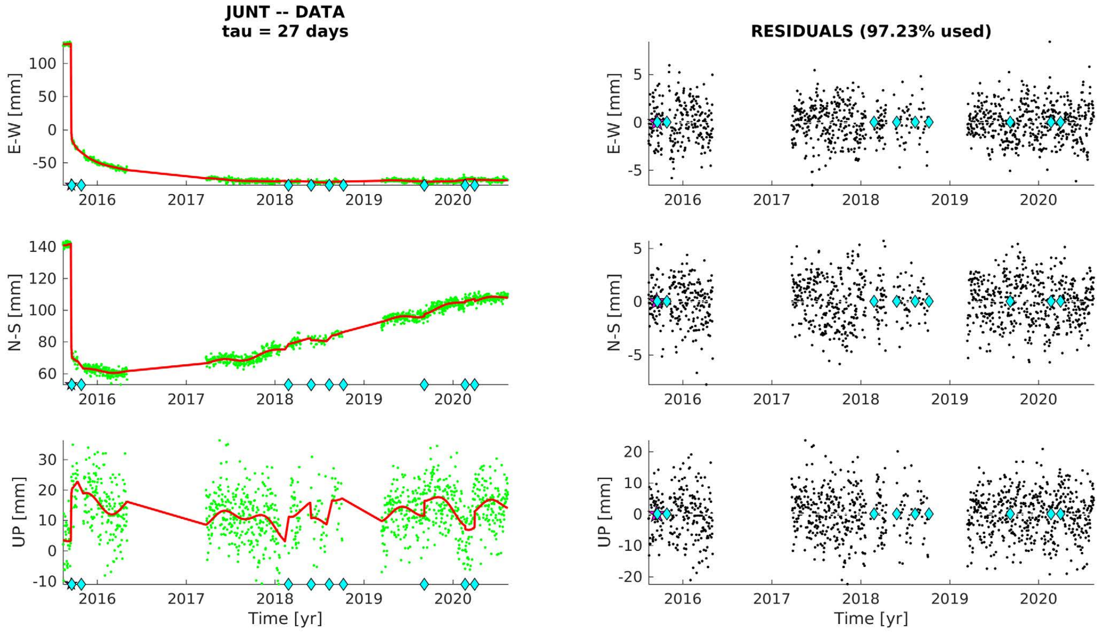

Figure S31. Series of figures showing the GNSS time-series (left column) for east, north and vertical component and the model residuals (right column). The red lines represent the predictions of the trajectory model. The red lines and the green dots represent the predictions of the trajectory model and the daily observations, respectively. The model accounts for a linear, interseismic rate, antenna offsets (Cyan diamond symbol), earthquake offsets (fuchsia stars), postseismic decays, and seasonal oscillations.

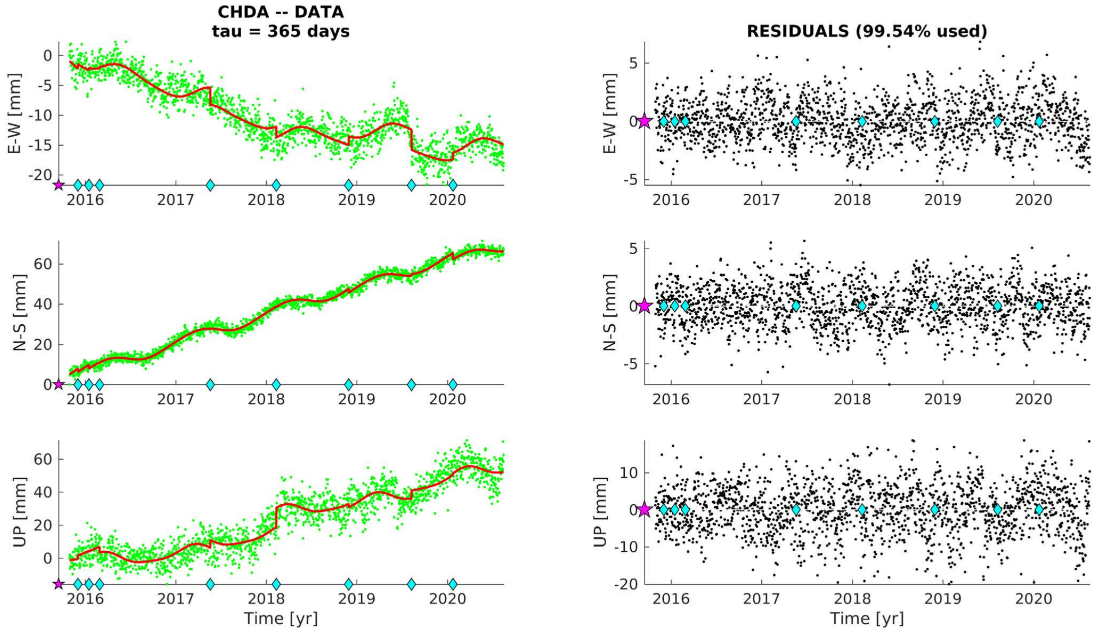

Figure S32. Series of figures showing the GNSS time-series (left column) for east, north and vertical component and the model residuals (right column). The red lines represent the predictions of the trajectory model. The red lines and the green dots represent the predictions of the trajectory model and the daily observations, respectively. The model accounts for a linear, interseismic rate, antenna offsets (Cyan diamond symbol), earthquake offsets (fuchsia stars), postseismic decays, and seasonal oscillations.

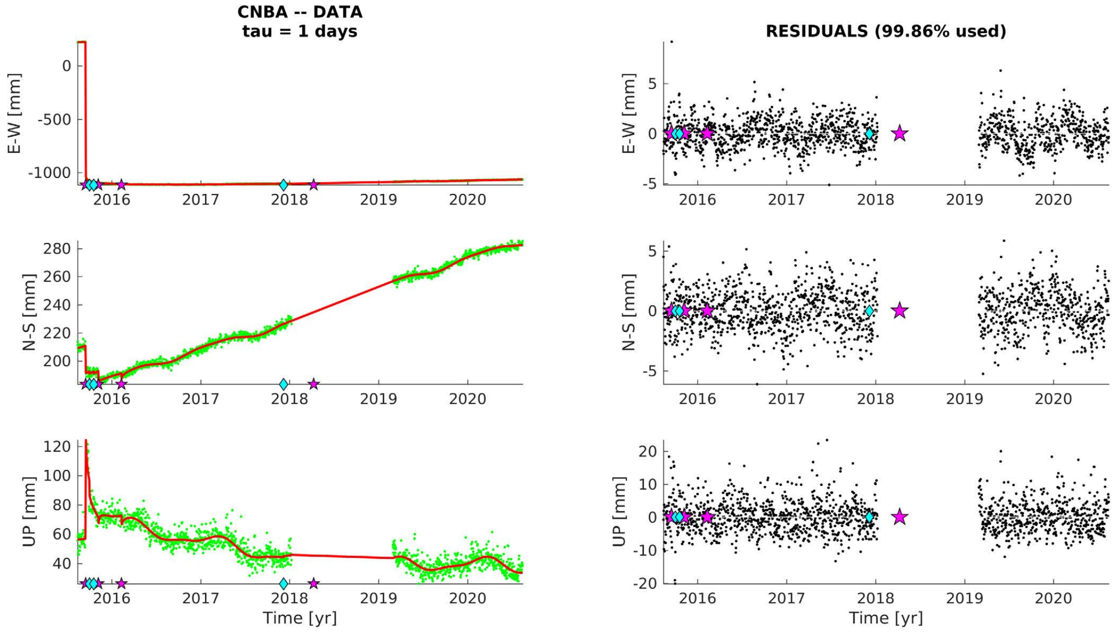

Figure S33. Series of figures showing the GNSS time-series (left column) for east, north and vertical component and the model residuals (right column). The red lines represent the predictions of the trajectory model. The red lines and the green dots represent the predictions of the trajectory model and the daily observations, respectively. The model accounts for a linear, interseismic rate, antenna offsets (Cyan diamond symbol), earthquake offsets (fuchsia stars), postseismic decays, and seasonal oscillations.

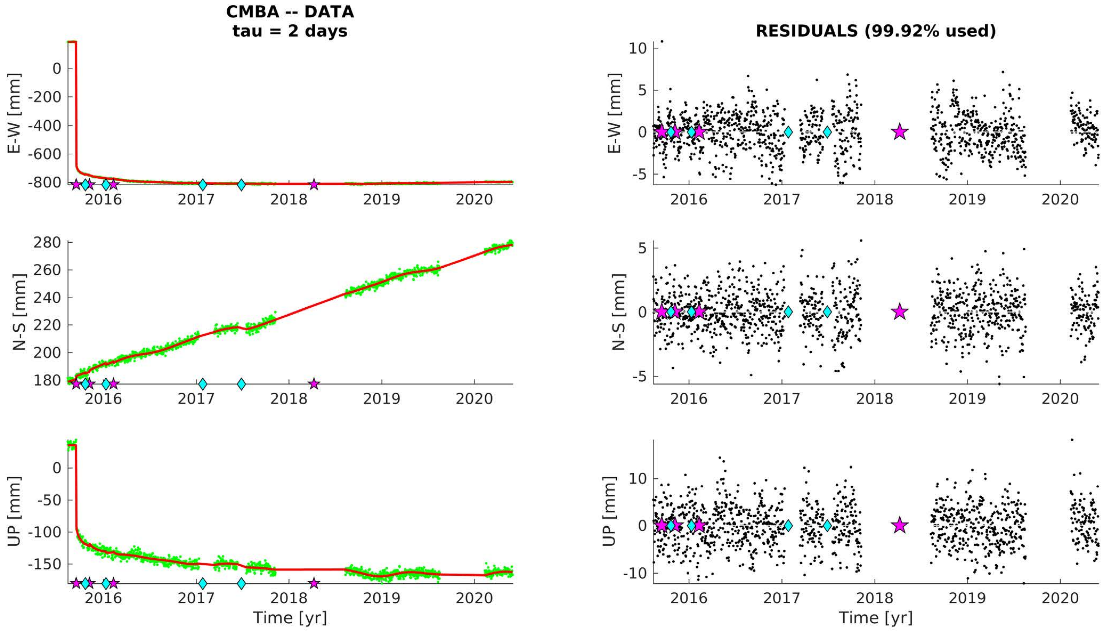

Figure S34. Series of figures showing the GNSS time-series (left column) for east, north and vertical component and the model residuals (right column). The red lines represent the predictions of the trajectory model. The red lines and the green dots represent the predictions of the trajectory model and the daily observations, respectively. The model accounts for a linear, interseismic rate, antenna offsets (Cyan diamond symbol), earthquake offsets (fuchsia stars), postseismic decays, and seasonal oscillations.

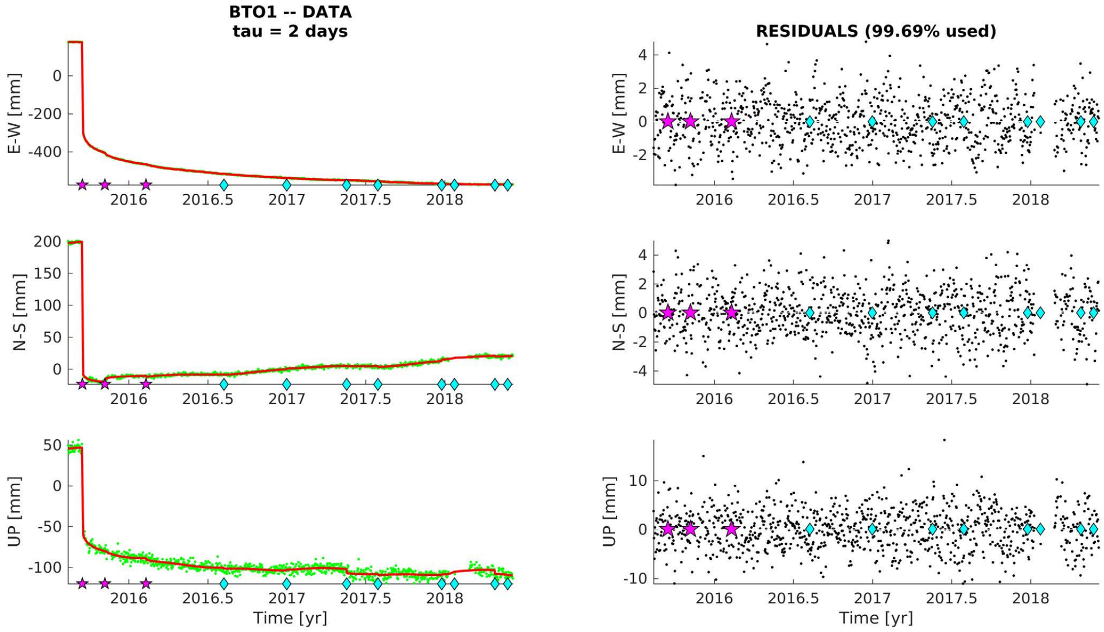

Figure S35. Series of figures showing the GNSS time-series (left column) for east, north and vertical component and the model residuals (right column). The red lines represent the predictions of the trajectory model. The red lines and the green dots represent the predictions of the trajectory model and the daily observations, respectively. The model accounts for a linear, interseismic rate, antenna offsets (Cyan diamond symbol), earthquake offsets (fuchsia stars), postseismic decays, and seasonal oscillations.

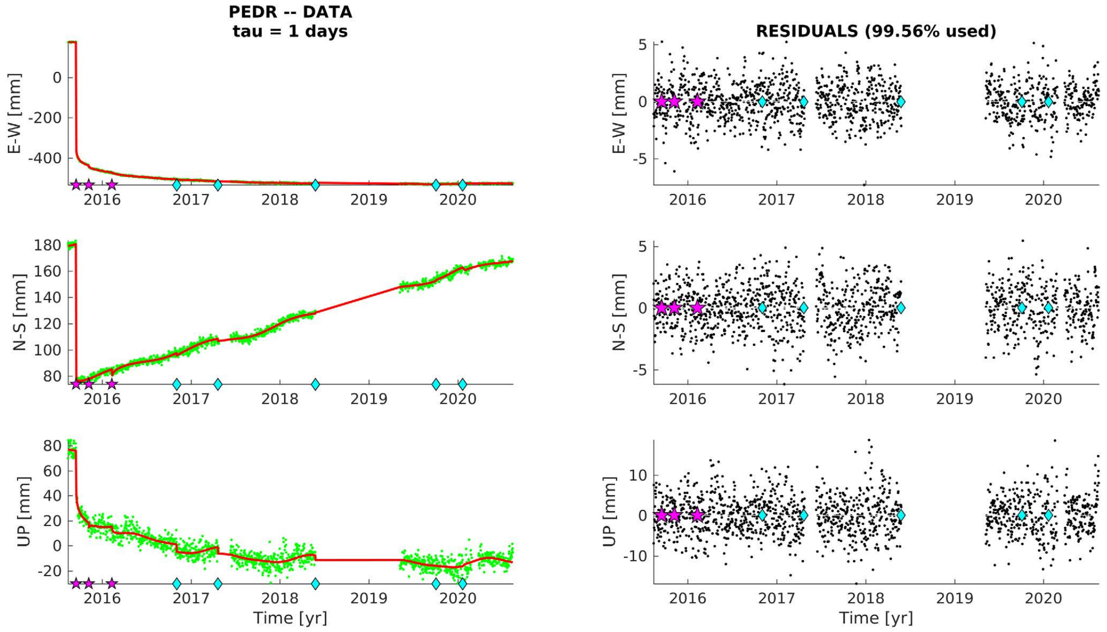

Figure S36. Series of figures showing the GNSS time-series (left column) for east, north and vertical component and the model residuals (right column). The red lines represent the predictions of the trajectory model. The red lines and the green dots represent the predictions of the trajectory model and the daily observations, respectively. The model accounts for a linear, interseismic rate, antenna offsets (Cyan diamond symbol), earthquake offsets (fuchsia stars), postseismic decays, and seasonal oscillations.

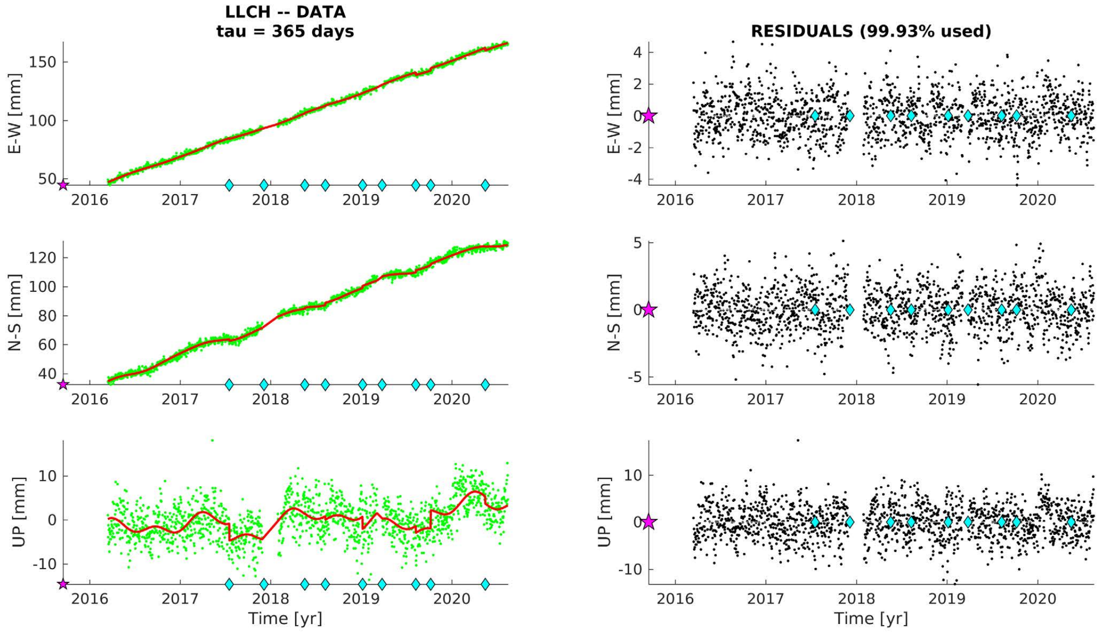

Figure S37. Series of figures showing the GNSS time-series (left column) for east, north and vertical component and the model residuals (right column). The red lines represent the predictions of the trajectory model. The red lines and the green dots represent the predictions of the trajectory model and the daily observations, respectively. The model accounts for a linear, interseismic rate, antenna offsets (Cyan diamond symbol), earthquake offsets (fuchsia stars), postseismic decays, and seasonal oscillations.

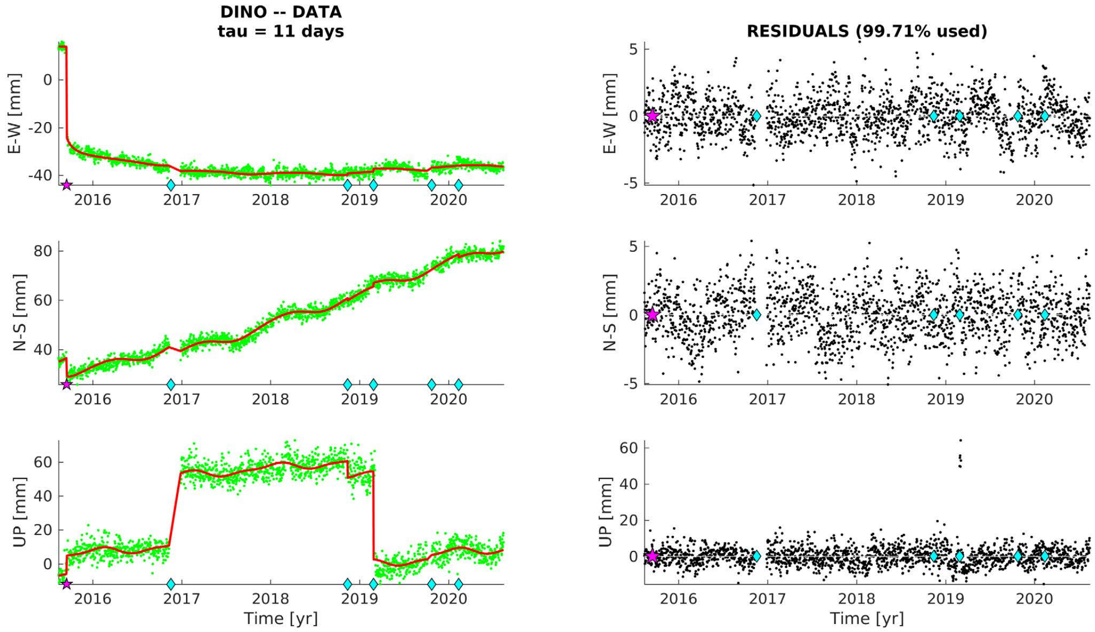

Figure S38. Series of figures showing the GNSS time-series (left column) for east, north and vertical component and the model residuals (right column). The red lines represent the predictions of the trajectory model. The red lines and the green dots represent the predictions of the trajectory model and the daily observations, respectively. The model accounts for a linear, interseismic rate, antenna offsets (Cyan diamond symbol), earthquake offsets (fuchsia stars), postseismic decays, and seasonal oscillations.

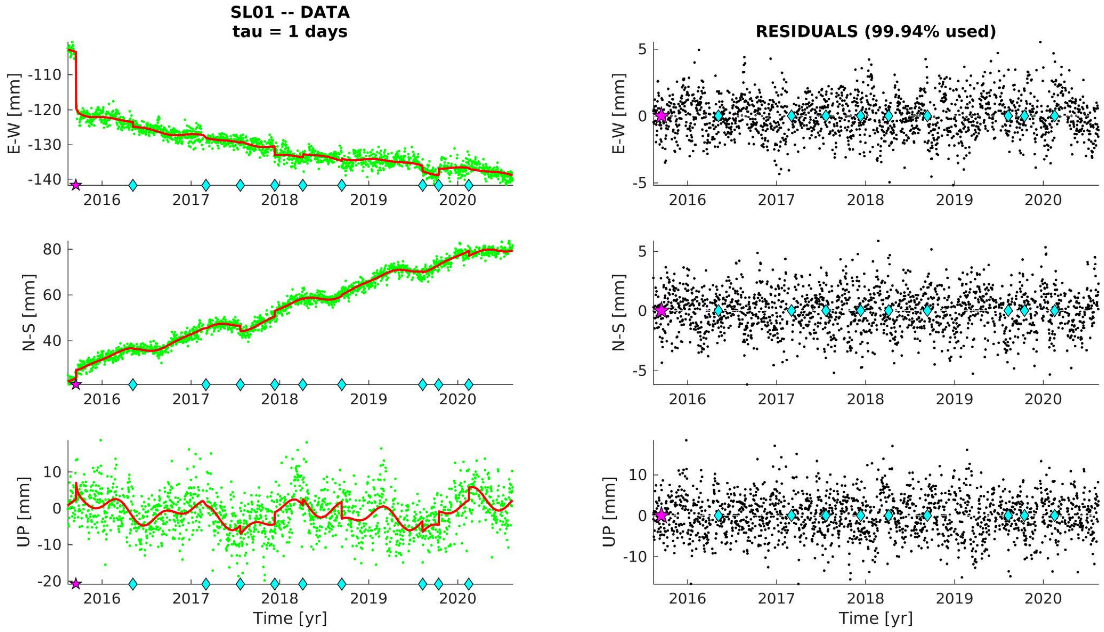

Figure S39. Series of figures showing the GNSS time-series (left column) for east, north and vertical component and the model residuals (right column). The red lines represent the predictions of the trajectory model. The red lines and the green dots represent the predictions of the trajectory model and the daily observations, respectively. The model accounts for a linear, interseismic rate, antenna offsets (Cyan diamond symbol), earthquake offsets (fuchsia stars), postseismic decays, and seasonal oscillations.

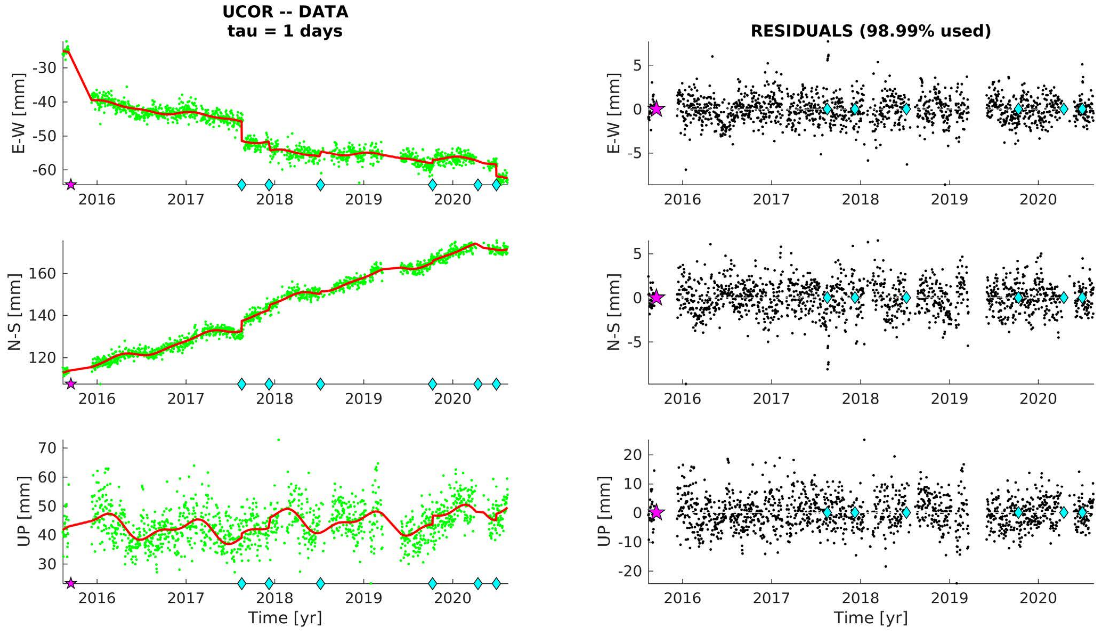

Figure S40. Series of figures showing the GNSS time-series (left column) for east, north and vertical component and the model residuals (right column). The red lines represent the predictions of the trajectory model. The red lines and the green dots represent the predictions of the trajectory model and the daily observations, respectively. The model accounts for a linear, interseismic rate, antenna offsets (Cyan diamond symbol), earthquake offsets (fuchsia stars), postseismic decays, and seasonal oscillations.

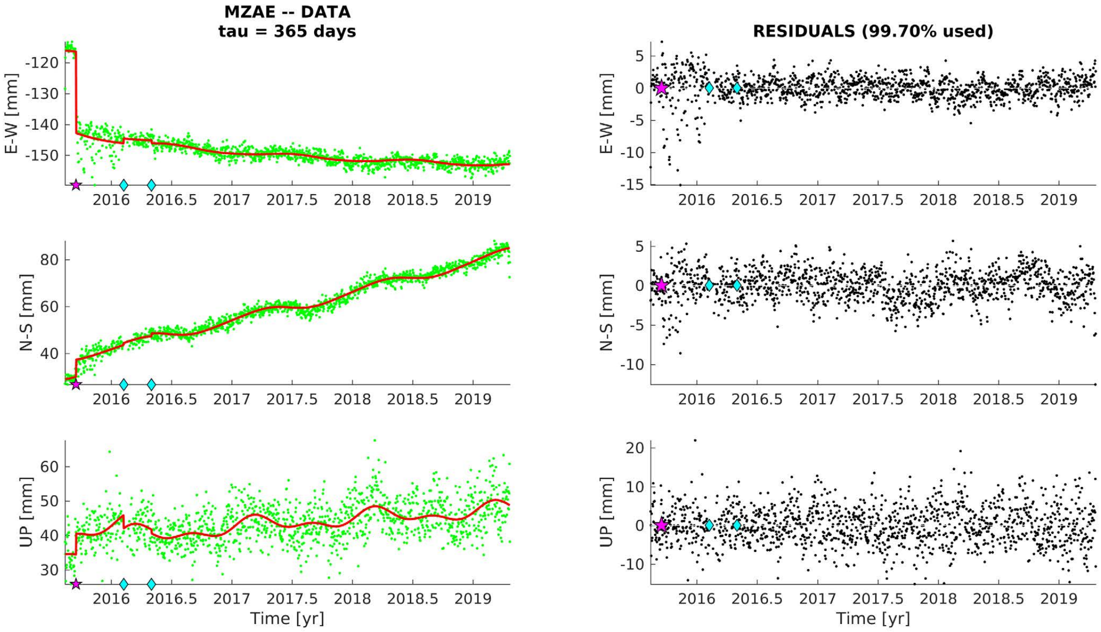

Figure S41. Series of figures showing the GNSS time-series (left column) for east, north and vertical component and the model residuals (right column). The red lines represent the predictions of the trajectory model. The red lines and the green dots represent the predictions of the trajectory model and the daily observations, respectively. The model accounts for a linear, interseismic rate, antenna offsets (Cyan diamond symbol), earthquake offsets (fuchsia stars), postseismic decays, and seasonal oscillations.

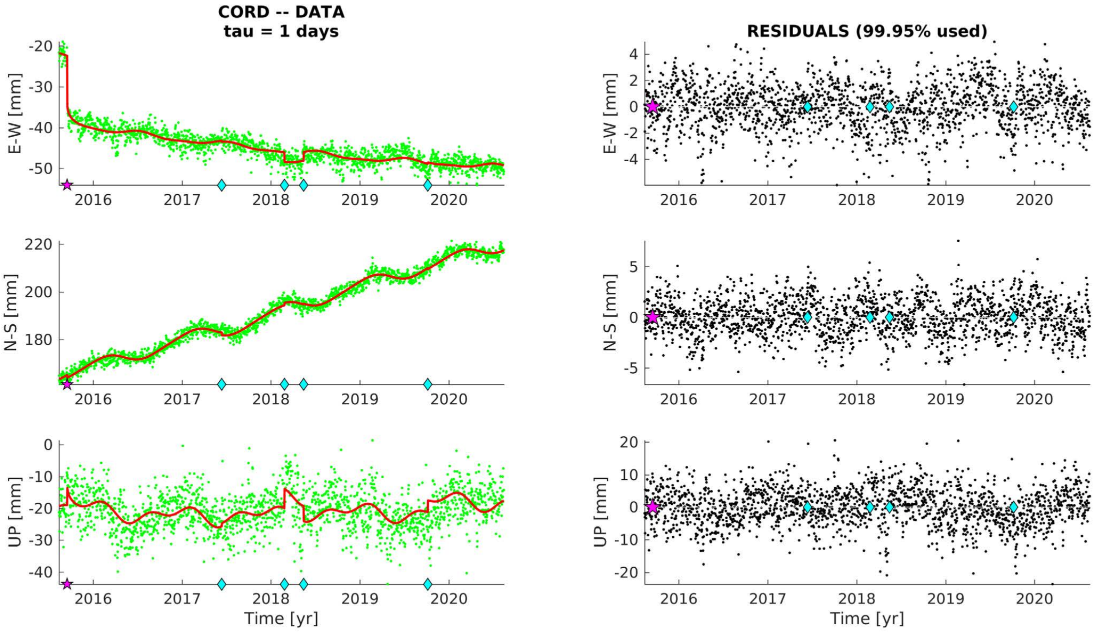

Figure S42. Series of figures showing the GNSS time-series (left column) for east, north and vertical component and the model residuals (right column). The red lines represent the predictions of the trajectory model. The red lines and the green dots represent the predictions of the trajectory model and the daily observations, respectively. The model accounts for a linear, interseismic rate, antenna offsets (Cyan diamond symbol), earthquake offsets (fuchsia stars), postseismic decays, and seasonal oscillations.

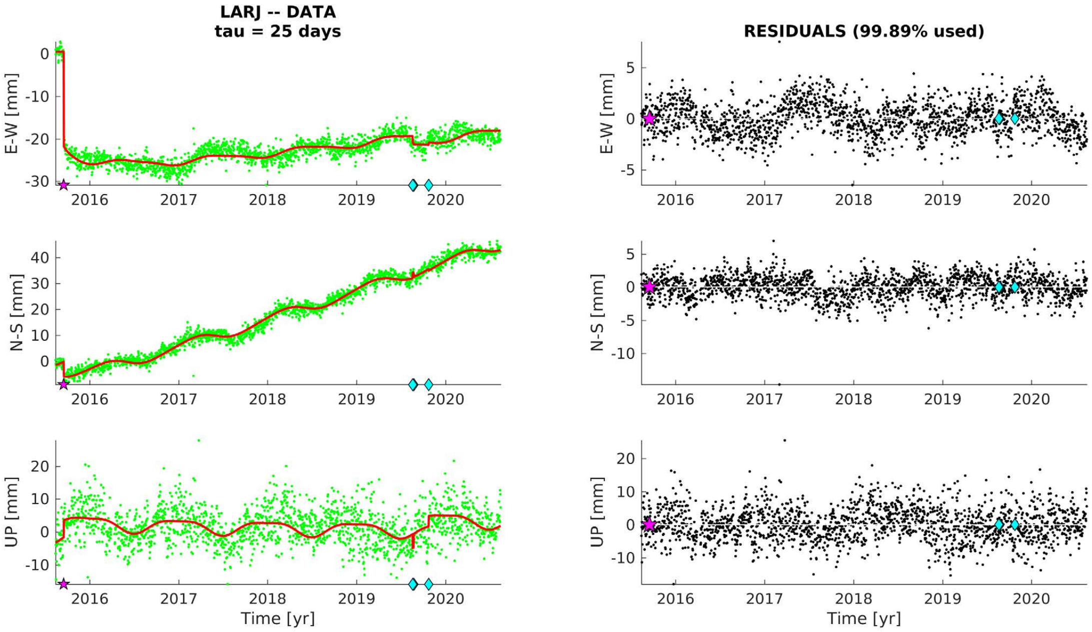

Figure S43. Series of figures showing the GNSS time-series (left column) for east, north and vertical component and the model residuals (right column). The red lines represent the predictions of the trajectory model. The red lines and the green dots represent the predictions of the trajectory model and the daily observations, respectively. The model accounts for a linear, interseismic rate, antenna offsets (Cyan diamond symbol), earthquake offsets (fuchsia stars), postseismic decays, and seasonal oscillations.

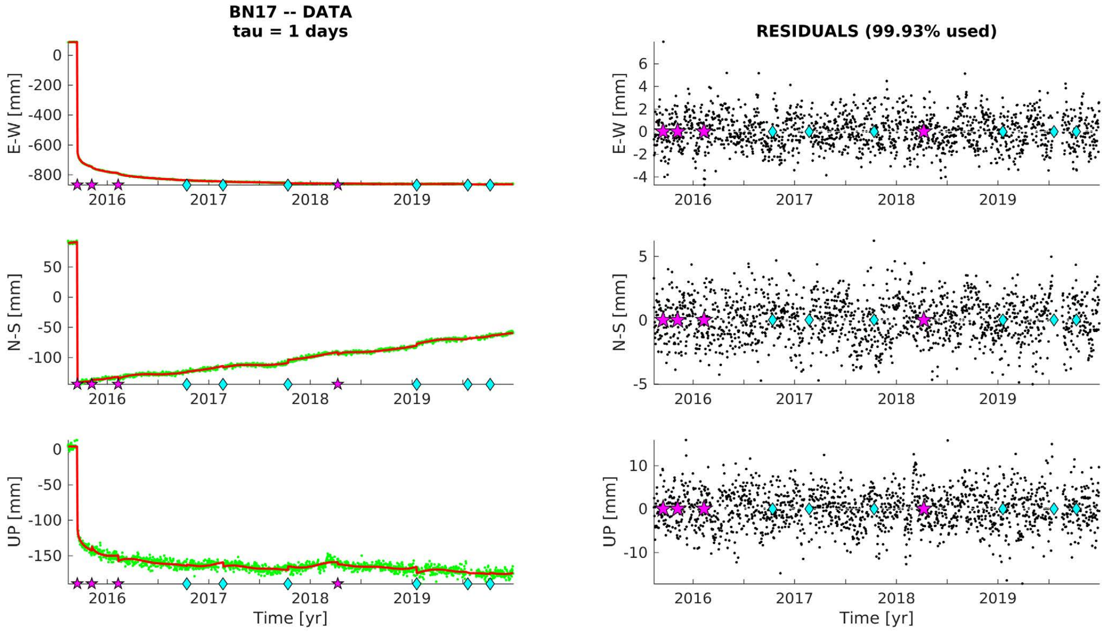

Figure S44. Series of figures showing the GNSS time-series (left column) for east, north and vertical component and the model residuals (right column). The red lines represent the predictions of the trajectory model. The red lines and the green dots represent the predictions of the trajectory model and the daily observations, respectively. The model accounts for a linear, interseismic rate, antenna offsets (Cyan diamond symbol), earthquake offsets (fuchsia stars), postseismic decays, and seasonal oscillations.

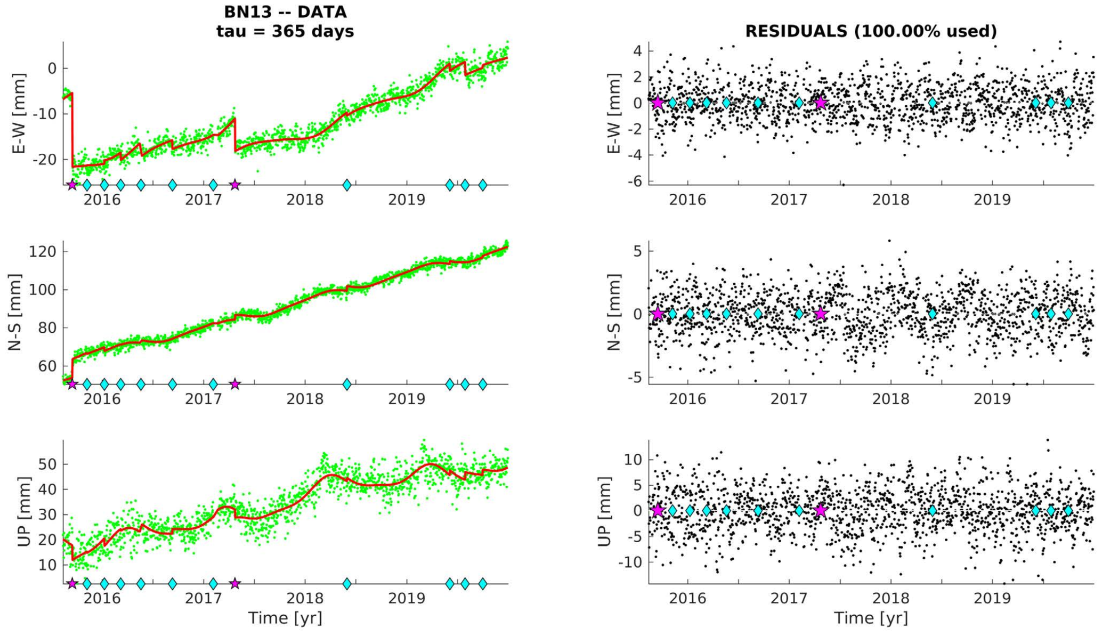

Figure S45. Series of figures showing the GNSS time-series (left column) for east, north and vertical component and the model residuals (right column). The red lines represent the predictions of the trajectory model. The red lines and the green dots represent the predictions of the trajectory model and the daily observations, respectively. The model accounts for a linear, interseismic rate, antenna offsets (Cyan diamond symbol), earthquake offsets (fuchsia stars), postseismic decays, and seasonal oscillations.

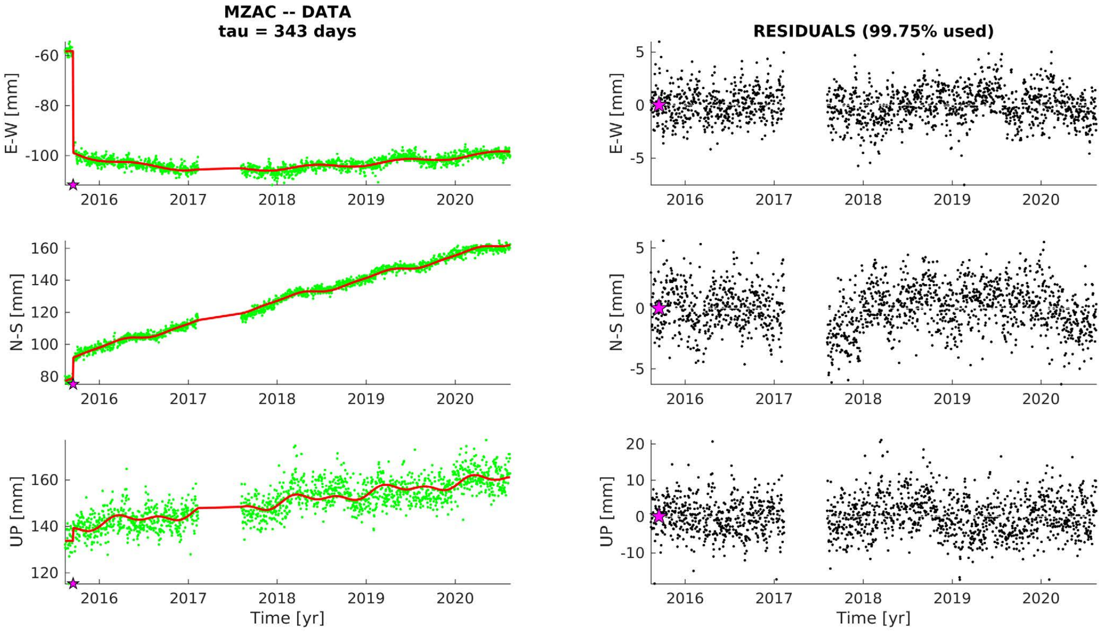

Figure S46. Series of figures showing the GNSS time-series (left column) for east, north and vertical component and the model residuals (right column). The red lines represent the predictions of the trajectory model. The red lines and the green dots represent the predictions of the trajectory model and the daily observations, respectively. The model accounts for a linear, interseismic rate, antenna offsets (Cyan diamond symbol), earthquake offsets (fuchsia stars), postseismic decays, and seasonal oscillations.

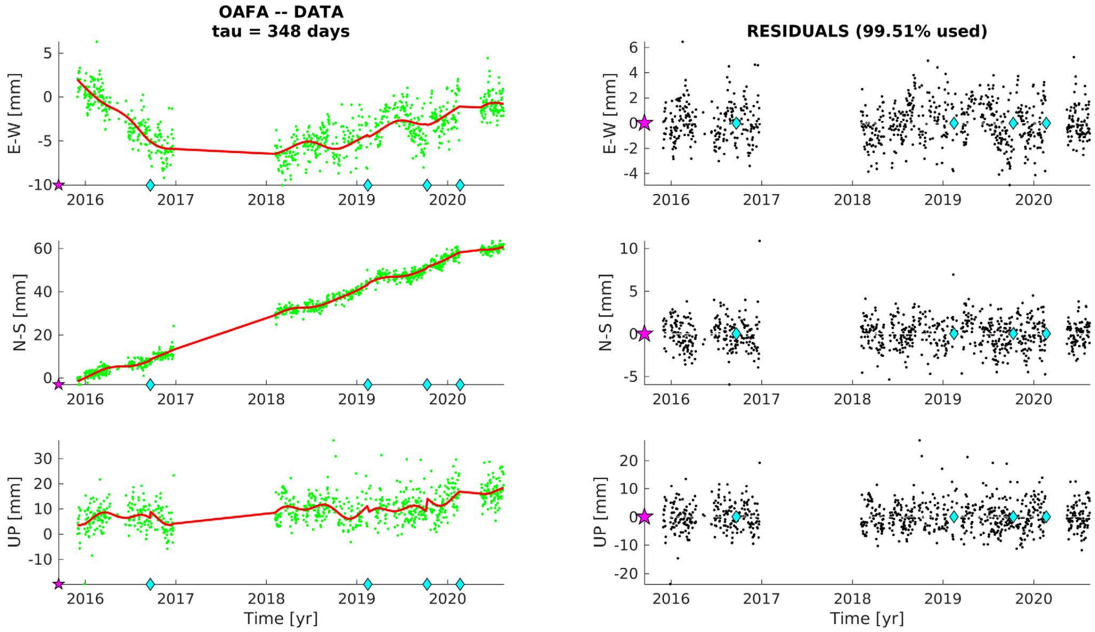

Figure S47. Series of figures showing the GNSS time-series (left column) for east, north and vertical component and the model residuals (right column). The red lines represent the predictions of the trajectory model. The red lines and the green dots represent the predictions of the trajectory model and the daily observations, respectively. The model accounts for a linear, interseismic rate, antenna offsets (Cyan diamond symbol), earthquake offsets (fuchsia stars), postseismic decays, and seasonal oscillations.

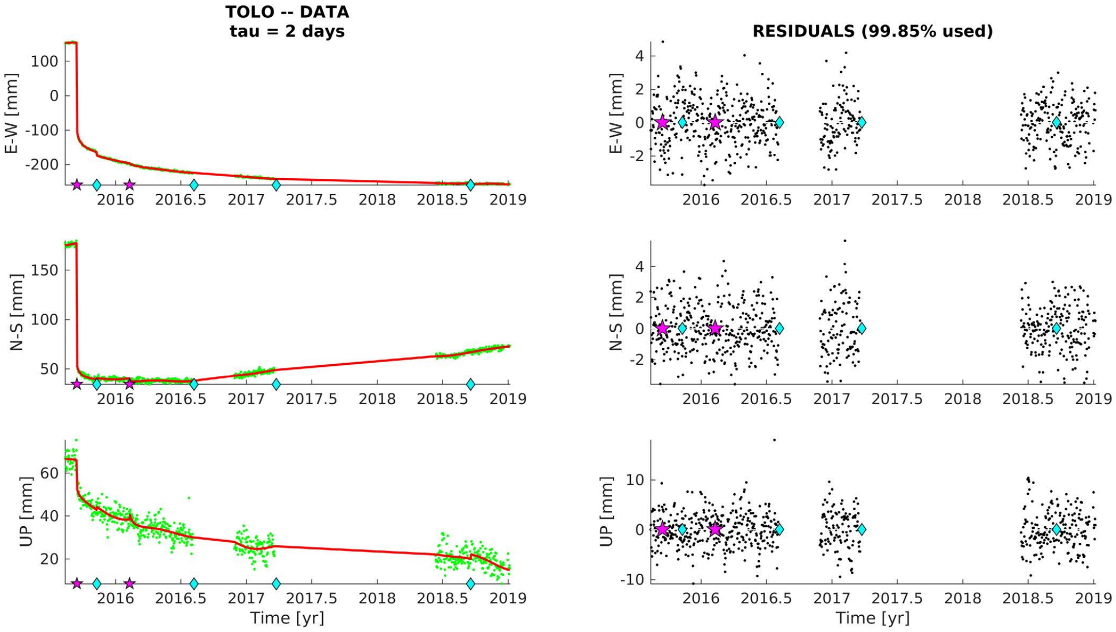

Figure S48. Series of figures showing the GNSS time-series (left column) for east, north and vertical component and the model residuals (right column). The red lines represent the predictions of the trajectory model. The red lines and the green dots represent the predictions of the trajectory model and the daily observations, respectively. The model accounts for a linear, interseismic rate, antenna offsets (Cyan diamond symbol), earthquake offsets (fuchsia stars), postseismic decays, and seasonal oscillations.

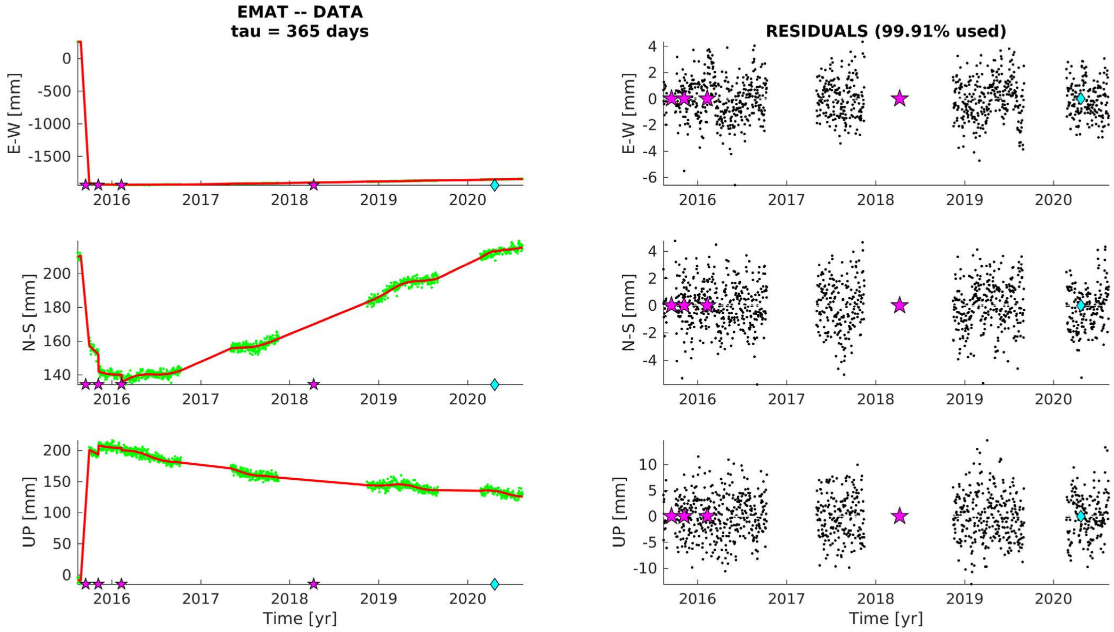

Figure S49. Series of figures showing the GNSS time-series (left column) for east, north and vertical component and the model residuals (right column). The red lines represent the predictions of the trajectory model. The red lines and the green dots represent the predictions of the trajectory model and the daily observations, respectively. The model accounts for a linear, interseismic rate, antenna offsets (Cyan diamond symbol), earthquake offsets (fuchsia stars), postseismic decays, and seasonal oscillations.

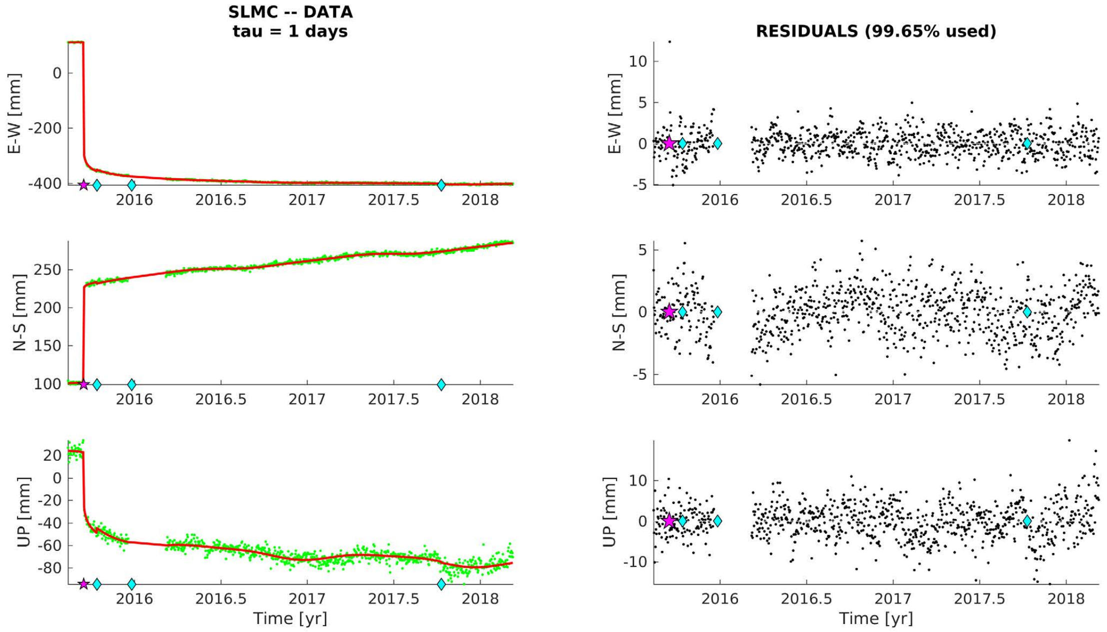

Figure S50. Series of figures showing the GNSS time-series (left column) for east, north and vertical component and the model residuals (right column). The red lines represent the predictions of the trajectory model. The red lines and the green dots represent the predictions of the trajectory model and the daily observations, respectively. The model accounts for a linear, interseismic rate, antenna offsets (Cyan diamond symbol), earthquake offsets (fuchsia stars), postseismic decays, and seasonal oscillations.

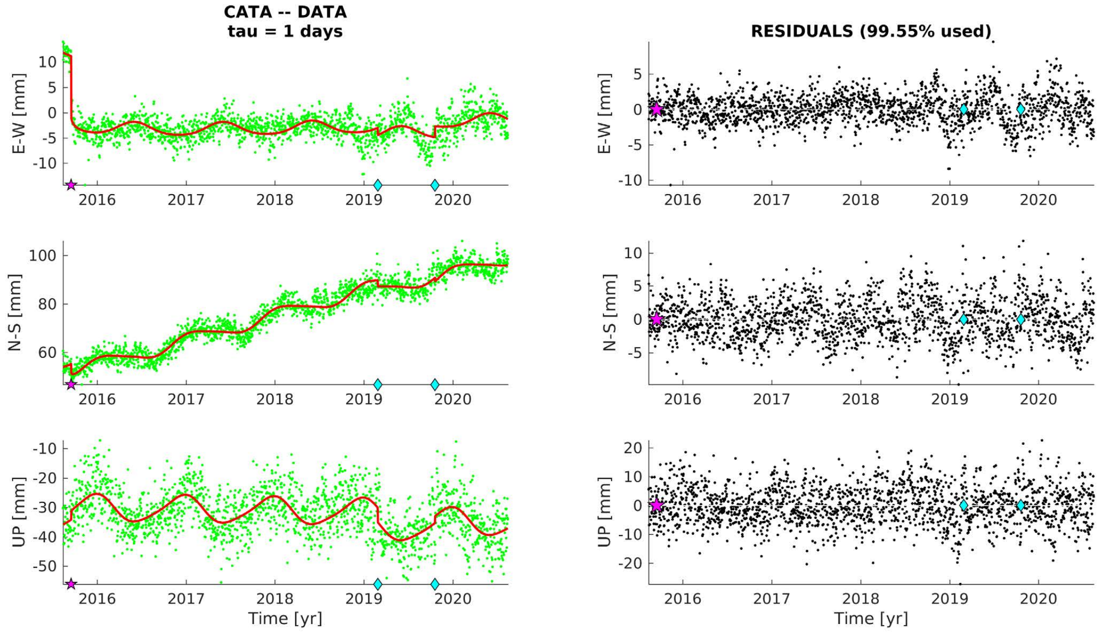

Figure S51. Series of figures showing the GNSS time-series (left column) for east, north and vertical component and the model residuals (right column). The red lines represent the predictions of the trajectory model. The red lines and the green dots represent the predictions of the trajectory model and the daily observations, respectively. The model accounts for a linear, interseismic rate, antenna offsets (Cyan diamond symbol), earthquake offsets (fuchsia stars), postseismic decays, and seasonal oscillations.

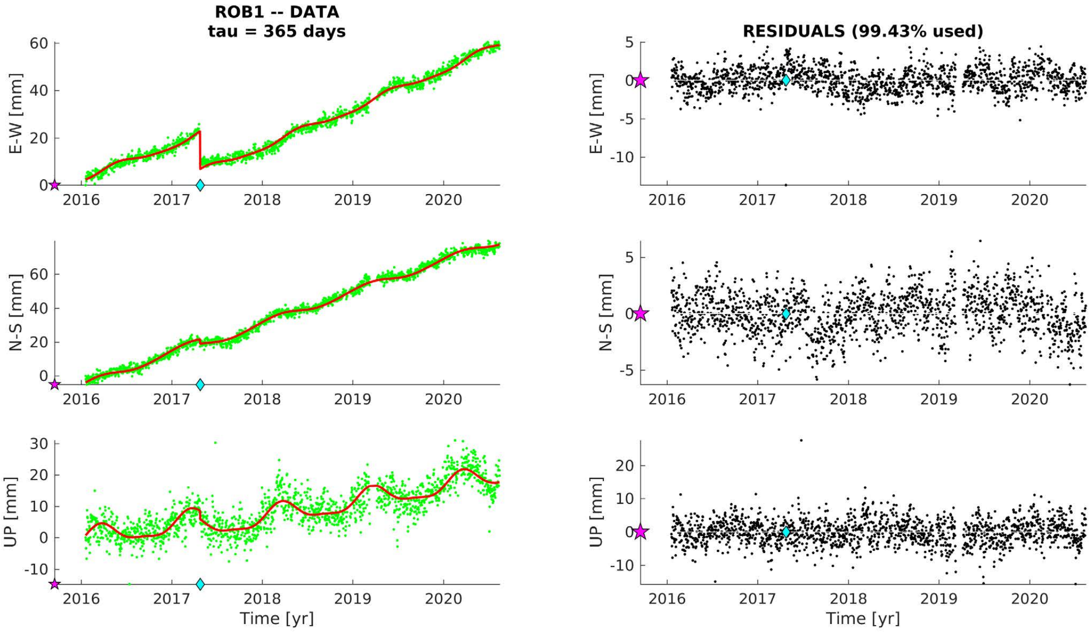

Figure S52. Series of figures showing the GNSS time-series (left column) for east, north and vertical component and the model residuals (right column). The red lines represent the predictions of the trajectory model. The red lines and the green dots represent the predictions of the trajectory model and the daily observations, respectively. The model accounts for a linear, interseismic rate, antenna offsets (Cyan diamond symbol), earthquake offsets (fuchsia stars), postseismic decays, and seasonal oscillations.

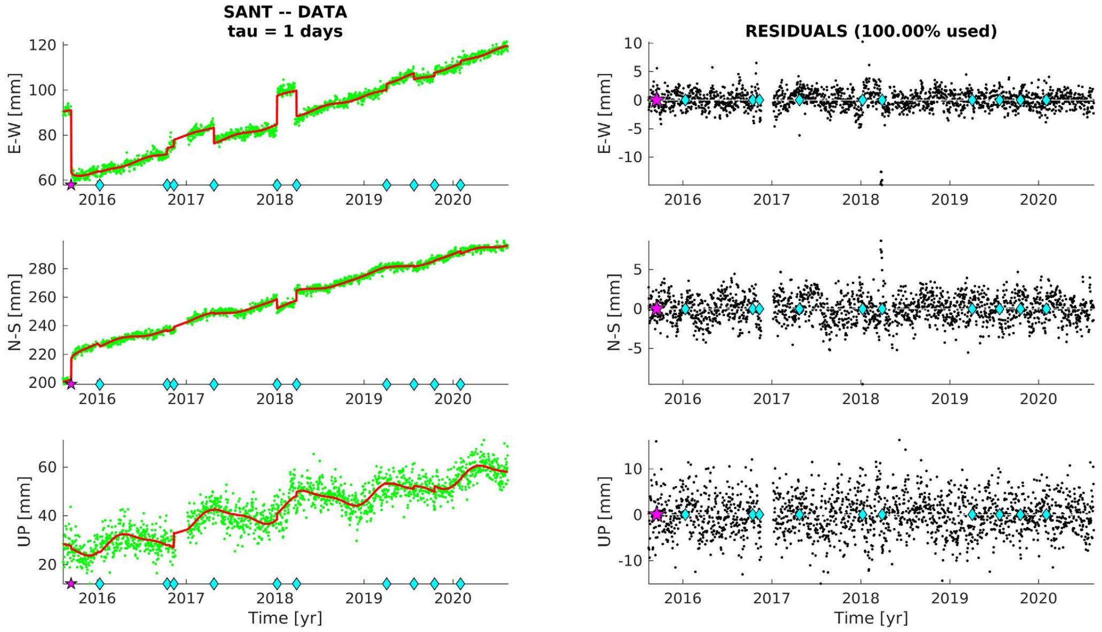

Figure S53. Series of figures showing the GNSS time-series (left column) for east, north and vertical component and the model residuals (right column). The red lines represent the predictions of the trajectory model. The red lines and the green dots represent the predictions of the trajectory model and the daily observations, respectively. The model accounts for a linear, interseismic rate, antenna offsets (Cyan diamond symbol), earthquake offsets (fuchsia stars), postseismic decays, and seasonal oscillations.

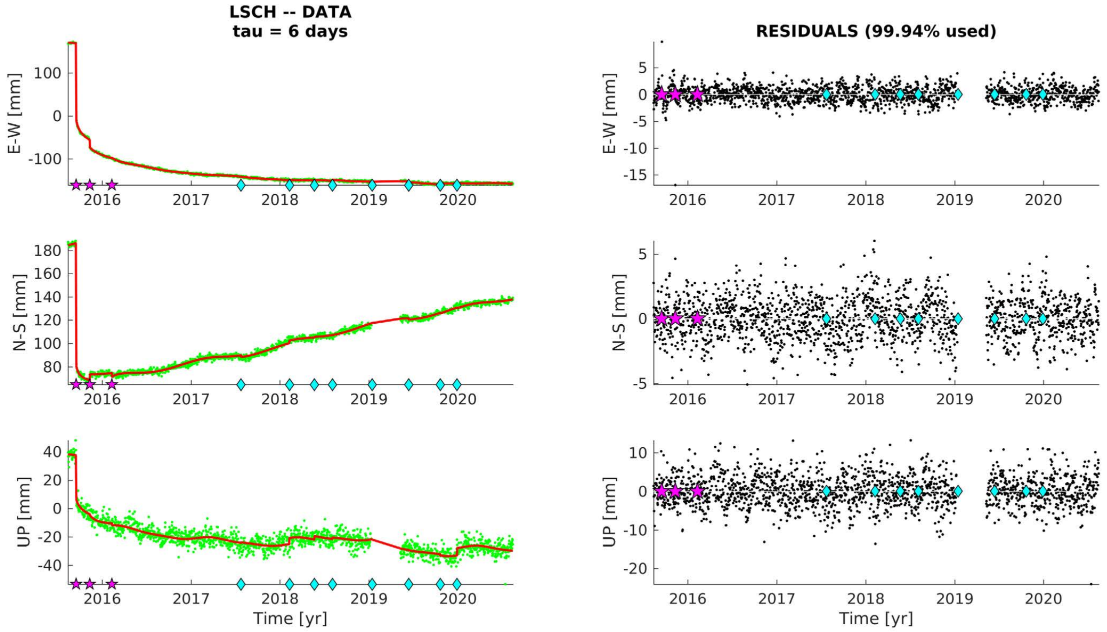

Figure S54. Series of figures showing the GNSS time-series (left column) for east, north and vertical component and the model residuals (right column). The red lines represent the predictions of the trajectory model. The red lines and the green dots represent the predictions of the trajectory model and the daily observations, respectively. The model accounts for a linear, interseismic rate, antenna offsets (Cyan diamond symbol), earthquake offsets (fuchsia stars), postseismic decays, and seasonal oscillations.

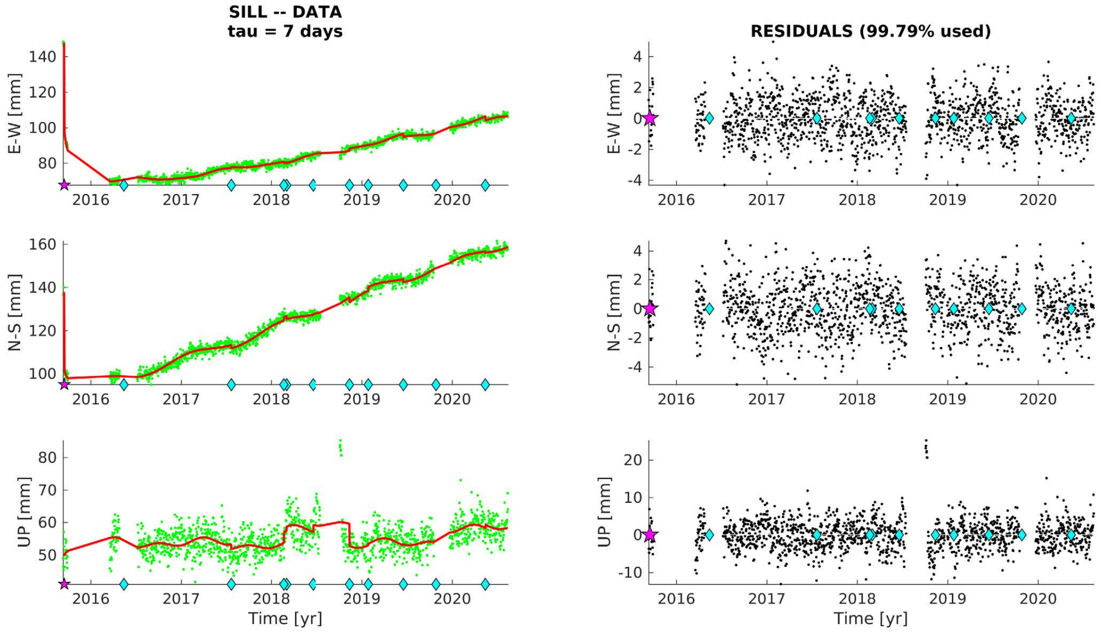

Figure S55. Series of figures showing the GNSS time-series (left column) for east, north and vertical component and the model residuals (right column). The red lines represent the predictions of the trajectory model. The red lines and the green dots represent the predictions of the trajectory model and the daily observations, respectively. The model accounts for a linear, interseismic rate, antenna offsets (Cyan diamond symbol), earthquake offsets (fuchsia stars), postseismic decays, and seasonal oscillations.

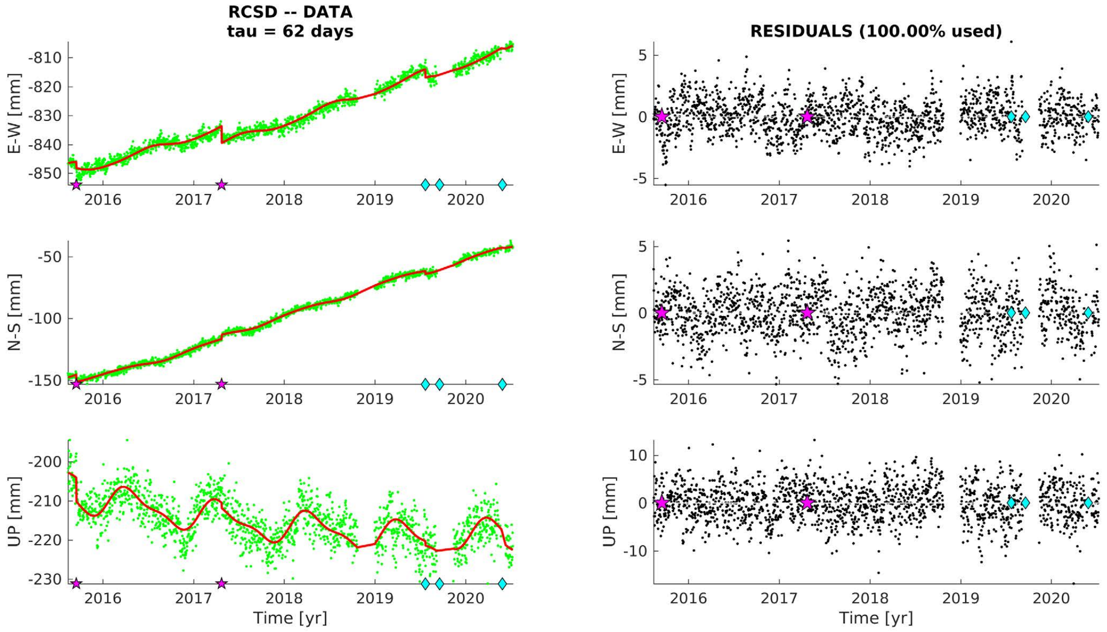

Figure S56. Series of figures showing the GNSS time-series (left column) for east, north and vertical component and the model residuals (right column). The red lines represent the predictions of the trajectory model. The red lines and the green dots represent the predictions of the trajectory model and the daily observations, respectively. The model accounts for a linear, interseismic rate, antenna offsets (Cyan diamond symbol), earthquake offsets (fuchsia stars), postseismic decays, and seasonal oscillations.

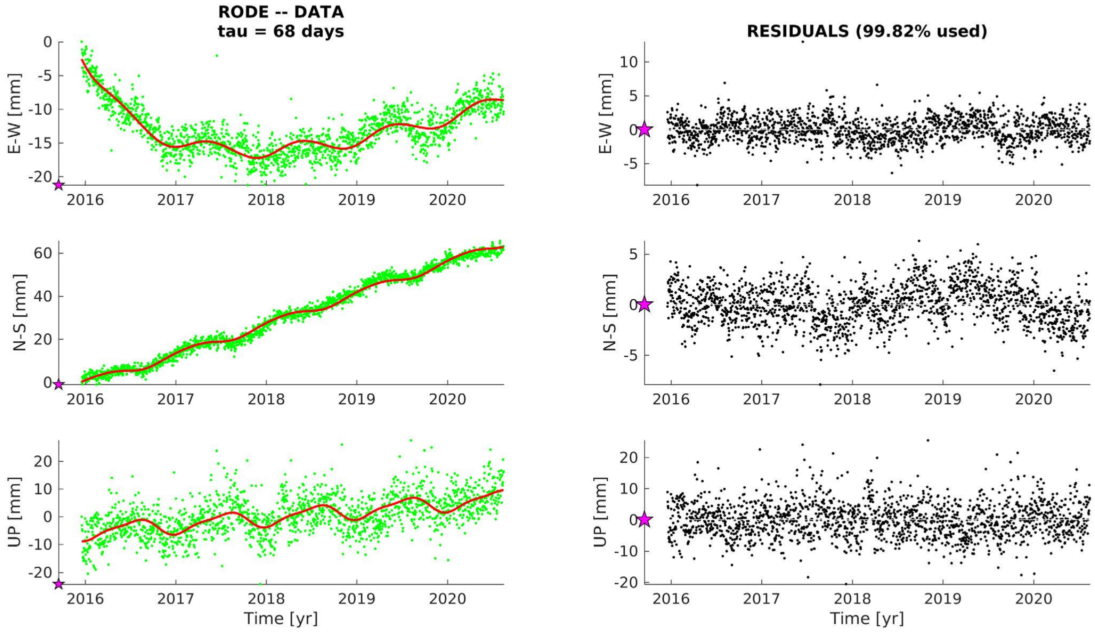

Figure S57. Series of figures showing the GNSS time-series (left column) for east, north and vertical component and the model residuals (right column). The red lines represent the predictions of the trajectory model. The red lines and the green dots represent the predictions of the trajectory model and the daily observations, respectively. The model accounts for a linear, interseismic rate, antenna offsets (Cyan diamond symbol), earthquake offsets (fuchsia stars), postseismic decays, and seasonal oscillations.

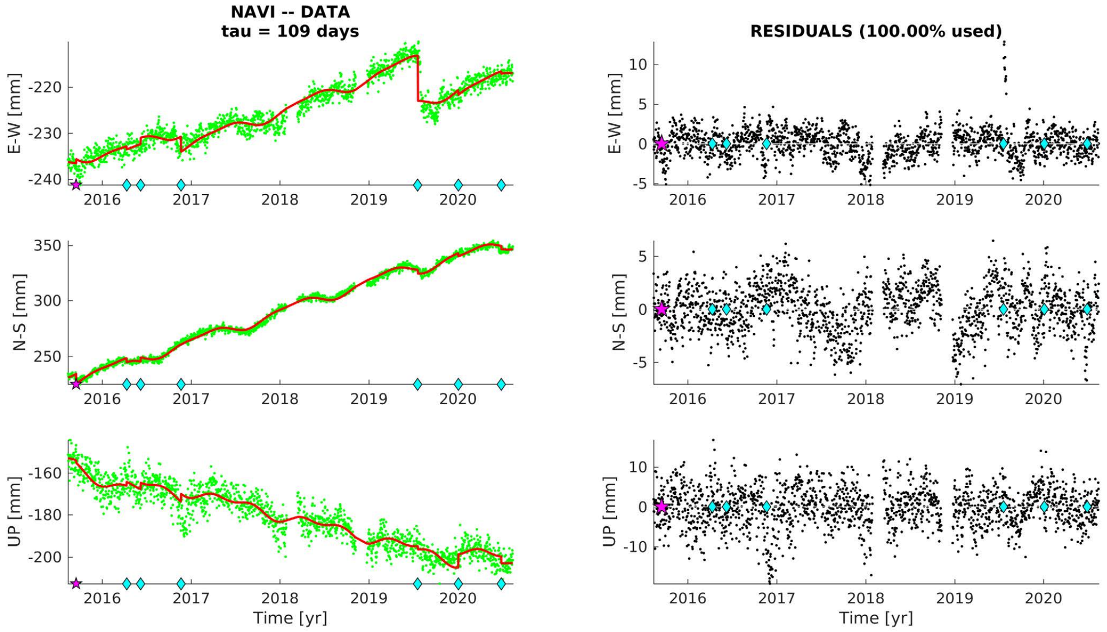

Figure S58. Series of figures showing the GNSS time-series (left column) for east, north and vertical component and the model residuals (right column). The red lines represent the predictions of the trajectory model. The red lines and the green dots represent the predictions of the trajectory model and the daily observations, respectively. The model accounts for a linear, interseismic rate, antenna offsets (Cyan diamond symbol), earthquake offsets (fuchsia stars), postseismic decays, and seasonal oscillations.

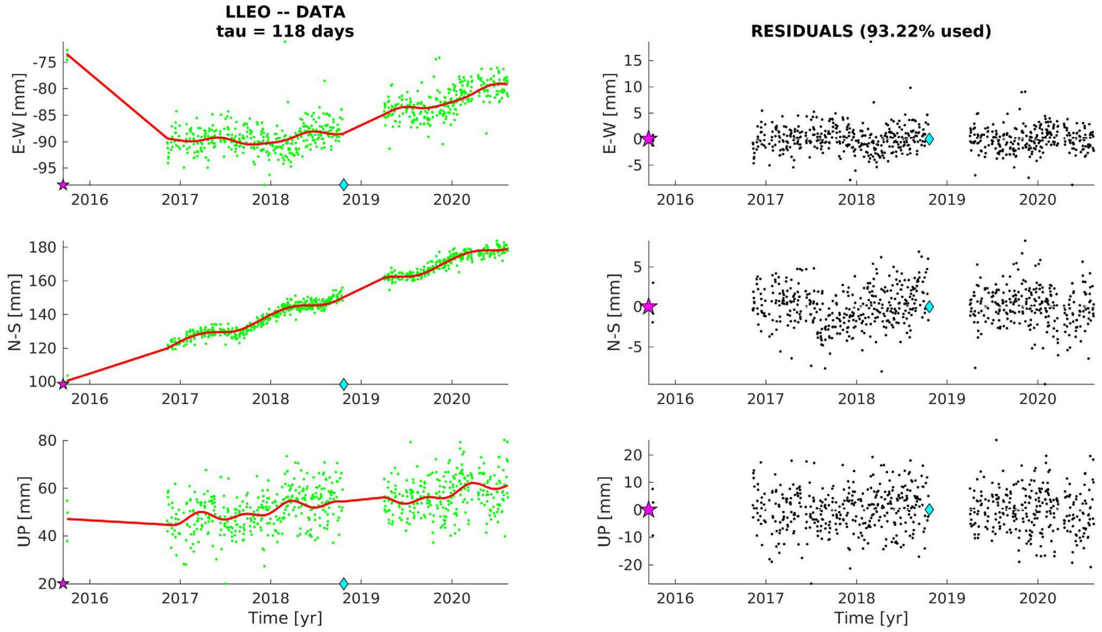

Figure S59. Series of figures showing the GNSS time-series (left column) for east, north and vertical component and the model residuals (right column). The red lines represent the predictions of the trajectory model. The red lines and the green dots represent the predictions of the trajectory model and the daily observations, respectively. The model accounts for a linear, interseismic rate, antenna offsets (Cyan diamond symbol), earthquake offsets (fuchsia stars), postseismic decays, and seasonal oscillations.

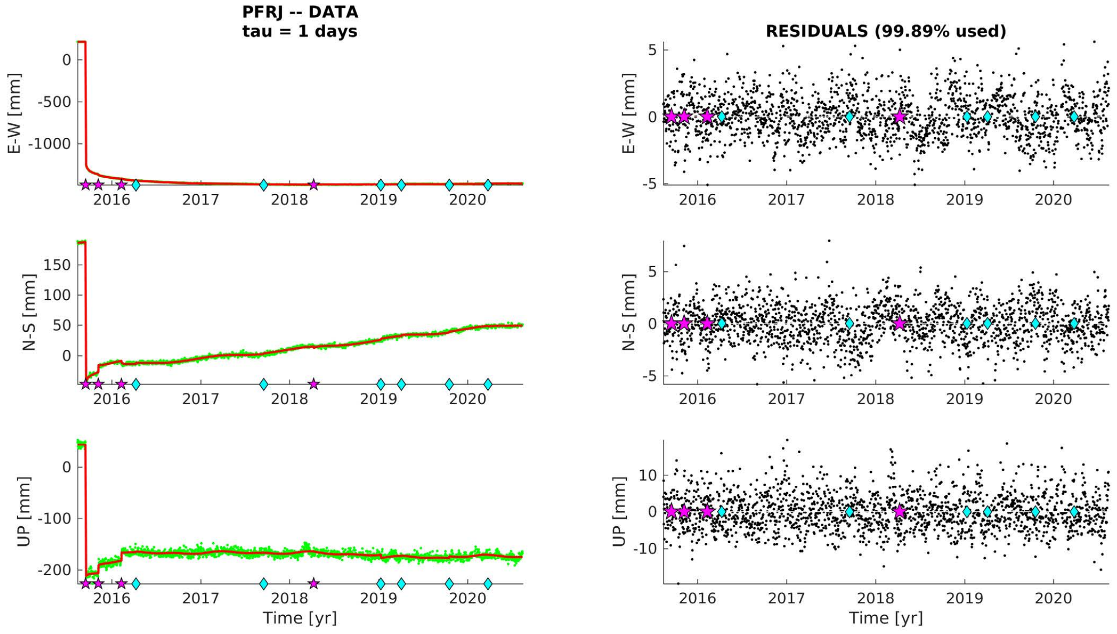

Figure S60. Series of figures showing the GNSS time-series (left column) for east, north and vertical component and the model residuals (right column). The red lines represent the predictions of the trajectory model. The red lines and the green dots represent the predictions of the trajectory model and the daily observations, respectively. The model accounts for a linear, interseismic rate, antenna offsets (Cyan diamond symbol), earthquake offsets (fuchsia stars), postseismic decays, and seasonal oscillations.

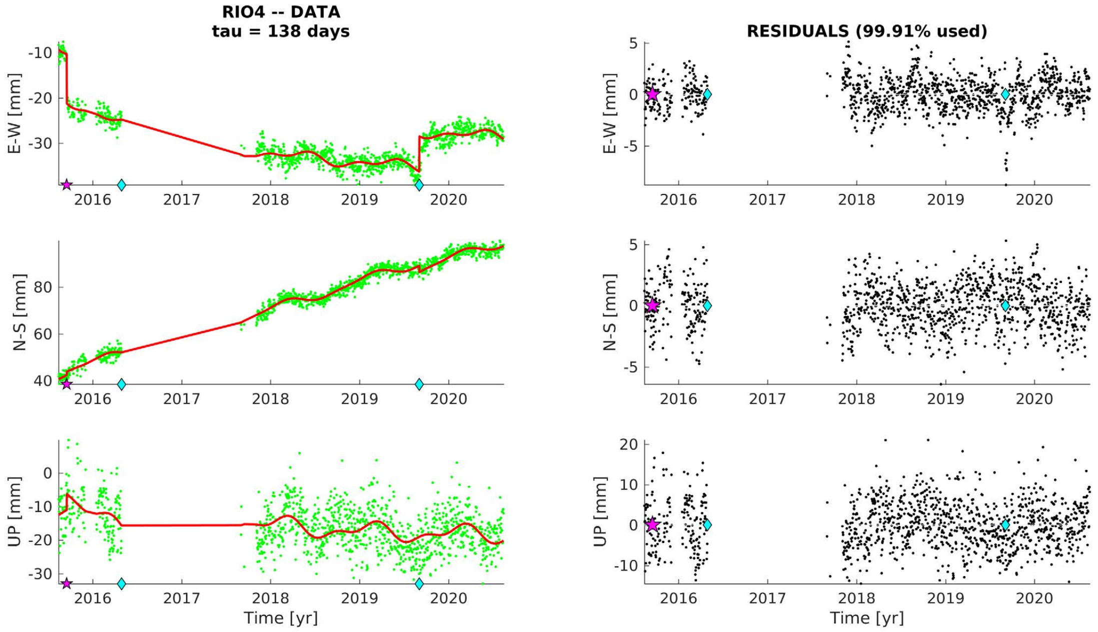

Figure S61. Series of figures showing the GNSS time-series (left column) for east, north and vertical component and the model residuals (right column). The red lines represent the predictions of the trajectory model. The red lines and the green dots represent the predictions of the trajectory model and the daily observations, respectively. The model accounts for a linear, interseismic rate, antenna offsets (Cyan diamond symbol), earthquake offsets (fuchsia stars), postseismic decays, and seasonal oscillations.

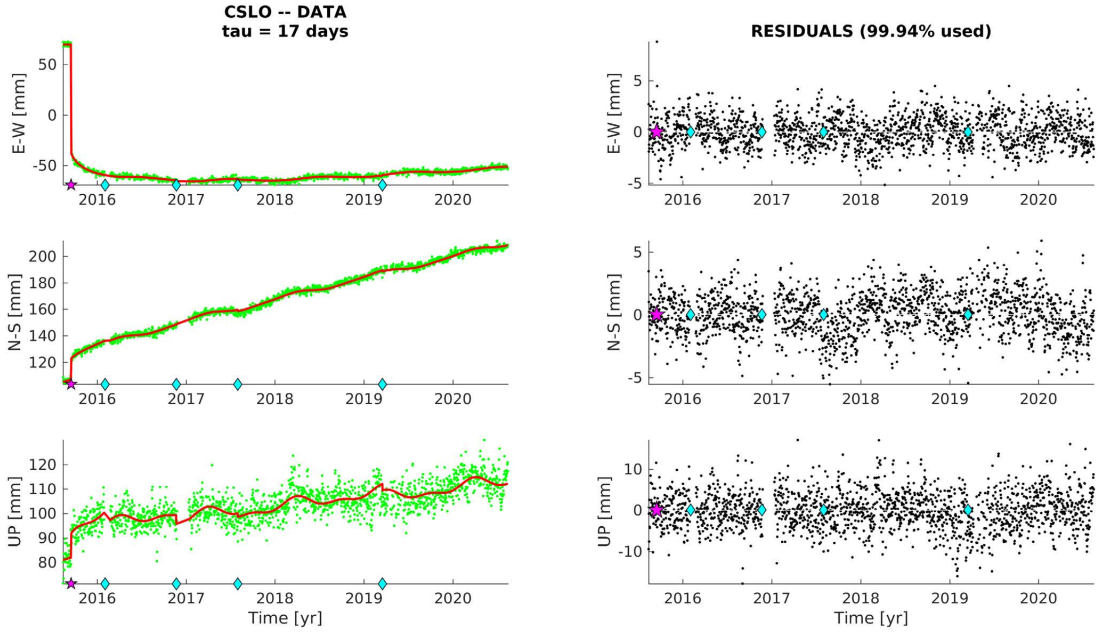

Figure S62. Series of figures showing the GNSS time-series (left column) for east, north and vertical component and the model residuals (right column). The red lines represent the predictions of the trajectory model. The red lines and the green dots represent the predictions of the trajectory model and the daily observations, respectively. The model accounts for a linear, interseismic rate, antenna offsets (Cyan diamond symbol), earthquake offsets (fuchsia stars), postseismic decays, and seasonal oscillations.

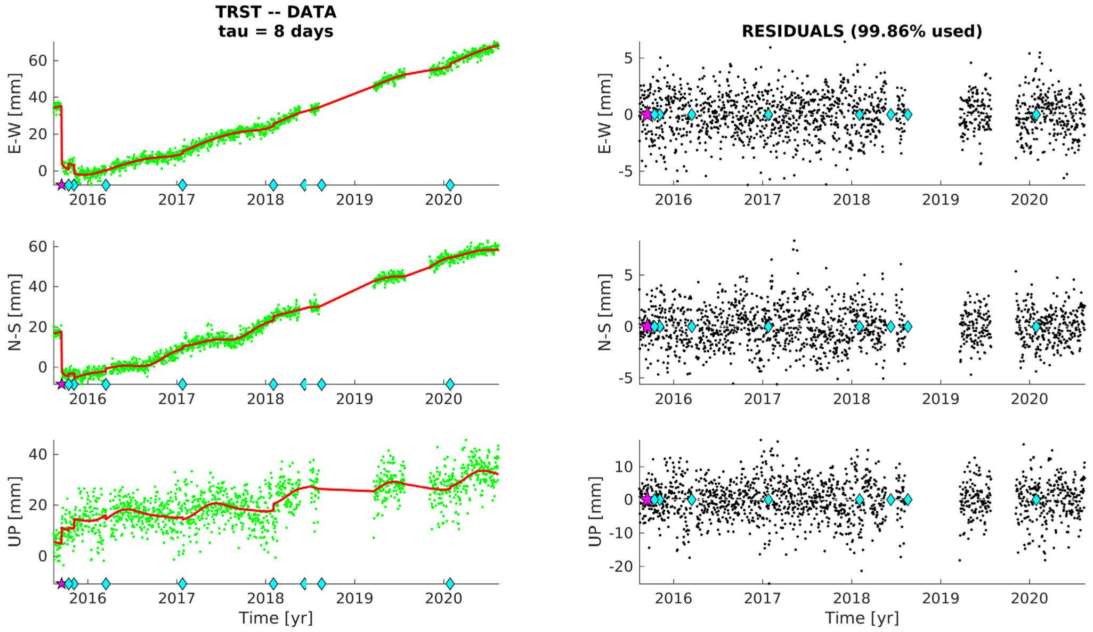

Figure S63. Series of figures showing the GNSS time-series (left column) for east, north and vertical component and the model residuals (right column). The red lines represent the predictions of the trajectory model. The red lines and the green dots represent the predictions of the trajectory model and the daily observations, respectively. The model accounts for a linear, interseismic rate, antenna offsets (Cyan diamond symbol), earthquake offsets (fuchsia stars), postseismic decays, and seasonal oscillations.
